# Efficient Synthesis of DNA Duplexes Containing Reduced Acetaldehyde Interstrand Cross-Links

**DOI:** 10.1021/jacs.2c10070

**Published:** 2022-12-30

**Authors:** Sally B. Morton, L. David Finger, Roxanne van der Sluijs, William D. Mulcrone, Michael Hodskinson, Christopher L. Millington, Christina Vanhinsbergh, Ketan J. Patel, Mark J. Dickman, Puck Knipscheer, Jane A. Grasby, David M. Williams

**Affiliations:** † Centre for Chemical Biology, Department of Chemistry, Sheffield Institute for Nucleic Acids, University of Sheffield, Brook Hill, Sheffield S3 7HF, U.K.; ‡ Oncode Institute, Hubrecht Institute−KNAW and University Medical Center Utrecht, Uppsalalaan 8, 3584 CT Utrecht, The Netherlands; § MRC Laboratory of Molecular Biology, Francis Crick Avenue, Cambridge CB2 0QH, U.K.; ∥ Department of Chemical and Biological Engineering, University of Sheffield, Mappin Street, Sheffield S1 3JD, U.K.

## Abstract

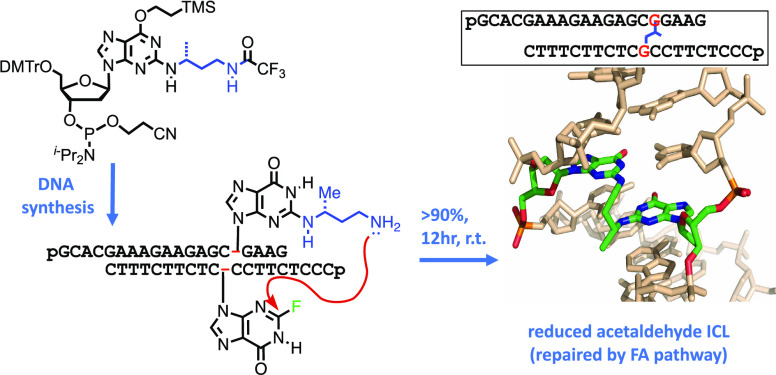

DNA interstrand cross-links (ICLs) prevent DNA replication and transcription and can lead to potentially lethal events, such as cancer or bone marrow failure. ICLs are typically repaired by proteins within the Fanconi Anemia (FA) pathway, although the details of the pathway are not fully established. Methods to generate DNA containing ICLs are key to furthering the understanding of DNA cross-link repair. A major route to ICL formation *in vivo* involves reaction of DNA with acetaldehyde, derived from ethanol metabolism. This reaction forms a three-carbon bridged ICL involving the amino groups of adjacent guanines in opposite strands of a duplex resulting in amino and imino functionalities. A stable reduced form of the ICL has applications in understanding the recognition and repair of these types of adducts. Previous routes to creating DNA duplexes containing these adducts have involved lengthy post-DNA synthesis chemistry followed by reduction of the imine. Here, an efficient and high-yielding approach to the reduced ICL using a novel *N*^2^-((*R*)-4-trifluoroacetamidobutan-2-yl)-2′-deoxyguanosine phosphoramidite is described. Following standard automated DNA synthesis and deprotection, the ICL is formed overnight in over 90% yield upon incubation at room temperature with a complementary oligodeoxyribonucleotide containing 2-fluoro-2′-deoxyinosine. The cross-linked duplex displayed a melting transition 25 °C higher than control sequences. Importantly, we show using the Xenopus egg extract system that an ICL synthesized by this method is repaired by the FA pathway. The simplicity and efficiency of this methodology for preparing reduced acetaldehyde ICLs will facilitate access to these DNA architectures for future studies on cross-link repair.

## Introduction

Interstrand DNA cross-links (ICLs), where two DNA strands are covalently linked, are potentially the most lethal type of DNA lesion. ICLs block DNA strand separation, preventing both replication and transcription.^1^ Failure to remove ICLs results in a significantly enhanced susceptibility to cancer, bone marrow failure, and growth abnormalities, which are characteristic phenotypes of patients suffering from Fanconi Anemia (FA).^2^ FA patients have mutations in any of at least 21 genes associated with the FA repair pathway. In healthy individuals, proteins involved in the FA pathway promote incision of the ICL DNA to “unhook” the adducted nucleotide, allowing subsequent replicative bypass of the lesion by translesion (TLS) polymerases, coupled with homologous recombination.^2^

**Scheme 1:**
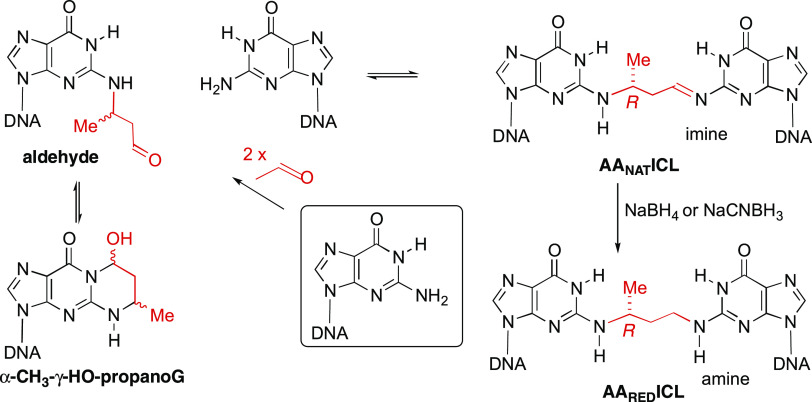
Formation of AA_NAT_ICL and AA_RED_ICL

ICLs can be formed through reaction of DNA with exogenous mutagens like cisplatin and mitomycin C or metabolic aldehydes such as acetaldehyde. Acetaldehyde, which is produced endogenously from the oxidation of ethanol by alcohol dehydrogenase, is found in tobacco smoke and in many fruits and vegetables.^3,4^ Acetaldehyde-derived ICLs (AA ICLs) are most commonly formed between exocyclic amino groups of two adjacent guanines on opposite strands of the DNA duplex in a 5′-CpG-3′ sequence.^5^ These AA ICLs, which result from the condensation of DNA with two molecules of acetaldehyde, are comprised of a methylated 3-carbon bridge with an amino and imino terminus. A stereochemical preference for the *R* configuration has been observed in duplexes containing this ICL (Scheme 1).^5−7^

Previous studies have shown that DNA duplexes containing these ICLs (AA_NAT_ICL) can be reduced with NaCNBH_3_ or NaBH_4_.^5,7^ Duplexes containing these reduced cross-links (AA_RED_ICL) have provided a valuable, stable surrogate of the natural cross-link for studying the repair of AA ICLs.^5−8^ Importantly, unlike AA_NAT_ICL, which was repaired by both an FA and a non-FA pathway,^8^ AA_RED_ICL was exclusively repaired by the FA pathway, considerably reducing ambiguity during studies of ICL repair.

**Scheme 2:**
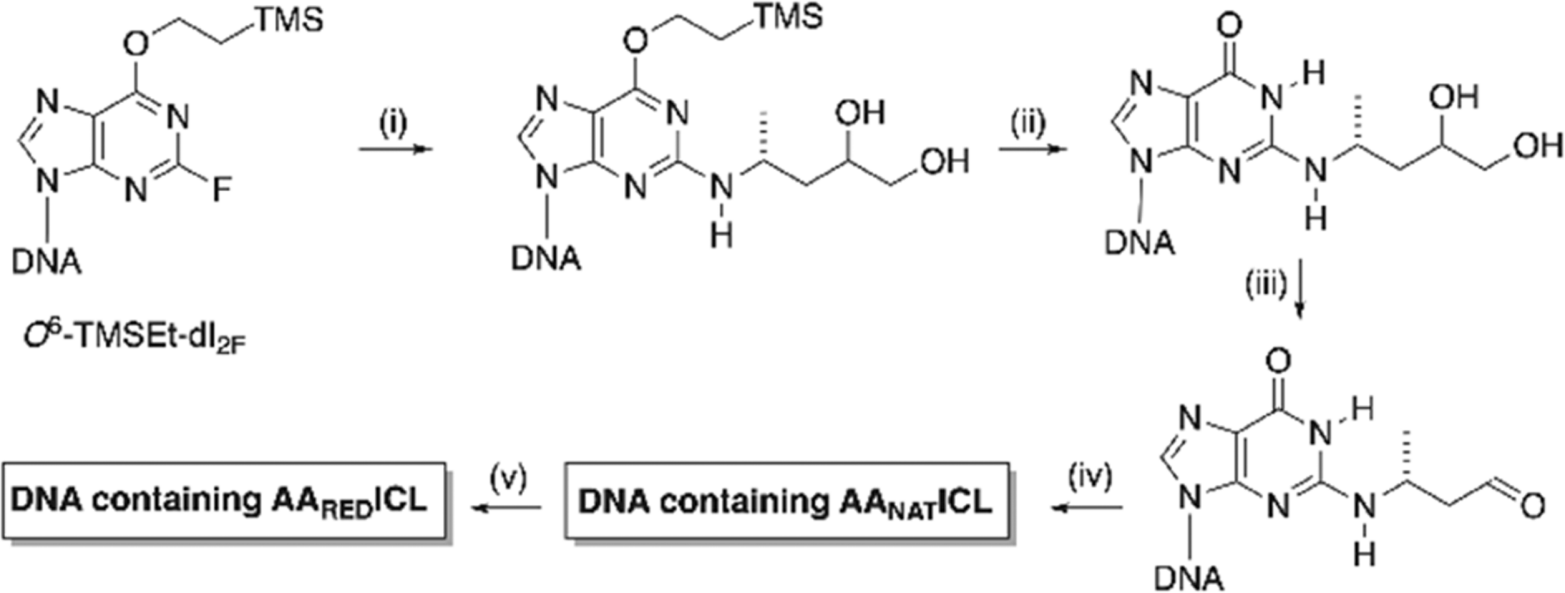
Synthesis of the DNA Duplex Containing AA_RED_ICL^5,7^ Reagents and conditions: (i) 4-(*R*)-aminopentane-1,2-diol, DIPEA, DMSO, heat, 24 h; (ii) 5% AcOH; (iii) aq NaIO_4_; (iv) ODN complement, buffer, 1 week; and (v) NaBH_4_ or NaCNBH_3_.

The synthesis of duplexes containing the AA_NAT_ICL was achieved previously by post-DNA synthesis methods (Scheme 2).^5,9^ Thus, an oligodeoxyribonucleotide (ODN) containing *O*^6^-(trimethylsilylethyl)-2-fluoro-2′-deoxyinosine (*O*^6^-TMSEt-dI_2F_) was reacted with 4-(*R*)-aminopentane-1,2-diol (Scheme 2i), which in turn was synthesized separately in seven steps.^5^ To remove the *O*^6^-protecting group, the product of the reaction was treated with aqueous acetic acid (Scheme 2ii). The *O*^6^-protecting group prevents side reactions during DNA synthesis and increases the rate of fluoride displacement.^6,10,11^ Subsequent treatment of the modified ODN with aq NaIO_4_ generated an aldehyde (Scheme 2iii).^6,10^ Although in equilibrium with the aldehyde, the preferred configuration of this product was the cyclized propanoG (Scheme 1). Upon addition of the complementary strand, the aldehyde slowly formed a cross-link with the 2-amino group of the adjacent dG residue in the CpG step (Scheme 2iv). For the ODN bearing the *R* configuration of propanoG (6*R*, 8*S*), the extent of cross-linking to form the AA_NAT_ICL after 21 days was ∼38%. In contrast, the extent of cross-link formation for the *S*-diastereoisomer (6*S*, 8*R*) was only 5%.^5^ The reduced form of the cross-link (AA_RED_ICL) was prepared by extended treatment with NaCNBH_3_ or NaBH_4_ (Scheme 2v).^5,7,12^

We envisaged a more direct synthetic route in which a site-specific AA_RED_ICL could be prepared without time-consuming post-DNA synthesis manipulation. Here, we report the synthesis of a bespoke phosphoramidite and show that it can be incorporated into an ODN using routine automated DNA synthesis. After deprotection and purification using standard procedures, the ODN containing *N*^2^-((*R*)-4-aminobutan-2-yl)-2′-deoxyguanosine (dG_AB_) can be annealed to a suitably designed ODN complement containing 2-fluoro-2′-deoxyinosine (dI_2F_) to produce the AA_RED_ICL directly and efficiently.

## Results and Discussion

**Scheme 3:**
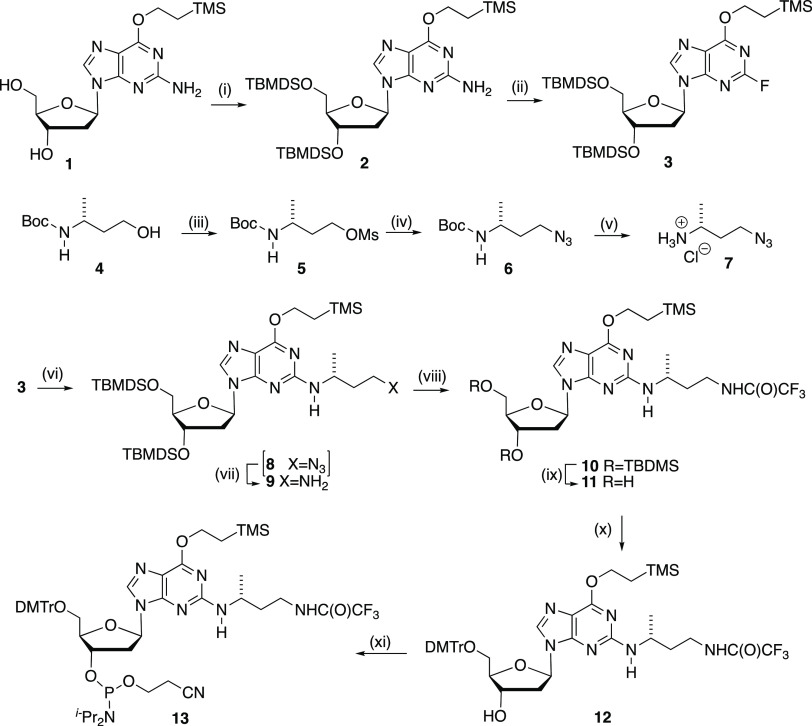
Synthesis of the Novel *N*^2^-((*R*)-4-Aminobutan-2-yl)-dG Phosphoramidite **13** Reagents and conditions: (i) TBDMSCl, imidazole, DMF, 94%; (ii) *tert-*butyl nitrite, PVPHF, toluene, −10 °C, 56%; (iii) MsCl, Et_3_N, DCM, 100%; (iv) NaN_3_, DMSO, heat, 68%; (v) HCl in DCM, dioxan, 88%; (vi) *i*-PrOSiMe_3_, DIPEA, DMSO heat, 13 d, then (vii) H_2,_ Pd-C, EtOAc, 41% (viii) MeO(CO)CF_3_, DCM 82%; (ix) Et_3_N·3HF, THF, 79%; (x) DMTrCl, DMAP, pyridine, Et_3_N, 52%; and (xi) (*i-*Pr_2_N)_2_POCH_2_CH_2_CN, 5-(thioethyl)tetrazole, dry DCM, 89%.

Our synthesis of the novel phosphoramidite began with the *O*^6^-protected 2′-deoxyguanosine **1**(10) (Scheme 3). Previously, compound **1** was converted to the corresponding 2-fluoroinosine by nitrosation followed by treatment with pyridine/HF.^10^ Decorte et al.^10^ reported some variability in the yields of this procedure, which was also our experience. Instead, we opted to use polyvinylpyridinium poly(hydrogen fluoride) (PVPHF), a polymer-supported HF that has been shown to be relatively safe and easy to remove following fluorination.^13^ Because the preferred solvent for using PVPHP is toluene, we first protected the 3′ and 5′ hydroxyl groups of **1** as TBDMS ethers. Treatment of the protected nucleoside **2** with *tert*-butyl nitrite followed by PVPHF at −10 °C for 15 min gave the fluorinated nucleoside **3** in a 56% yield after silica chromatography. We attribute this moderate yield to small amount of desilylation and other more polar products observed during this reaction in agreement with the literature.^13^ To introduce the aminobutyl unit, our design strategy was to prepare a phosphoramidite that, following DNA synthesis, would allow the less hindered and more nucleophilic amino group to form the DNA duplex ICL. Thus, starting from commercially available (*R*)-3-aminobutan-1-ol, we first protected the amino terminus with the Boc group to give **4**. Compound **4** was then converted into azide **6***via* mesylate **5**. Removal of the Boc-protecting group from **6** provided **7**, isolated as its hydrochloride salt.

Initial attempts to introduce the azidobutyl chain to nucleoside **3** by heating with **7** in DMSO in the presence of a base (DIPEA) resulted in significant desilylation. Attributing this to fluoride ions generated during the displacement reaction, we added a fluoride ion scavenger, isopropoxytrimethylsilane, allowing the smooth transformation to nucleoside **8** without concomitant desilylation. Unfortunately, we were unable to purify **8** due to its coelution on silica TLC with nucleoside **3**. Instead, crude **8** was reduced directly to nucleoside **9** using catalytic hydrogenation to afford the product in 41% yield (from compound **3**) following silica chromatography. Trifluoroacetyl protection of the amino group of nucleoside **9** gave **10**, which was then treated with triethylamine HF to furnish **11**. Protection of the 5′-hydroxyl group with DMTr gave **12**, which following phosphitylation afforded phosphoramidite **13** in 89% yield as a 1:1 mixture of two diastereoisomers.

**Scheme 4:**
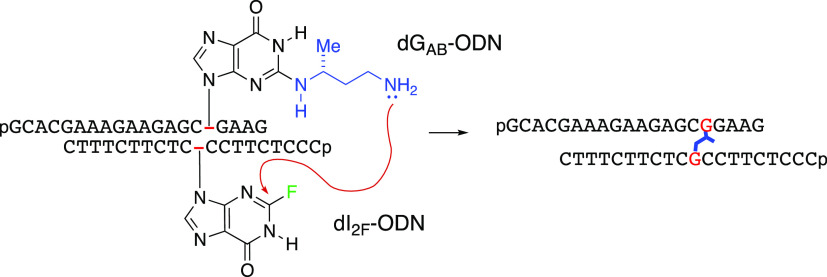
Synthesis of the DNA Duplex Containing AA_RED_ICL

The sequences chosen for the cross-linked duplex have been described previously (Scheme 4).^8^ Phosphoramidite **13** was used in standard automated DNA synthesis using base-labile phosphoramidites (phenoxyacetyl (pac) for dA and dG and acetyl for dC) to prepare the requisite oligonucleotide containing the *N*^2^-((*R*)-4-trifluoroacetamidobutan-2-yl)-dG modification. Following DNA synthesis, the column was treated with 10% diethylamine in acetonitrile to remove the cyanoethyl-protecting groups. Subsequent cleavage from the solid support and deprotection was achieved using conc aq ammonia solution for 6 h at room temperature. The oligonucleotide containing the modified nucleoside, *N*^2^-((*R*)-4-aminobutan-2-yl)-2′-deoxyguanosine (dG_AB_-ODN), was then purified by reverse-phase ion-pairing HPLC (RP-IP-HPLC) using a mobile phase gradient comprised of triethylammonium acetate pH 7 (TEAA) buffer and acetonitrile.

LC-MS afforded the mass for the fully deprotected dG_AB_-ODN (Figure S1A), indicating complete removal of the O6-TMSEt group. This presumably occurred due to acidification of the solvent during evaporation of the TEAA buffer solution. The same sequence without the modification was synthesized, deprotected, and purified identically to create a control strand (dG-ODN), which was also confirmed by LC-MS (Figure S1B). The requisite dI_2F_-containing complement strand (dI_2F_-ODN) was prepared using a commercially available O6-TMSEt-protected dI_2F_-phosphoramidite. Following synthesis, the ODN was fully deprotected according to the manufacturer’s protocol, purified by RP-IP-HPLC, and characterized by LC-MS (Figure S1C).^14^ A control ODN same with the dI_2F_ nucleotide replaced by dG (herein referred to as d_GCOMP_-ODN) was also prepared, purified, and confirmed by LC-MS (Figure S1D).

**Figure 1:**
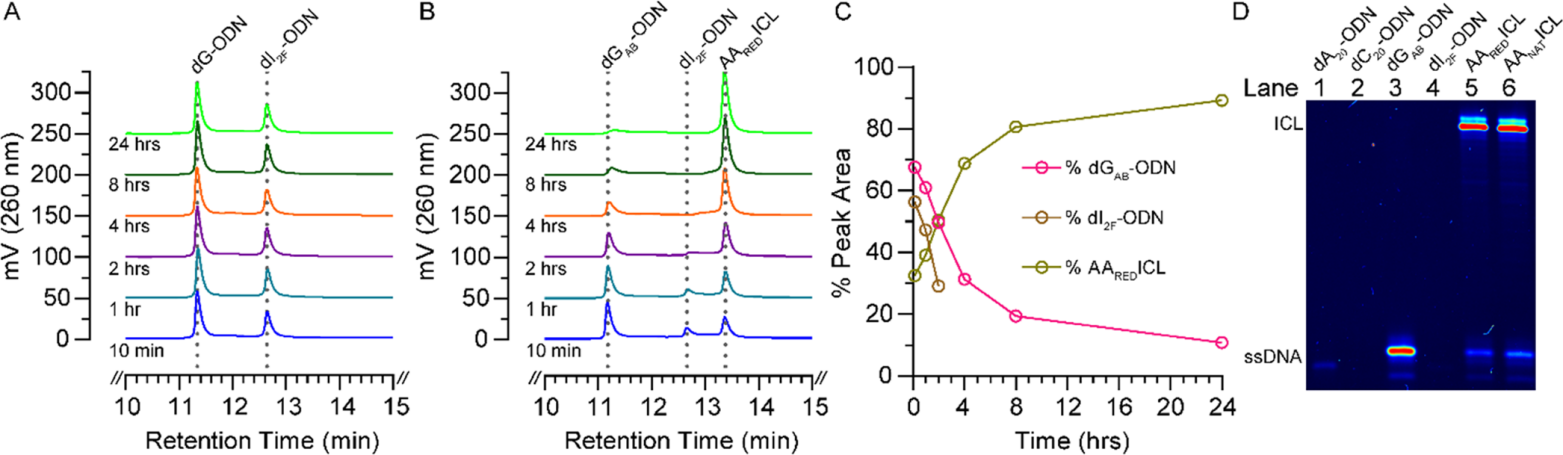
Monitoring cross-link formation by RP-IP-HPLC and denaturing PAGE. (A, B) Portions (10–15 min) of the chromatograms from the RP-IP-HPLC analysis (see the Supporting Information for details) of the quenched reactions, which were used to assess reaction progress. Each chromatogram is sequentially offset by 50 mV (left *Y*-axis) for clarity, and the time of quench is shown below its respective chromatogram (blue—10 min, cyan—1 h, purple—2 h, orange—4 h, green—8 h, bright green—24 h). See Figure S2A–D for full chromatograms and standards. Analysis of the control duplex formed by (A) dG-ODN (11.34 min) and dI_2F_-ODN (12.64 min) shows no change over 24 h, whereas the duplex formed by (B) dG_AB_-ODN (11.18 min) and dI_2F_-ODN (12.64 min) gives rise to a new peak at 13.36 min, which LC-MS has confirmed to be AA_RED_ICL (Figure S1D). (C) Quantitation of the peak areas of the single strands and the AA_RED_ICL of the chromatograms from panel (B) showing that the reaction is almost complete within 24 h (pink—dG_AB_-ODN, brown—dI_2F_-ODN, olive-green—AA_RED_ICL duplex). Note: the dI_2F_-ODN peak could not be quantified after 2 h. (D) Image of a 15% denaturing PAGE stained with SYBR gold that confirms the formation of the AA_RED_ICL. Each lane contains ∼100 ng of the indicated ODN, which is labeled above. The AA_NAT_ICL sample in lane 6, which was prepared as previously reported,^8^ is included for comparison. Note: the low intensities of bands in lanes 1, 2, and 4 are due to the inability of cyanine dyes to bind/stain homopyrimidines or ssDNA composed of just A, C, or T (Figure S2E).^18^

**Figure 2:**
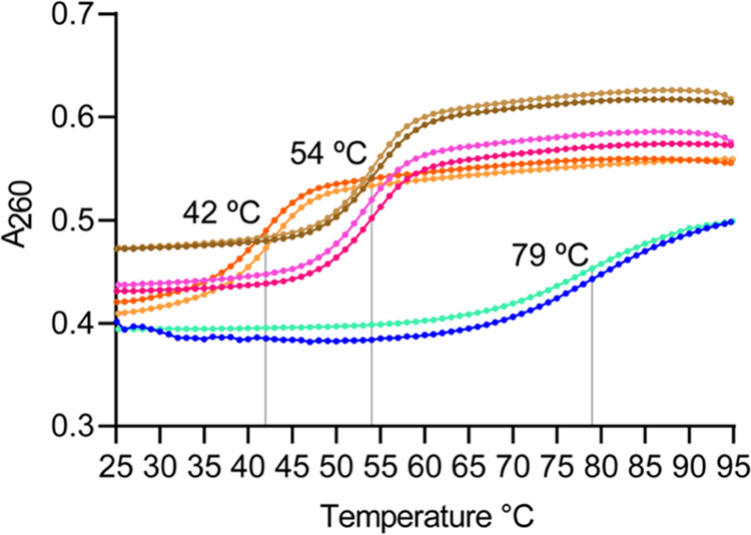
UV thermal melting data^19,20^ of control heteroduplexes (HDs) and AA_RED_ICL. See the Supporting information for experimental details. Forward (25–95 °C; ↑) and reverse (95–25 °C; ↓) melts are shown for dG/dI_2F_-HD (orange ↑, light orange ↓), dG/dG_COMP_-HD (magenta ↑, pink ↓), dG_AB_/dG_COMP_-HD (brown ↑, light brown ↓), and AA_RED_ICL (blue ↑, cyan ↓). The melting temperatures (*T*_m_), which are indicated by the gray lines, are noted on the plot.

For the cross-linking reaction, dI_2F_-ODN (1.1 equiv) was combined with either dG-ODN or dG_AB_-ODN in 50 mM sodium borate pH 9.0 buffer. After annealing for 10 min, the duplex was incubated at room temperature, and the reaction progress was monitored by RP-IP-HPLC (Figures 1A,B and S2A–D). Quantification of the chromatograms showed ∼89% conversion within 24 h (Figure 1C), which was also confirmed by denaturing PAGE (Figures 1D and S2E). The identity of the AA_RED_ICL was confirmed using LC-MS (Figure S1E) after purification by RP-IP-HPLC. Further confirmation of ICL formation was obtained by performing UV thermal melting analyses on various combinations of heteroduplexes and the purified AA_RED_ICL. As seen previously,^15,16^ the presence of a covalent ICL in AA_RED_ICL enhanced the melting temperature (*T*_m_) by ≥25 °C compared to all permutations of heteroduplexes (Figures 2 and S3). Furthermore, unlike AA_NAT_ICL UV thermal melts,^5^ the AA_RED_ICL melting transition is reversible, consistent with a stable ICL.

**Figure 3:**
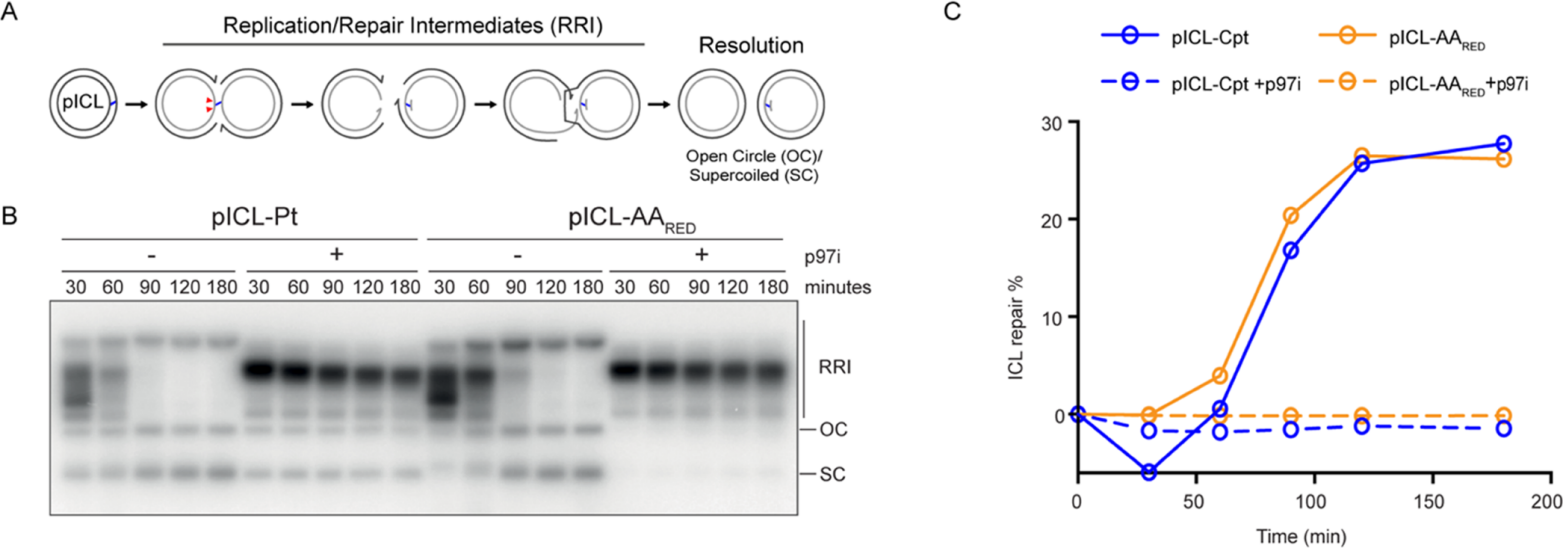
Repair of AA_RED_ICL in *Xenopus* egg extracts. (A) Schematic representation of replication and repair intermediates (RRI) that arise during ICL repair. Red arrowheads indicate nucleolytic incisions during the repair process that unhook the ICL from one of the two strands. (B) Representative gel image generated by autoradiography after electrophoresis, showing the results of *Xenopus* egg extract replication of plasmids containing cisplatin- or reduced-acetaldehyde-induced ICLs (pICL-Pt and pICL-AA_RED_, respectively) in the presence of ^32^P-α-dCTP and with or without p97i. Replication and repair intermediates (RRI), open circle (OC), and supercoiled (SC) products are indicated. Due to contaminating non-cross-linked plasmids in pICL-Pt, some OC and SC products arise at early times, and in the presence of p97i. (C) Quantification of ICL repair with respect to time based on the assay schematic and gel in Figure S4A,B and as described in the supporting methods.

To investigate whether AA_RED_ICLs prepared through this synthetic route are repaired by the FA pathway, we ligated the AA_RED_ICL duplex (Scheme 4) into a plasmid (pICL-AA_RED_). This plasmid was replicated in Xenopus egg extract alongside a plasmid containing a cisplatin ICL (pICL-Pt), the repair of which has been shown to fully rely on the FA pathway.^17^ Replication of pICL-Pt, as reported, resulted in replication fork convergence at the ICL followed by the generation of a characteristic pattern of replication and repair intermediates (RRI) of the FA pathway and accumulation of resolved open circular and supercoiled products (OC and SC, Figure 3A,B). Replication of pICL-AA_RED_ resulted in a similar pattern of repair intermediates. Addition of an inhibitor of the 97 segregase (p97i), which has been shown to prevent unloading of the CMG helicase, a crucial step in the FA pathway, resulted in the accumulation of RRI intermediates, indicating that repair was abrogated in both pICL-Pt and pICL-AA_RED_. To show directly that repair of pICL-AA_RED_ requires the FA pathway, we utilized the *Not*I assay^8^ that measures repair products generated by translesion synthesis and homologous recombination in the FA pathway (Figure S4A,B). Quantification of repair products (Figure 3C) showed that both Pt-ICL and AA_RED_ICL are repaired with similar efficiency and that repair is completely abolished by p97i. This indicates that repair of both cross-links relies on the FA pathway.

In summary, the synthesis of duplex AA_RED_ICL can be achieved in high yields without complex or time-consuming post-synthesis manipulation. We have confirmed that a plasmid containing the ICL duplex synthesized in this way is repaired by the FA pathway. The novel methodology described herein will facilitate future structural, biochemical, and cellular studies of the FA pathway by providing easy access to site-specific, stable ICLs caused by endogenous mutagens like acetaldehyde.

## Supporting Information Available

Experimental procedures, supporting figures, MS spectra of oligodeoxyribonucleotides, and NMR spectra of compounds (PDF)

## Abbreviations Used

ICL: interstrand cross-link
FA: Fanconi Anemia
AA_NAT_ICL: acetaldehyde-derived native interstrand cross-link
AA_RED_ICL: acetaldehyde-derived reduced interstrand cross-link
ODN: oligodeoxyribonucleotide
PVPHF: polyvinylpyridinium poly(hydrogen fluoride)
TBDMS: *tert*-butyldimethylsilyl
DIPEA: *N*,*N*-diisopropylethylamine
DMTr: dimethoxytrityl
dG_AB_: *N*^2^-((*R*)-4-aminobutan-2-yl)-2′-deoxyguanosine
dI_2F_: 2-fluoro-2′-deoxyinosine
TMSEt: trimethylsilylethyl
TEAA: triethylammonium acetate
TLC: thin layer chromatography
AcOH: acetic acid
p97i: p97 segregase inhibitor
pICL-Pt: plasmid containing cisplatin ICL
pICL-AA_RED_: plasmid containing AA_RED_ICL
RRI: replication and repair intermediates
OC: open circle
SC: supercoiled
