# Supplementary material for: Efficient Synthesis of DNA Duplexes Containing Reduced Acetaldehyde Interstrand Cross-Links
Source: J Am Chem Soc. 2022 Dec 30;145(2):953–9. doi: 10.1021/jacs.2c10070 (PMC9853853; doi:10.1021/jacs.2c10070)

Supporting Information

An efficient synthesis of DNA duplexes containing reduced acetaldehyde interstrand crosslinks.

Sally B. Morton[1], L. David Finger[1], Roxanne van der Sluijs[2], William D. Mulcrone[1], Michael Hodskinson[3†], Christopher L. Millington[1,††], Christina Vanhinsbergh[1], Ketan J. Patel[3,††], Mark J. Dickman[4], Puck Knipscheer[2], Jane A. Grasby[1] and David M. Williams[1*]

[1]Centre for Chemical Biology, Department of Chemistry, Sheffield Institute for Nucleic Acids, University of Sheffield, Sheffield S3 7HF, UK.

[2]Hubrecht Institute–KNAW and University Medical Center Utrecht, 3584 CT Utrecht, The Netherlands.

[3]MRC Laboratory of Molecular Biology, Francis Crick Avenue, Cambridge CB2 0QH, UK.

[4]Department of Chemical and Biological Engineering, University of Sheffield, Sheffield S1 3JD, UK.

†Present address: Evonetix Ltd, 9a Coldham's Business Park, Norman Way, Cambridge CB1 3LH, UK.

††Present address: MRC Weatherall Institute of Molecular Medicine, University of Oxford, John Radcliffe Hospital, Headington, Oxford OX3 9DS.

*Corresponding author: d.m.williams@sheffield.ac.uk

Contents

Chemical Synthesis pages 2–16.

Oligodeoxyribonucleotide (ODN) methods pages 17-20

DNA replication assays methods page 21

Supplementary Figures pages 22-24

References pages 26

NMR spectra of compounds pages 27-54

Chemical Synthesis

All reagents were purchased from commercial suppliers with no further purification, unless otherwise stated. Anhydrous DIPEA, DIPA, cyanoethanol and PCl_3 were all further distilled before use. DMTrCl and TsCl were recrystallized before use. Anhydrous DMSO and ethanol were dried and stored over molecular sieves. All other dry solvents were obtained from a Grubbs apparatus using oven dried glassware under N_2 using standard Schlenk techniques. All anhydrous reactions were carried out under N_2 using oven dried glassware, with exception of the reaction with PVPHF (38% - 42% HF), which was carried out in high density polyethylene bottles purchased from Scientific Laboratory Supplies Ltd. PVPHF was purchased from Sigma Aldrich.

Flash column chromatography was performed on silica gel (30-70m) supplied by Merck. Thin layer chromatography (TLC) was performed on pre-coated 60 F254 aluminium backed plates. TLC plates were visualised under UV (254nm) and/or the following stains: anisaldehyde stain (visualising nucleosides), ceric ammonium molybdate stain (identifying compounds rich in hydroxyl groups), potassium permanganate stain (visualising oxidizable species) and Ninhydrin stain (visualising amines/azides).

Some analytical RP-HPLC was performed on a Waters 2695 or 2690 instrumentation using an Gemini C18, 5 µm, 4.6 x 250 nm column (Phenomenex), flow rate 1mL/min, UV detection at 260nm. Larger purifications were achieved using a Phenomenex semi-preparative Gemini C18 (5 µm, 10 x 250 mm) column at 5 mL/min or a Phenomenex preparative Gemini C18 (5 µm, 19 x 250 mm) column at a flow rate of 17 mL/min, UV detection at 260 nm.

3',5'-Di-*O*-*tert*-butyldimethylsilyl-*O*^{6}-(2-trimethylsilylethyl)-2'-deoxyguanosine (2)

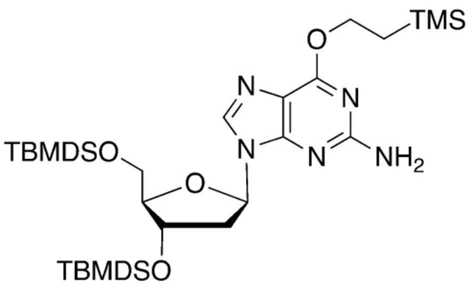

To *O*^{6}-(2-Trimethylsilylethyl)-2'-deoxyguanosine (**1**)[1] (0.91 g, 2.5 mmol) in anhydrous DMF (7 mL) were added imidazole (0.84 g, 12.4 mmol) and TBDMSCl (0.93 g, 6.2 mmol). The mixture was then stirred under N_2 at room temperature for 2.5 h. The solvent was then removed by evaporation. The resultant residue was re-dissolved in EtAOc (10 mL) and extracted with water (3 × 50 mL). The water layers were combined and back-extracted with EtOAc (100 mL). The combined organic layers were dried ($MgSO_4$), and the solvent evaporated to yield a white solid (1.39 g, 2.3 mmol, 94%).

TLC: R_f (20 % EtOAc/ pet. ether) = 0.56

^{1}H NMR (400 MHz, CD_3OD) δ 8.02 (s, 1H, C*H*, H8); 6.33 (t, *J* = 6.6 Hz, 1H, C*H*, H1'); 4.69 – 4.64 (m, 1H, C*H*, H3'); 4.61 (t, 2H, SiCH_2); 3.98 (dd, *J* = 7.2, 3.7 Hz, 1H, C*H*, H4'); 3.83 (qd, *J* = 11.2, 4.1 Hz, 2H, CH_2, H5'); 2.71 (dt, *J* = 13.0, 6.4 Hz, 1H, C*H*, H2'); 2.41 (ddd, *J* = 13.2, 6.0, 3.7 Hz, 1H, C*H*, H2'); 1.24 (t, 2H, OCH_2); 0.97 (s, 9H, (CH_3)$_3$); 0.93 (s, 9H, (CH_3)$_3$); 0.17 (s, 6H, (CH_3)$_2$); 0.13 – 0.08 (m, 15H, (CH_3)$_3$ & (CH_3)$_2$).

^{13}C NMR (101 MHz, CD_3OD) δ 161.02, 160.44, 153.12, 137.33, 114.18, 87.85, 83.68, 72.23, 64.33, 62.70, 40.13, 25.06, 24.88, 17.86, 17.48, 17.02, -2.79, -5.89, -5.99, -6.67, -6.72.

HRMS (ESI+) Found 596.3498 $[M+H]^+$ (Calculated for $C_{27}H_{53}N_5O_4Si_3$ $[M+H]^+$ 596.3478).

3',5'-Di-*O*-*tert*-butyldimethylsilyl-*O*^{6}-(2-trimethylsilylethyl)-2-fluoro-2'-deoxyinosine (3)

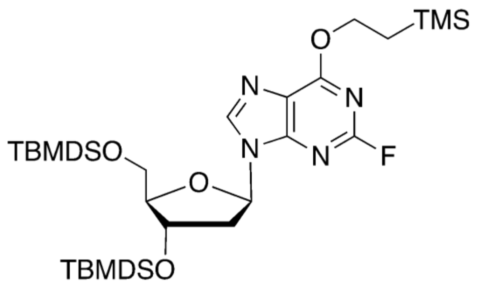

3',5'-Di-*O*-*tert*-butyldimethylsilyl-*O*^{6}-(2-trimethylsilylethyl)-2'-deoxyguanosine (**2**) (1.00 g, 1.7 mmol) was stirred in anhydrous toluene (30 mL) at -10 °C under N_2 at room temperature in a high-density polyethylene bottle. To this solution, *tert*-Butyl-nitrite (0.80 mL, 6.7 mmol) and PVPHF (4.20 g, 38–42 % HF) were carefully added. The mixture was stirred at -10 °C for 15 min, and was then left to warm to 0 °C over 15 min. The reaction was neutralised by the addition of aqueous $NaHCO_3$ (200 mL, 0.4M) and then filtered through celite, which was sequentially washed with aqueous $NaHCO_3$ (50 mL, 0.4M), water (50 mL) and EtOAc (200 mL). After separation, the organic layer was dried ($MgSO_4$) and evaporated. The crude product was then purified by flash column chromatography (20% EtOAc/pet. ether) and evaporated to give a golden-coloured oil (2.24 g, 3.7 mmol, 56%).

TLC: R_f (20% EtOAc/pet. ether) product = 0.78

^{1}H NMR (400 MHz, CD_3OD) δ 8.37 (s, 1H, C*H*, H8); 6.37 (t, *J* = 6.3 Hz, 1H, C*H*, H1'); 4.81 (dd, *J* = 10.0, 4.6 Hz, 1H, C*H*, H3'); 4.73 (dd, *J* = 9.4, 7.1 Hz, 2H, SiC*H₂*); 3.98 (dd, *J* = 8.0, 3.9 Hz, 1H, C*H*, H4'); 3.86 (ddd, *J* = 14.7, 11.2, 4.1 Hz, 2H, C*H*$_2$, H5'); 2.92 (dt, *J* = 12.9, 6.0 Hz, 1H, C*H*, H2'); 2.47 (ddd, *J* = 13.0, 6.5, 4.8 Hz, 1H, C*H*, H2'); 1.29 (t, *J* = 8.8, 8.1 Hz, 2H, OC*H*$_2$); 0.97 (s, 9H, (C*H*$_3$)$_3$); 0.87 (s, 9H, (C*H*$_3$)$_3$); 0.18 (s, 6H, (C*H*$_3$)$_2$); 0.13 (s, 9H, (C*H*$_3$)$_3$); 0.07 (s, 3H, C*H*$_3$); 0.03 (s, 3H, C*H*$_3$).

^{19}F NMR (377 MHz, CD_3OD) δ -51.8 (1F, N=C-F).

^{13}C NMR (101 MHz, CD_3OD) δ 159.1, 157.0, 152.3, 142.2, 110.3, 87.8, 84.9, 71.6, 66.4, 62.2, 39.3, 25.0, 17.8, 17.5, 17.0, -2.9, -5.9, -6.0, -6.8.

HRMS (ESI+) Found 599.3297 $[M+H]^+$ (Calculated for $C_{27}H_{51}FN_4O_4Si_3$ $[M+H]^+$ 599.3275), Found 621.3106 $[M+Na]^+$ (Calculated for $C_{27}H_{51}FN_4O_4Si_3$ $[M+H]^+$ 621.3094

(*R*)-*Tert*-butyl(4-hydroxybutan-2-yl)carbamate (4)

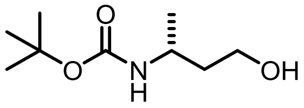

To (*R*)-3-aminobutan-1-ol (1.00 g, 11.2 mmol) dissolved in anhydrous DCM (10 mL), dry triethylamine (2.35 mL, 16.8 mmol) was added at room temperature under N_2. Di-*tert*-butyl dicarbonate (2.83 mL, 12.3 mmol) was then added dropwise over 5 min with vigorous stirring. The solution was then stirred overnight at room temperature under N_2. Reaction progression was monitored by TLC. When complete, the solvent was removed by evaporation. The resultant residue was dissolved in EtOAc (100 mL) and extracted against H_2O (3 x 30 mL). The aqueous layers were combined and back-extracted with EtOAc (50 mL). The combined organic layers were washed with brine (30 mL), dried ($MgSO_4$) and evaporated to afford a white solid (2.11 g, 11.1 mmol, 100%).

TLC: R_f (50% EtOAc/pet. ether) product = 0.46

^{1}H NMR (400 MHz, d6-DMSO) δ 6.65 (d, *J* = 8.2 Hz, 1H, N*H*); 4.36 (t, *J* = 5.1 Hz, 1H, O*H*); 3.62 – 3.45 (m, 1H, C*H*); 3.43 – 3.32 (m, 2H, CH_2OH); 1.60 – 1.39 (m, 2H, CH_2); 1.37 (s, 9H, $(CH_3)_3$); 1.00 (d, *J* = 6.6 Hz, 3H, CH_3).

^{13}C NMR (101 MHz, d6-DMSO) δ 155.5, 77.7, 58.5, 43.6, 28.7, 21.4.

HRMS (ESI+) Found 212.1265 $[M+Na]^+$ (Calculated for $C_9H_{19}NO_3$ $[M+Na]^+$ 212.1257)

IR (ATR, cm^{-1}) 3284 (OH, NH secondary amine, br), 1672 (C=O, secondary amide), 1163 (C-O, carbamate stretch), 1075 (C-O, primary alcohol).

(*R*)-3-((*tert*-Butoxycarbonyl)amino)butyl methanesulfonate (5)

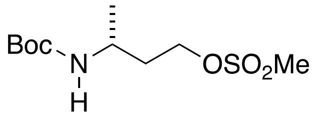

To a stirred solution of (*R*)-*tert*-butyl(4-hydroxybutan-2-yl)carbamate (**4**) (5.67 g, 30.0 mmol) and anhydrous triethylamine (6.70 mL, 47.9 mmol) in anhydrous CH_2Cl_2 (40 mL) at 0 ℃ was added methanesulfonyl chloride (2.78 mL, 36.0 mmol) dropwise. The mixture was stirred at room temperature for 2 h before it was washed with saturated $NaHCO_3$ solution (3 x 30 mL). The organic layer was then separated, dried over K_2CO_3, and concentrated *in vacuo* to give (*R*)-3-((*tert*-Butoxycarbonyl)amino)butyl methanesulfonate (**5**) (8.00 g, 100%) as a yellow solid.

TLC: R_f (EtOAc) product = 0.63

^{1}H NMR (400 MHz, $CDCl_3$) δ 4.47 (d, *J* = 7.0 Hz, 1H, N*H*), 4.26 (dd, *J* = 6.8, 6.0 Hz, 2H, C*H*$_2$OSO$_2$Me), 3.79 (br, 1H, BocNHC*H*), 3.00 (s, 3H, OSO$_2$C*H*$_3$), 1.89 (dtd, *J* = 14.4, 6.8, 5.2 Hz, 1H, BocNHCHCH_AH$_B$), 1.79 (ddt, *J* = 14.4, 8.7, 6.0 Hz, 1H, BocNHCHCH$_A$$H_B$), 1.41 (s, 9H, (C*H*$_3$)$_3$O), 1.16 (d, *J* = 6.7 Hz, 3H, C*H*$_3$).

^{13}C NMR (101 MHz, $CDCl_3$) δ 155.5 (C=O), 110.5 (CH_3), 67.4 (CH_2), 37.4 (CH), 36.6 (CH_2), 28.4 (3 x CH_3), 21.4 (CH_3).

HRMS (ESI+) Found 267.118 $[M+Na]^+$ (Calculated for $[C_{10}H_{21}NO_5S]^+$ $[M+Na]^+$: 267.110)

(*R*)-*tert*-butyl(4-azidobutan-2-yl)carbamate (6)

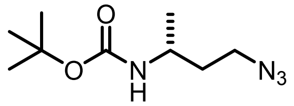

NaN_3 (4.40 g, 67.7 mmol) was added to a stirred solution of mesylate (**5**) (7.79 g, 29.1 mmol) in anhydrous DMSO (40 mL). The mixture was stirred at 70 °C overnight before it was allowed to cool to room temperature. Water (80 mL) was added, then the mixture was extracted into Et_2O (3 x 50 mL). The combined organic layers were washed with brine (3 x 30 mL), dried over $MgSO_4$, and concentrated *in vacuo*. Purification by column chromatography (50/50 EtOAc/ Hexane) gave azide (**6**) (4.03 g, 65%) as a clear oil.

TLC: R_f (30% EtOAc/pet. ether) product = 0.78

^{1}H NMR (δ ^{1}H NMR (400 MHz, CD_3OD) δ 3.80 – 3.54 (m, 1H, C*H*); 3.42 – 3.27 (m, 2H, CH_2N_3); 1.75 – 1.56 (m, 2H, CH_2); 1.46 (s, 9H, (CH_3)$_3$); 1.14 (d, *J* = 6.7 Hz, 3H, CH_3).

^{13}C NMR (101 MHz, CD_3OD) δ 156.4, 78.5, 48.6, 43.9, 35.5, 27.6, 20.2.

HRMS (ESI+) Found 237.1323 $[M+Na]^+$ (Calculated for $C_9H_{18}N_4O_2$ $[M+Na]^+$ 237.1322)

IR (ATR, cm^{-1}) 3356 (NH secondary amine, br), 2977 (CH aliphatic stretch), 2095 (N_3), 1684 (C=O amide stretch), 1161 (C-O).

(*R*)-4-Azidobutan-2-yl-amine hydrochloride (7)

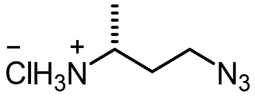

(*R*)-*tert*-butyl(4-azidobutan-2-yl)carbamate (**6**) (0.92 g, 4.3 mmol) was dissolved in anhydrous DCM (6 mL) at room temperature under N_2. A solution of HCl in dioxane (10.70 mL, 42.9 mmol, 4M) was then added cautiously over 5 min. The reaction was then stirred for 1.5 hours, which is when the reaction was deemed complete as determined by TLC. The mixture was then evaporated to give a straw-coloured oil (0.56 g, 3.7 mmol, 88%).

^{1}H NMR (400 MHz, D_2O) δ 3.70 – 3.30 (m, 3H, C*H* & C*H*$_2$N$_3$); 1.88 – 1.65 (m, 2H, C*H*$_2$); 1.20 (d, *J* = 6.7 Hz, 3H, C*H*$_3$).

^{13}C NMR (101 MHz, D_2O) δ 47.30, 45.82, 32.68, 17.34.

HRMS (ESI+) Found 115.0983 $[M]^+$ (Calculated for $C_4H_{11}N_4$ $[M+Na]^+$ 115.0978).

IR (ATR, cm^{-1}) 2881 (NH_3^+, br), 2095 (N_3).

3',5'-Di-*O*-(*tert*-butyldimethylsilyl)-*N*²-(*R*)-(4-azidobutan-2-yl)-*O*⁶-(2-trimethylsilylethyl)-2'-deoxyguanosine (8).

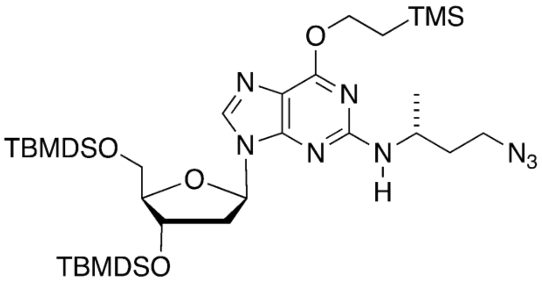

To a room temperature solution of 3',5'-di-*O*-*tert*-butyldimethylsilyl-O^6-(2-trimethylsilylethyl)-2-fluoro-2'-deoxyinosine (**3**) (2.92 g, 4.9 mmol) in dry DMSO (15 mL) under N_2, were added isopropoxytrimethylsilane (17.2 mL, 97.2 mmol) and *N,N*-diisopropylethylamine (6.8 mL, 17.6 mmol). (*R*)-4-Azidobutan-2-yl-amine hydrochloride (**7**) (1.10 g, 7.3 mmol) dissolved in dry DMSO (10 mL) was then added dropwise to the reaction mixture, which was heated for 13 days at 75 ^{0}C. The mixture was cooled to room temperature and water (100 mL) was added. The product was extracted into EtOAc (3 × 100 mL). The organic layers were combined, back-extracted with water (50 mL), dried ($MgSO_4$) and evaporated to give a crude mixture as a golden brown oil (3.35 g, 99%). Crude compound **8** was converted without purification to compound **9,** by hydrogenation as described on page 10.

Data for **8:**

TLC: R_f (20% EtOAc/pet. ether) product = 0.78

^{1}H NMR (400 MHz, DMSO) δ 8.50 (s, 1H, C*H*, H8, starting material); 8.00 (s, 1H, C*H*, H8, product); 6.30 (t, 1H, C*H*, H1', starting material); 6.22 (t, 1H, C*H*, H1', product).

^{19}F NMR (377 MHz, DMSO) δ -50.90 (N=CF starting material), -154.31 ($SiF(CH_3)_3$).

Direct infusion MS (ES+) = 693.

3',5'-di-*O*-(*tert*-butyldimethylsilyl)-*N*²-(*R*)-(4-aminobutan-2-yl)-*O*⁶-(2-trimethylsilylethyl)-2'-deoxyguanosine (9)

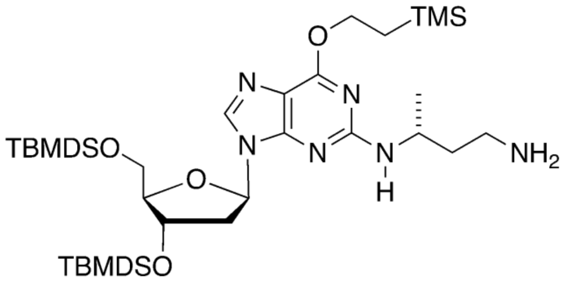

The crude 3',5'-di-*O*-(*tert*-butyldimethylsilyl)-N^2-(*R*)-(4-azidobutan-2-yl)-O^6-(2-trimethylsilylethyl)-2'-deoxyguanosine (**8**) (3.35 g, 4.8 mmol) was dissolved in EtOAc (100 mL) and hydrogenated under a H_2 atmosphere (50 bar) at room temperature over 10%/w Pd/C catalyst in an autoclave. The solvent evaporated to give 3',5'-di-*O*-(*tert*-butyldimethylsilyl)-N^2-(*R*)-(4-aminobutan-2-yl)-O^6-(2-trimethylsilylethyl)-2'-deoxyguanosine as a pale yellow oil (1.39 g, 2.1 mmol, 41% over two steps from **7**).

TLC: R_f (20% MeOH:EtOAc/1% Et_3N) = 0.29

^{1}H NMR (400 MHz, CD_3OD) δ 7.99 (s, 1H, C*H*, H8); 6.34 (t, *J* = 6.6 Hz, 1H, C*H*, H1'); 4.69 – 4.55 (m, 3H, C*H*, H3' & SiC*H₂*); 4.30 – 4.19 (m, 1H, NHC*H*); 4.01 – 3.96 (m, 1H, C*H*, H4'); 3.81 (qd, *J* = 11.1, 4.3 Hz, 2H, CH_2, H5'); 2.92 – 2.74 (m, 3H, NH_2CH_2 & CH_2, H2'); 2.41 (ddd, *J* = 13.3, 6.1, 3.7 Hz, 1H, CH_2, H2'); 1.92 – 1.73 (m, 2H, CHCH_2); 1.30 (d, *J* = 6.5 Hz, 3H, CH_3); 1.27 – 1.20 (t, 2H, OCH_2); 0.96 (s, 9H, C(CH_3)$_3$); 0.92 (s, 9H, C(CH_3)$_3$); 0.17 (s, 6H, Si(CH_3)$_2$); 0.11 (s, 9H, Si(CH_3)$_3$); 0.10 (s, 3H, SiCH_3); 0.08 (s, 3H, SiCH_3).

^{13}C NMR (101 MHz, CD_3OD) δ 162.2, 160.7, 154.7, 138.4, 115.4, 89.3, 85.0, 73.7, 65.6, 64.2, 45.8, 41.0, 39.0, 38.7, 26.5, 26.3, 21.6, 19.3, 18.9, 18.5, -1.3, -4.4, -4.5, -5.2, -5.3.

HRMS (ESI+) Found 667.4229 $[M+H]^+$ (Calculated for $C_{31}H_{62}N_6O_4Si_3$ $[M+H]^+$ 667.4213)

***O*⁶-(2-trimethylsilylethyl)-3',5'-di-*O*-(*tert*-butyldimethylsilyl)-*N*²-(*R*)-(4-trifluoroacetamidobutan-2-yl)-2'-deoxyguanosine (10).**

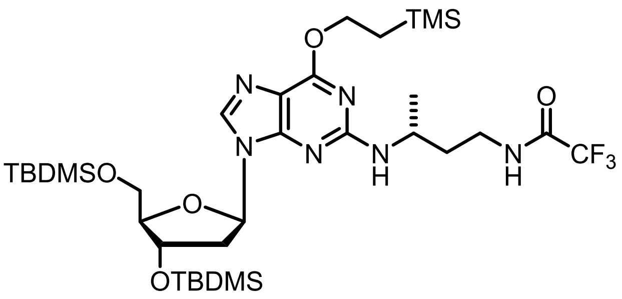

To a solution of 3',5'-Di-*O*-(*tert*-butyldimethylsilyl)-*N*²-(*R*)-(4-aminobutan-2-yl)-*O*⁶-(2-trimethylsilylethyl)-2'-deoxyguanosine (**9**) (1.39 g, 2.1 mmol) in anhydrous DCM (5 mL) stirred at 0°C, dry triethylamine (0.87 mL, 6.3 mmol) was added, followed by the dropwise addition of methyl trifluoroacetate (0.34 mL, 3.3 mmol) over 10 min. The reaction was stirred for a further 30 min, left to warm to room temperature and stirred overnight. The mixture was then evaporated, and the remaining residue was dissolved in DCM (100 mL). The solution was extracted against saturated aq. $NaHCO_3$ (3 × 30 mL) and water (2 × 30 mL). The organic layer was then dried ($MgSO_4$) and evaporated. The crude mixture was purified by flash column chromatography (1% MeOH/EtOAc) to give a white solid (1.3 g, 1.7 mmol, 82%).

TLC: R_f (30% EtOAc/pet. ether) = 0.86

^{1}H NMR (400 MHz, CD_3OD) δ 7.97 (s, 1H, C*H*, H8); 6.33 (t, *J* = 6.6 Hz, 1H, C*H*, H1'); 4.68 – 4.63 (m, 1H, C*H*, H3'); 4.62 – 4.56 (m, 2H, $CH_2Si(CH_3)_3$); 4.23 – 4.12 (m, 1H, NHC*H*); 3.97 (dd, *J* = 7.4, 4.1 Hz, 1H, C*H*, H4'); 3.82 (ddd, *J* = 15.0, 11.1, 4.4 Hz, 2H, CH_2, H5'); 3.49 – 3.35 (m, 2H, $NHCH_2$); 2.92 – 2.72 (m, 1H, C*H*, H2'); 2.37 (ddd, *J* = 13.2, 6.1, 3.5 Hz, 1H, C*H*, H2'); 1.92 – 1.80 (m, 2H, $CHCH_2$); 1.28 (d, *J* = 6.6 Hz, 3H, CH_3); 1.23 (t, 2H, OCH_2); 0.95 (s, 9H, $C(CH_3)_3$); 0.91 (s, 9H, $C(CH_3)_3$); 0.16 (s, 6H, $Si(CH_3)_2$); 0.10 (s, 9H, $Si(CH_3)_3$); 0.09 (s, 3H, $SiCH_3$); 0.08 (s, 3H, $SiCH_3$).

^{13}C NMR (101 MHz, CD_3OD) δ 160.7, 159.1, 158.0, 157.6, 157.2, 156.9, 153.3, 137.4, 120.4, 117.6, 114.7, 114.0, 111.9, 87.8, 84.0, 72.4, 64.1, 62.9, 44.8, 39.7, 37.0, 35.2, 25.1, 24.9, 20.0, 17.9, 17.5, 17.1, -2.7, -5.8, -5.9, -6.6, -6.6.

^{19}F NMR (377 MHz; $CDCl_3$) -77.3 (1F, CF_3)

Acc. Mass (ESI+) Found 763.4048 $[M+H]^+$ (Calculated for $C_{33}H_{61}F_3N_6O_5Si_3$ $[M+H]^+$ 763.4036).

N^2-(*R*)-(4-trifluoroacetamidobutan-2-yl)-O^6-(2-trimethylsilylethyl)-2'-deoxyguanosine (11)

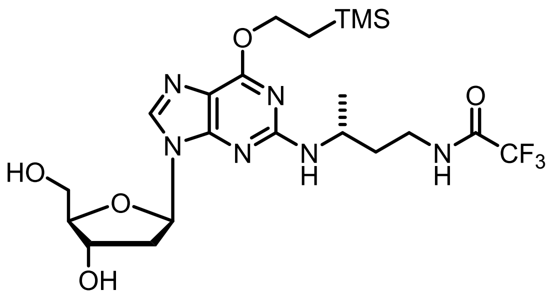

To a solution of O^6-(2-trimethylsilylethyl)-3',5'-di-*O*-(*tert*-butyldimethylsilyl)-N^2-(*R*)-(4-trifluoroacetamidobutan-2-yl)-2'-deoxyguanosine (**10**) (1.00 g, 1.3 mmol) in anhydrous THF (10 mL), triethylamine trihydrofluoride (0.7 mL, 4.3 mmol) was added dropwise. The mixture was stirred at room temperature overnight then evaporated. The crude mixture was dissolved in MeOH (10 mL), dried onto a short silica plug and eluted with EtOAc to afford N^2-(*R*)-(4-trifluoroacetamidobutan-2-yl)-O^6-(2-trimethylsilylethyl)-2'-deoxyguanosine as a white crystalline solid (0.55 g, 1.0 mmol, 79%).

TLC: R_f (20% MeOH/EtOAc) product = 0.69

^{1}H NMR (400 MHz, CD_3OD) δ 8.02 (s, 1H, C*H*, H8); 6.34 (t, *J* = 6.9 Hz, 1H, C*H*, H1'); 4.74 – 4.52 (m, *J* = 13.0, 10.9, 4.6 Hz, 3H, C*H*, H3' & SiCH_2); 4.24 – 4.13 (m, 1H, NHC*H*); 4.03 (d, *J* = 2.9 Hz, 1H, C*H*, H4'); 3.79 (ddd, *J* = 33.4, 12.0, 3.7 Hz, 2H, CH_2, H5'); 3.41 (t, *J* = 7.2 Hz, 2H, NHCH_2); 2.87 (dt, *J* = 13.8, 6.9 Hz, 1H, C*H*, H2'); 2.37 (ddd, *J* = 13.2, 5.9, 2.8 Hz, 1H, C*H*, H2'); 1.90 – 1.81 (m, 2H, CHCH_2); 1.28 (d, *J* = 6.6 Hz, 3H, CH_3); 1.24 (t, *J* = 8.4 Hz, 2H, OCH_2); 0.11 (s, 9H, Si(CH_3)$_3$).

^{13}C NMR (101 MHz, CD_3OD) δ 160.8, 158.9, 158.0, 157.6, 157.3, 156.9, 153.0, 138.2, 120.4, 117.6, 114.7, 114.3, 111.9, 87.9, 84.9, 71.6, 64.2, 62.3, 44.9, 39.4, 37.0, 35.1, 20.0, 17.1, -2.7.

^{19}F NMR (377 MHz, CD_3OD) δ 77.3 (1F, CF_3)

HRMS (ESI+) Found 535.2307 $[M+H]^+$ (Calculated for $C_{21}H_{33}F_3N_6O_5Si$ $[M+H]^+$ 535.2307).

5'-*O*-(4,4'-Dimethoxytrityl)-*N*²-(*R*)-(4-trifluoroacetamidobutan-2-yl)-*O*⁶-(2-trimethylsilylethyl)-2'-deoxyguanosine (12)

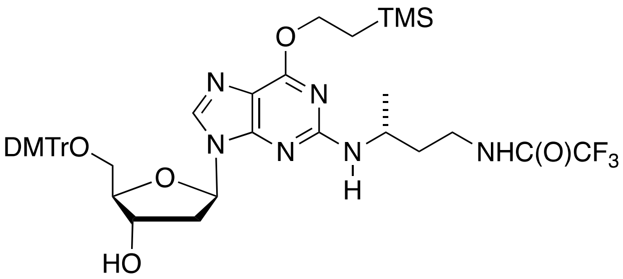

N^2-(*R*)-(4-trifluoroacetamidobutan-2-yl)-*O*^{6}-(2-trimethylsilylethyl)-2'-deoxyguanosine (**11**) (0.53 g, 1.0 mmol) was dissolved in anhydrous pyridine (7 mL) room temperature under argon. To this solution, triethylamine (0.18 mL, 1.3 mmol) and 4-(dimethylamino)pyridine (0.006 g, 0.05 mmol) were added. 4,4'-Dimethoxytrityl chloride (0.4 g, 1.2 mmol) was then added portion-wise over 30 min to the mixture and was left to stir at room temperature overnight. The reaction progression was monitored by TLC. When deemed complete, MeOH (5 mL) was added, and the solvents were removed under vacuum. The crude mixture was re-dissolved in DCM (100 mL), extracted against saturated $NaHCO_3$ (3 × 50 mL) and water (2 × 50 mL), and then dried ($MgSO_4$). Solvents were evaporated and the crude residue was purified by flash column chromatography (65% EtOAc/pet. ether/1% Et_3N) to give 5'-*O*-(4,4'-dimethoxytrityl)-*N*^{2}-(*R*)-(4-trifluoroacetamidobutan-2-yl)-*O*^{6}-(2-trimethylsilylethyl)-2'-deoxyguanosine as a white crystalline solid (0.43 g, 0.5 mmol, 52%).

TLC: R_f (65% EtAOc/Pet ether/1% Et_3N) product = 0.44

^{1}H NMR (400 MHz, $CDCl_3$) δ 7.68 (s, 1H, C*H*, H8); 7.41 (d, *J* = 7 Hz, 2H, Ar*H*); 7.25 (ddd, *J* = 21.1, 13, 8 Hz, 7H, Ar*H*); 6.80 (d, *J* = 9 Hz, 4H, Ar*H*); 6.30 (t, *J* = 6.5 Hz, 1H, C*H*, H1'); 4.70 – 4.53 (m, 3H, C*H*, H3' & C*H*$_2$*Si*); 4.18 – 4.04 (m, 2H, C*H* & C*H*, H4'); 3.79 (s, 6H, (C*H*$_3$)$_2$); 3.59 – 3.47 (m, *J* = 8 Hz, 1H, NHC*H*$_2$); 3.33 (ddd, *J* = 25, 10, 5 Hz, 2H, C*H*$_2$, H5'); 3.18 – 3.05 (m, *J* = 4.5 Hz, 1H, NHC*H*$_2$); 2.89 – 2.77 (m, 1H, C*H*$_2$, H2'); 2.53 – 2.43 (m, 1H, C*H*$_2$, H2'); 1.91 – 1.78 (m, *J* = 15, 6 Hz, 1H, C*H*$_2$); 1.64 – 1.52 (m, *J* = 4, 1 Hz, 1H, C*H*$_2$); 1.27 – 1.19 (m, *J* = 12, 5 Hz, 5H, OC*H*$_2$ & C*H*$_3$); 0.10 (s, 9H, (C*H*$_3$)$_3$).

^{13}C NMR (101 MHz, $CDCl_3$) δ 161.3, 158.9, 158.6, 157.1, 144.6, 136.8, 135.7, 130.0, 128.1, 127.9, 127.0, 117.4, 115.6, 114.5, 113.2, 86.5, 85.5, 83.1, 72.6, 65.1, 64.0, 55.2, 44.5, 39.7, 37.1, 36.8, 21.8, 17.7, -1.4.

^{19}F NMR (377 MHz, $CDCl_3$) δ -75.7 (1F, CF_3)

HRMS (ESI+) Found 837.3619 $[M+H]^+$ (Calculated for $C_{42}H_{51}F_3N_6O_7Si$ $[M+H]^+$ 837.3613).

5'-*O*-(4,4'-dimethoxytrityl)-*N*[2]-(*R*)-(4-trifluoroacetamidobutan-2-yl)-*O*[6]-(2-trimethylsilylethyl)-2'-deoxyguanosine-3'-*O*-(2-cyanoethyl)-*N,N'*-diisopropylphosphoramidite (13)

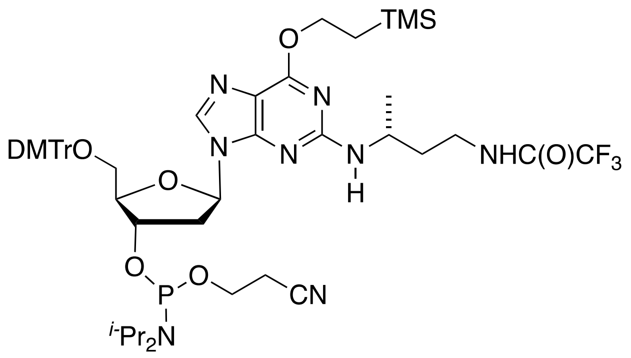

5'-*O*-(4,4'-dimethoxytrityl)-*N*[2]-(*R*)-(4-trifluoroacetamidobutan-2-yl)-*O*[6]-(2-trimethylsilylethyl)-2'-deoxyguanosine (**12**) (0.23 g, 0.3 mmol) was dried under vacuum then dissolved in anhydrous DCM (1.6 mL). 2-Cyanoethyl *N,N,N',N'*-tetraisopropylphosphorodiamidite (0.13 mL, 0.4 mmol) was then added dropwise, and the solution was stirred at room temperature under argon. 5-(Ethylthio)-*1H*-tetrazole (0.04 g, 0.3 mmol) was dried under vacuum, dissolved in anhydrous acetonitrile (1.1 mL) and added dropwise to the reaction over 30 min. The mixture was left stirring at room temperature and under argon for 2 h. The solvent was then evaporated, and the residue was dissolved in EtOAc (50 mL). The organic mixture was extracted against $NaHCO_3$ (3 × 30 mL) and water (2 × 30 mL). The organic layer was then dried ($MgSO_4$). After removal of solvent by evaporation, the crude product was purified by flash column chromatography (30 % EtOAc/pet.ether/10% Et_3N) to give a white solid (0.25 g, 0.3 mmol, 89%).

TLC: R_f (30 % EtOAc/pet.ether/10% Et_3N) product: 0.64, product: 0.46

^{1}H NMR (400 MHz, CD_3CN) δ 7.73 (dd, *J* = 5.5, 1.7 Hz, 1H, C*H*, H8); 7.60 (broad s, 1H, N*H*COCF_3); 7.43 – 7.35 (m, 2H, Ar*H*); 7.25 (dt, *J* = 10.8, 8.5 Hz, 7H, Ar*H*); 6.79 (ddd, *J* = 18.4, 10.3, 6.6 Hz, 4H, Ar*H*); 6.28 (td, *J* = 6.4, 3.1 Hz, 1H, C*H*, H1'); 5.35 – 5.18 (m, 1H, N*H*); 4.83 (tdd, *J* = 10.9, 6.7, 4.5 Hz, 1H, C*H*, H3'); 4.65 – 4.53 (m, 2H, CH_2Si); 4.20 – 3.79 (m, 4H, C*H*NH & C*H*, H4' & CH_2); 3.77 (t, J = 2.0 Hz, 6H, $(CH_3)_2$) 3.71 – 3.55 (m, 3H, CH_2OP & CH_2NH); 3.38 – 3.23 (m, 4H, $(NCH)_4$ & CH_2, H5'); 3.06 (t, *J* = 12.0, 6.2 Hz, 1H, CH_2NH); 2.66 (dt, *J* = 5.9 Hz, 2H, CH_2, H2'); 2.51 (dd, *J* = 14.2, 7.0 Hz, 2H, CH_2CN); 1.84 – 1.68 (m, 2H, CH_2); 1.26 – 1.12 (m, 14H, $(iPr)_2$N); 1.07 (d, *J* = 6.8 Hz, 3H, CH_3); 1.00 (t, *J* = 7.1 Hz, 2H, CH_2O); 0.11 (s, 9H, Si$(CH_3)_3$).

^{13}**C NMR** (101 MHz, CD_3CN) δ 160.88, 158.69, 158.64, 158.59, 158.58, 156.83, 156.47, 145.16, 145.13, 137.94, 137.85, 135.91, 135.83, 135.78, 130.05, 130.01, 129.91, 129.88, 128.01, 127.93, 127.77, 127.75, 126.80, 126.76, 112.99, 85.98, 85.93, 85.38, 85.34, 85.09, 85.03, 83.86, 83.79, 74.05, 73.86, 73.54, 64.21, 63.98, 63.86, 58.50, 58.46, 58.31, 58.28, 54.87, 46.06, 44.90, 43.12, 43.00, 37.84, 37.06, 35.34, 23.96, 23.92, 23.89, 23.88, 23.85, 23.81, 20.39, 20.35, 20.09, 20.01, 20.01, 19.93, 17.11, 11.10, -2.34.

^{19}**F NMR** (377 MHz, CD_3CN) δ -76.52 (1F, CF_3); -75.74 (1F, CF_3)

^{31}**P NMR** (162 MHz, CD_3CN) δ 148.40, 148.13

HRMS (ESI+) Found 1037.4675 $[M+H]^+$ (Calculated for $C_{51}H_{68}F_3N_8O_8PSi$ $[M+H]^+$ 1037.4692).

Oligodeoxyribonucleotide (ODN) methods

Oligodeoxyribonucleotide (ODN) synthesis and purification

Except for dG_{COMP}-ODN, which was purchased without purification from ATDBio Ltd, oligodeoxyribonucleotides (ODNs) were synthesised on an Applied Biosystems 394 DNA/ RNA automated synthesiser on a 1 μmol scale. Solid Chemical Phosphorylation Reagent (^{solid}CPR™) and base labile phosphoramidites (acetyl-dC, isopropyl-phenoxyacetyl-dG, phenoxyacetyl-dA, dT amidites) were purchased from Link Biosearch Technologies. All oligonucleotides were synthesised with the final dimethoxytrityl group (DMT) removed (DMT-OFF). The N^2-((R)-4-trifluoroacetamidobutan-2-yl)-dG phosphoramidite (**13**) was used at 100 mg/mL and was coupled for 6 minutes. The ^{solid}CPR™ reagent was used according to the manufacturers protocol in the final step of the synthesis with a 6-minute coupling time followed by a final detritylation step (DMT-OFF). O^6-(trimethylsilylethyl)-2-fluoro-2'-deoxyinosine (dI_{2F}) phosphoramidite was purchased from Chem Genes.

ODNs synthesised:

dG_{AB}-ODN: 5'-pGCACGAAAGAAGAGC(dG_{AB})GAAG3'

dG-ODN: 5'-pGCACGAAAGAAGAGC(dG)GAAG3'

dI_{2F}-ODN: 5'-pCCCTCTTCC(dI_{2F})CTCTTCTTTC3'

dG_{COMP}-ODN: 5'-pCCCTCTTCCGCTCTTCTTTC3'

In house synthesized ODNs were treated with 5 mL of 10% diethylamine (DEA) in MeCN using a Harvard Apparatus 11 Plus syringe pump at 0.5 ml/min to remove the cyanoethyl protecting groups over 5 minutes. The columns were then washed with MeCN and ODNs were cleaved from the support and deprotected using concentrated aqueous ammonia at room temperature for 6 hrs. Column cleavage and deprotection of the dI_{2F}-ODN was carried out using 0.1 M aq NaOH at room temperature for 20 h followed by neutralisation with 0.1 M AcOH. The O^6-TMSethyl protecting group was then removed by a subsequent treatment with 5% aqueous acetic acid. All ODNs were desalted using NAP-10 (GE Healthcare) gel filtration columns. Crude ODNs were then purified by RP-IP-HPLC using a 4.6 x 250 mm Gemini C18 column (Phenomenex) at 55 °C on a WAVE HPLC system (ADSBio) with UV detection at 260 nm using D-6000 HSM software (Hitachi) and the following: Buffer A (100 mM TEAAc pH 7.0, 0.1% MeCN) and buffer B (100 mM TEAAc pH 7.0, 50% MeCN).

ODN Mass Spectrometry

ODN samples were analysed by RP-IP-HPLC on a Vanquish binary gradient UHPLC system (Thermo Fisher Scientific) using a DNAPac RP (100 mm × 2.1 mm I.D. Thermo Scientific). The mobile phases were as follows: LC buffer A: 0.2% Triethylamine (TEA), 50 mM 1,1,1,3,3,3,-Hexafluoro-2-propanol (HFIP); LC buffer B: 0.2% Triethylamine (TEA), 50 mM HFIP, 20% v/v MeCN. Starting with 2% buffer B, the oligos were separated using a linear gradient up to 25% B in 40 mins at 60 °C and at a flow rate of 200 µl min^{-1}. Mass Spectrometry analysis was performed using an Orbitrap Exploris 240 LC MS instrument (ThermoFisher Scientific). Data acquisition was performed using DDA in full scan negative mode (scanning 450 to 3000 m/z) with an MS1 resolution of 120,000 and a normalized Automatic Gain Control (AGC) target of 200%. MS1 ions were selected for higher energy collisional dissociation (HCD). MS2 resolution was set at 30,000 with the AGC target at 50% with an isolation window of 4 m/z, a scan range of 150-2000 m/z and a normalized stepped collision energy 15, 18, 21. Data analysis was performed in Freestyle (ThermoFisher Scientific) using Xtract Deconvolution.

ODN Crosslinking Reaction

dI_{2F}-ODN (2.7 nmol) was combined with either dG-ODN (2.4 nmol) or dG_{AB}-ODN (2.4 nmol) in 50 mM sodium borate pH 9.0 buffer (total volume 60 µL). The mixtures were immediately heated at 95 °C for one minute and allowed to cool to room temperature for 10 minutes. At this point, a 6 µL aliquot from each mixture was removed and quenched in 54 µL of 111 mM TEAA pH 6.5 (this is considered the 'zero' time point). The remaining volume was incubated at room temperature for the indicated time, at which point a 6 µL aliquot from each mixture was removed and quenched as above. Quenched reactions were stored at -20 °C until analysis by HPLC.

HPLC analysis and quantitation of crosslinking reaction

Reaction mixtures were analysed at 55 °C by denaturing HPLC using a 4.6 × 150 mm AdvanceBio Oligo column (Agilent) and a WAVE HPLC system (ADS Bio) with D-6000 HSM software (Hitachi). A 25 µL aliquot of quenched reaction mixture (~100 pmol duplex) was analysed. Separation of individual strands and cross-linked duplex was achieved using buffer A (100 mM TEAA pH 7.0, 0.1% MeCN) and buffer B (100 mM TEAA pH 7.0, 25% MeCN) and the following gradient run at 0.9 mL/min: 20% B, 0-1 min; 20-80% B, 1-21 min; 80%-100% B, 21-22 min; 100 % B, 22-24 min; 100-20% B, 24-25 min; 20% B, 25-28 min. Chromatograms were processed by subtracting a baseline generated by injecting a blank (25 µL injection of 100 mM TEAA pH 6.5, 0.1% MeCN containing 5 mM sodium borate). Peaks from individual strands were identified using standards injected in the same solution as the blank.

To quantify, the peak areas were integrated using the associated software. The peak areas corresponding to the dG_{AB}-ODN, dG-ODN, dI_{2F}-ODN, and AA_{RED}ICL were normalized by dividing the peak area by the associated extinction coefficient (dG_{AB}-ODN and dG-ODN: 215,800 M^{-1} cm^{-1}; dI_{2F}-ODN: 154,600 M^{-1} cm^{-1}; and AA_{RED}ICL duplex: 320,026 M^{-1} cm^{-1} (adjusted for duplex hypochromicity using equations 1 and 2 (https://atdbio.com/nucleic-acids-book/Ultraviolet-absorbance-of-oligonucleotides#Hypochromicity-and-double-stranded-DNA). Because dG_{AB}-ODN was the limiting strand in the duplex crosslinking reaction, the percentages of dG_{AB}-ODN, dI_{2F}-ODN and AA_{RED}ICL duplex were calculated according to equations 3, 4 and 5, respectively. Plots of chromatograms and quantitation were generated in Graph Prism 9.3.1.

$$h = (0.059 \times f_{GC}) + (0.287 \times f_{AT}) \qquad \text{Equation 1}$$

$$E_{ds,260} = (E_{ss,260} + E_{reverse\ complement,260}) \times (1 - h) \qquad \text{Equation 2}$$

$$\%dG_{AB} - ODN = \frac{Area_{dG_{AB} - ODN}}{(Area_{dG_{AB} - ODN} + Area_{AA_{RED}ICL})} \times 100 \qquad \text{Equation 3}$$

$$\%dI_{2F} - ODN = \frac{Area_{dI_{2F} - ODN}}{(Area_{dI_{2F} - ODN} + Area_{AA_{RED}ICL})} \times 100 \qquad \text{Equation 4}$$

$$\%AA_{RED}ICL = \frac{Area_{AA_{RED}ICL}}{(Area_{dG_{AB} - ODN} + Area_{AA_{RED}ICL})} \times 100 \qquad \text{Equation 5}$$

Polyacrylamide gel electrophoresis (PAGE)

Denaturing PAGE was achieved using a 15% (19:1) acrylamide gel containing 7M urea and 1×TBE (89 mM Tris, 89 mM Boric acid, 1 mM EDTA). Gel solution (35 mL) was prepared from the appropriate stock solutions and de-gassed under vacuum for approximately five minutes. To initiate polymerization, 10% aqueous ammonium persulfate (g/v) (10% APS; 280 μl) and tetramethylethylenediamine (TEMED; 14 μL) were added to the solution and stirred. The gel was immediately poured into a pre-assembled 16.5 × 22 × 0.1 cm slab gel cast (C.B.S. Scientific) and a 0.1 cm, 15-well comb was inserted to create the wells. The gel was laid horizontally for at least one hour to allow the gel solution to completely polymerize. Once set, the gel was pre-rerun with 1×TBE running buffer (AppliChem GmbH) for 1 hour at 35 W (~2.1 W/cm). ODN samples were diluted with water and 2× PAGE loading dye (95% formamide, 0.02% bromophenol blue, 0.01% xylene cyanol, 0.1 mM EDTA, 0.02 % SDS) to a final ODN concentration of 10 ng/μl ODN in 1× PAGE loading dye. Wells were loaded with 10 μL of the

indicated ODN sample or 1× PAGE loading dye for empty wells, and the gel was electrophoresed at constant power (35 W) using a 1×TBE running buffer. When the bromophenol blue standard approached an Rf ~3/4 the length of the gel, it was removed from the casting plates and placed into 1× SYBR™ gold stain in 1×TBE for 15 minutes. The bands were then visualized with a ChemiDoc MP Imaging System and Image Lab software (Bio-Rad Laboratories, Inc) according to the manufacturer's protocol.

HPLC Purification of $AA_{RED}ICL$

$AA_{RED}ICL$ was purified from starting materials using a 7.8 × 10 mm OligoSep Prep HC cartridge (ADS Biotec Inc.) and a WAVE HPLC system equipped with an FCW-180 fraction collector and WAVEMAKER 4.1 software (ADS Biotech Inc.). Separation of individual strands and cross-linked duplex was achieved using buffer A (100 mM TEAA pH 7.0, 0.1% MeCN) and buffer B (100 mM TEAA pH 7.0, 25% MeCN) and the following gradient run at 1.5 mL/min and 80 °C: 25% B, 0-1 min; 25-65% B, 1-21 min; 80%-100% B, 21-22 min; 100 % B, 22-24 min; 100-20% B, 24-25 min; 25% B, 25-28 min. The starting materials and $AA_{RED}ICL$ product were fractionated and pooled. The MeCN was removed by rotoevaporation, and the remaining solvent was removed by lyophilization.

UV Thermal Melting

DNA melting was performed using a Cary 3500 UV-Vis Multicell Peltier system (Agilent) with the associated Cary UV Workstation software (v1.3.4). Samples were melted in reduced-volume (500 μL) 10 mm pathlength cuvettes at 1.5 μM heteroduplex concentration (3 μM [strand]) in 10 mM sodium phosphate buffer pH 7.0, 100 mM NaCl and 50 μM EDTA. Once samples were prepared, they were placed in the cuvette, layered with 100 μL of mineral oil, and stoppered. Using the Cary 3500 instrument, the samples heated, annealed, and melted according to the parameters listed in table S1. The first derivative curves, which were derived from the smoothed data generated using the Savitzky-Golay technique (filter 11, interval 1), were used to estimate the melting temperature (t_m).

Table S1. DNA thermal melting instrument parameters.

Stage	Collect Data?	Start (°C)	Data Interval (°C/min)	Ramp Rate (°C/min)	End (°C)	Hold (min)
**1**	No	25.0	NA‡	10.0	90.0	5.00
**2**	No	90.0	NA	2.0	25.0	5.00
**3**	Yes	25.0	1.0	1.0	95.0	0.50
**4**	Yes	95.0	1.0	1.0	25.0	0.00

‡NA-Not applicable because data is not being collected.

DNA replication assays methods

Xenopus laevis egg extracts

Egg extracts were prepared using eggs from adult *X. laevis* female frogs (aged >2 years; Nasco, catalog no. LM00535), and de-membranated sperm chromatin was prepared from the testes of adult *X. laevis* male frogs (purchased from the European Xenopus Resource Centre). All animal procedures and experiments were performed in accordance with national animal welfare laws and were reviewed by the Animal Ethics Committee of the Royal Netherlands Academy of Arts and Sciences (KNAW). All animal experiments were conducted under a project license granted by the Central Committee Animal Experimentation (CCD) of the Dutch government and approved by the Hubrecht Institute Animal Welfare Body (IvD), with project license number AVD80100201711044.

Xenopus egg extracts and DNA replication/repair assays

X. laevis egg production, preparation of *Xenopus* HSS, demembranated sperm chromatin, NPE, and DNA replication were performed as previously described.[2] Duplex oligonucleotides containing site-specific interstrand crosslinks were ligated into a plasmid backbone to generate crosslinked plasmids (pICL-Pt and PICL-AA_{RED}) as described.[3] DNA replication and NotI repair assays were performed as previously described.[4]

Supporting Figures

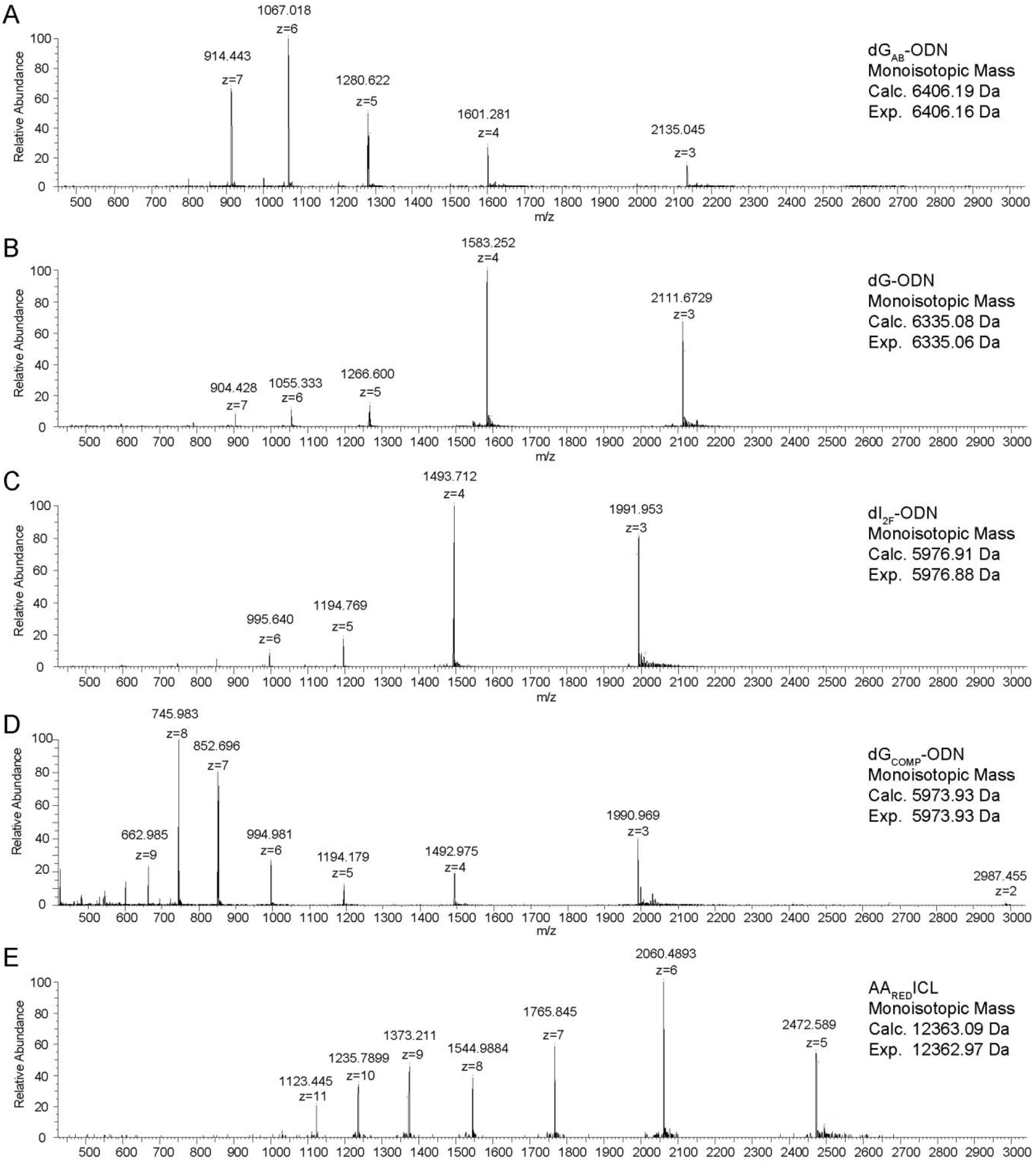

Figure S1 LC-MS results for **A** dG_{AB}-ODN, **B** dG-ODN, **C** dI_{2F}-ODN, **D** dGcomp-ODN and E AA_{RED}ICL with the calculated (Calc.) and experimental (Exp.) monoisotopic masses shown. Detected ions are labelled with their average mass-to-charge ratio (m/z) and the charge state (z) in each MS1 spectrum.

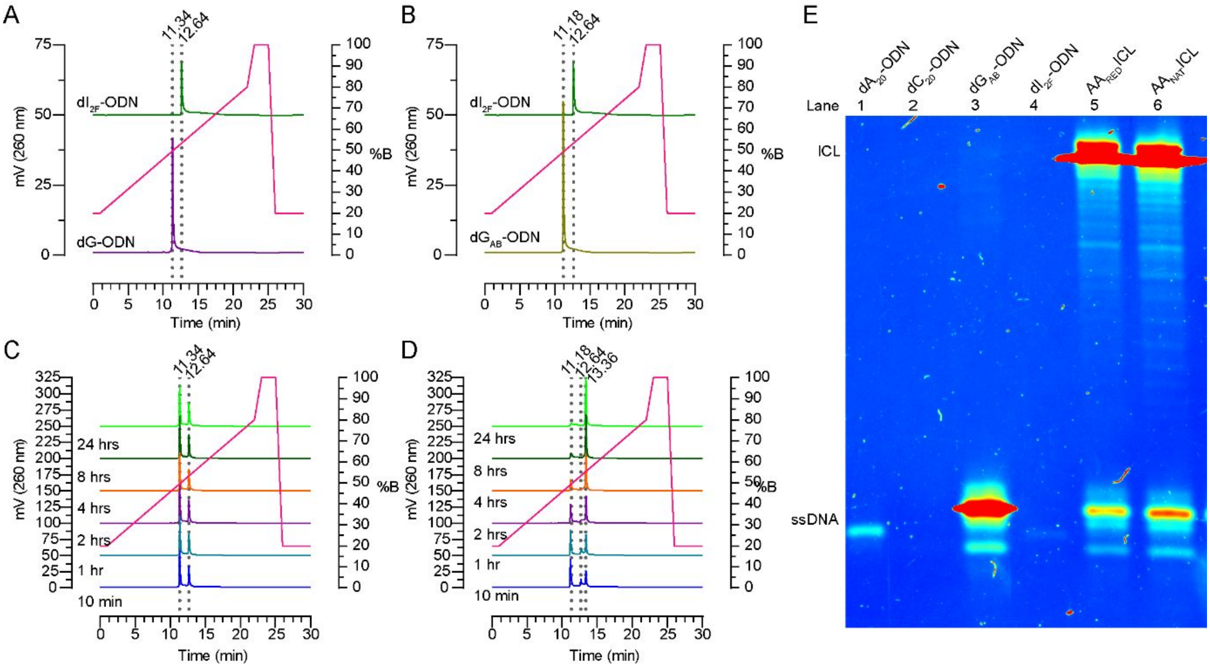

Figure S2 Analysis of control and ICL reactions by RP-IP-HPLC and denaturing PAGE. **A,B** To facilitate peak identification, individual chromatograms of the **A** dI_{2F}-ODN (green) and dG-ODN (purple) and **B** dI_{2F}-ODN (green) and dG_{AB}-ODN (olive green) are shown. **A,B** For clarity, the individual chromatograms are offset by 50 mV using the Graphpad Prism 9.3.1 software. The gradient that was used to separate the strands is shown as a magenta line and is plotted relative to the right Y-axis. The gradient is plotted to account for the dead volume (~1 min) between the pump and the detector. Retention times for the strands are shown above, and a dotted line is included to guide the eye. Chromatograms of reaction progress as a function of quench time for **C** a control reaction containing dG-ODN and dI_{2F}-ODN and **D** an ICL reaction containing dG_{AB}-ODN and dI_{2F}-ODN. **C,D** These results are displayed as described in figure 1A,B; however, the full chromatogram is shown. **E** Same 15% (19:1) denaturing PAGE stained with SYBR™ gold shown in Figure 1D, but purposefully overexposed to highlight faint bands. Lane numbers and sample identities are labelled. Poor binding of cyanine dyes like SYBR™ gold to homopyrimidines or ssDNA composed of just A, C or T (sequence preference G>A>T>C) has been discussed in Figure 1D.[5] Overexposure of the gel does reveal some staining of dA_{20} (lane 1) as well as the dI_{2F}-ODN (lane 4). Similar differential staining trends have also been observed for ethidium bromide and silver staining methodologies;[6] therefore, these methods are not viable alternatives to using SYBR™ gold.

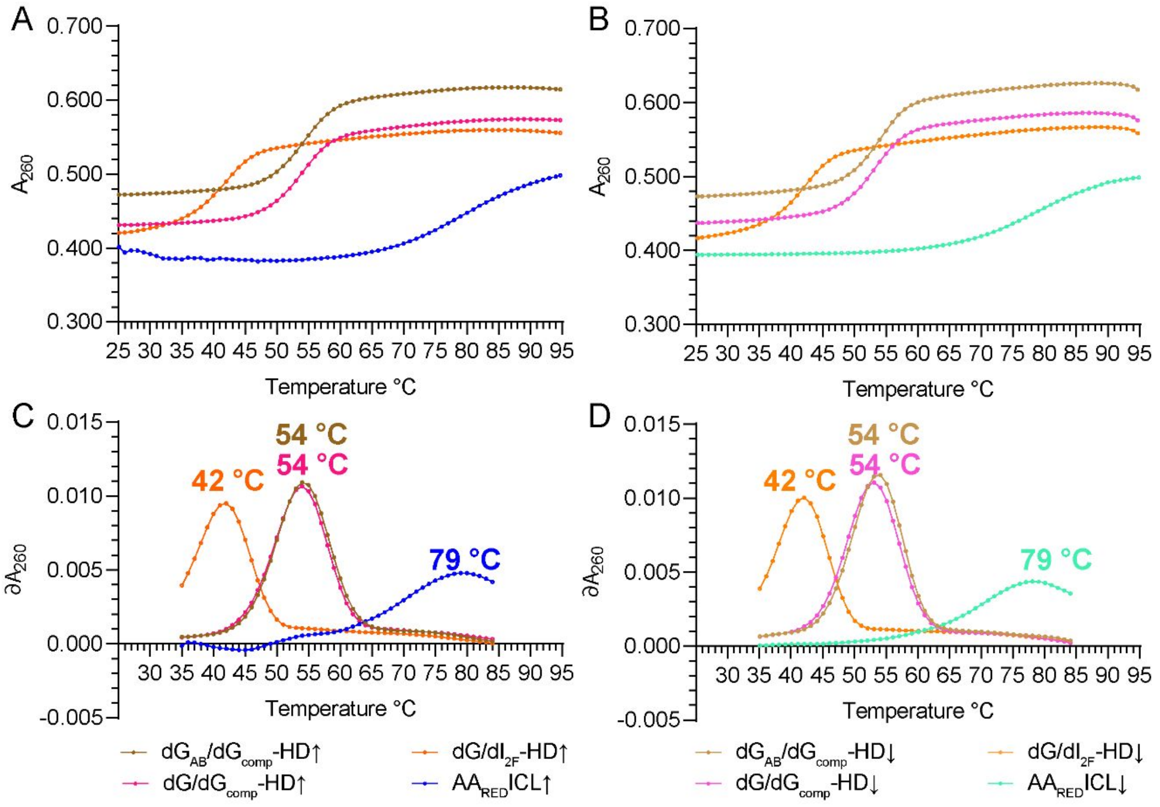

Figure S3 Summary of UV thermal melting data of various heteroduplexes (HDs) and AA_{RED}ICL**.** The data from Figure 2 is divided into **A** forward (25 °C to 95 °C) and **B** reverse (95 °C to 25 °C) melts for clarity. The intermolecular HDs (dG/dG_{comp}, dG_{AB}/dG_{comp} and dG/dI_{2F}) melt at a considerably lower temperature than the corresponding intramolecular AA_{RED}ICL, which is consistent with a covalent link. The transition of the AA_{REDI}CL is considerably broader than the HD transitions suggesting less two-state melting character for the cross-linked species. **C,D** The first derivatives (∂A_{260nm}) of the data in panels **A** and **B**, which were generated after smoothing of the data using the Savitsky-Golay technique (filter 11, interval 1), are plotted for the **C** forward and **D** reverse melts. The melting temperatures (T_m) are estimates derived from the maxima of the first derivative curves. Plots were generated using Graphpad Prism 9.3.1 software.

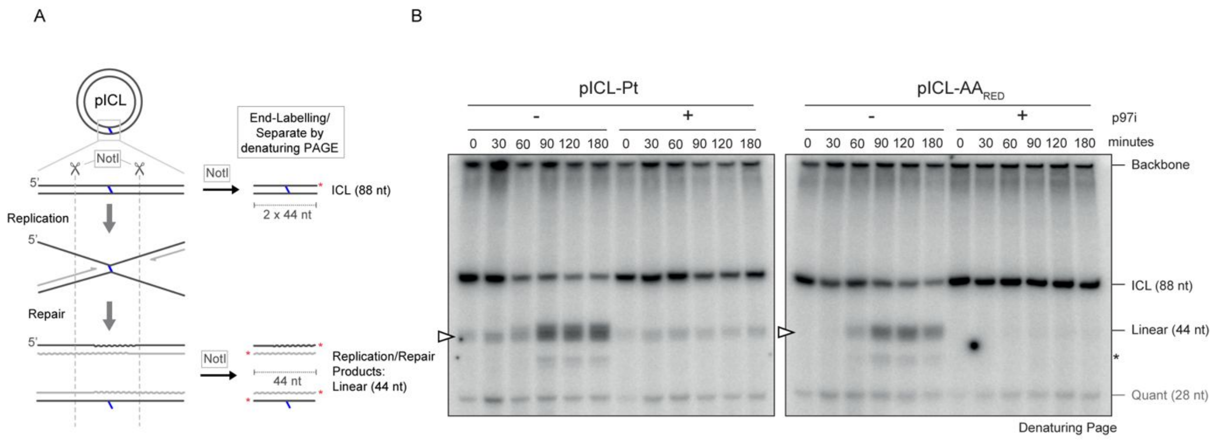

Figure S4 A Schematic representation of the NotI-based ICL repair assay. Wavy lines indicate the part of the strand that is synthesized during repair. In this assay, RRI are digested using NotI, radioactively labelled at the 3′ end and separated by denaturing PAGE. Before DNA replication (t = 0), only the 88 nucleotide (nt) (2 × 44 nt, crosslinked) fragment and the unresolved vector backbone are observed. During ICL repair, the 44-nt fragment accumulates, which is quantified to determine the percentage of repair. **B** After replication of the plasmids containing cisplatin- or reduced-acetaldehyde-induced ICLs (pICL-Pt and pICL-AA_{RED}, respectively) in Xenopus egg extract, the RRI are isolated, digested by NotI, and resolved by denaturing PAGE. Accumulation of the 44-nt product (white arrow) indicates repair. Some 44 nt product is present at t=0; this arises from contaminating non-crosslinked plasmid present in pICL-Pt and pICL-AA_{RED}, albeit far less in the latter. The asterisk marks a product that is probably generated from end-joining activity in some extracts.

References

1. Decorte, B. L.; Tsarouhtsis, D.; Kuchimanchi, S.; Cooper, M. D.; Horton, P.; Harris, C. M.; Harris, T. M. Improved Strategies for Postoligomerization Synthesis of Oligodeoxynucleotides Bearing Structurally Defined Adducts at the N2 Position of Deoxyguanosine. *Chem Res Toxicol* **1996**, *9*, 630–637.

2. Sparks, J.; Walter, J. C. Extracts for Analysis of DNA Replication in a Nucleus-Free System. *Cold Spring Harb Protoc* **2019**, pdb.prot097154.

3. Enoiu, M.; Jiricny, J.; Schärer, O. D. Repair of Cisplatin-Induced DNA Interstrand Crosslinks by a Replication-Independent Pathway Involving Transcription-Coupled Repair and Translesion Synthesis. *Nucleic Acids Res* **2012**, 40, 8953–8964.

4. Hodskinson, M. R.; Bolner, A.; Sato, K.; Kamimae-Lanning, A. N.; Rooijers, K.; Witte, M.; Mahesh, M.; Silhan, J.; Petek, M.; Williams, D. M.; Kind, J.; Chin, J. W.; Patel, K. J.; Knipscheer, P. Alcohol-Derived DNA Crosslinks Are Repaired by Two Distinct Mechanisms. *Nature* **2020**, 579, 603–608.

5. Han, X.; Wang, E.; Cui, Y.; Lin, Y.; Chen, H.; An, R.; Liang, X.; Komiyama, M. The Staining Efficiency of Cyanine Dyes for Single-Stranded DNA is Enormously Dependent on Nucleotide composition. *Electrophoresis* **2019**, 40, 1708-1714.

6. Tang, W.; Zhou, H.; Li, W. Silver and Cyanine Staining of Oligonucleotides in Polyacrylamide Gel. *PLoS ONE* **2015** 10(12):e0144422.

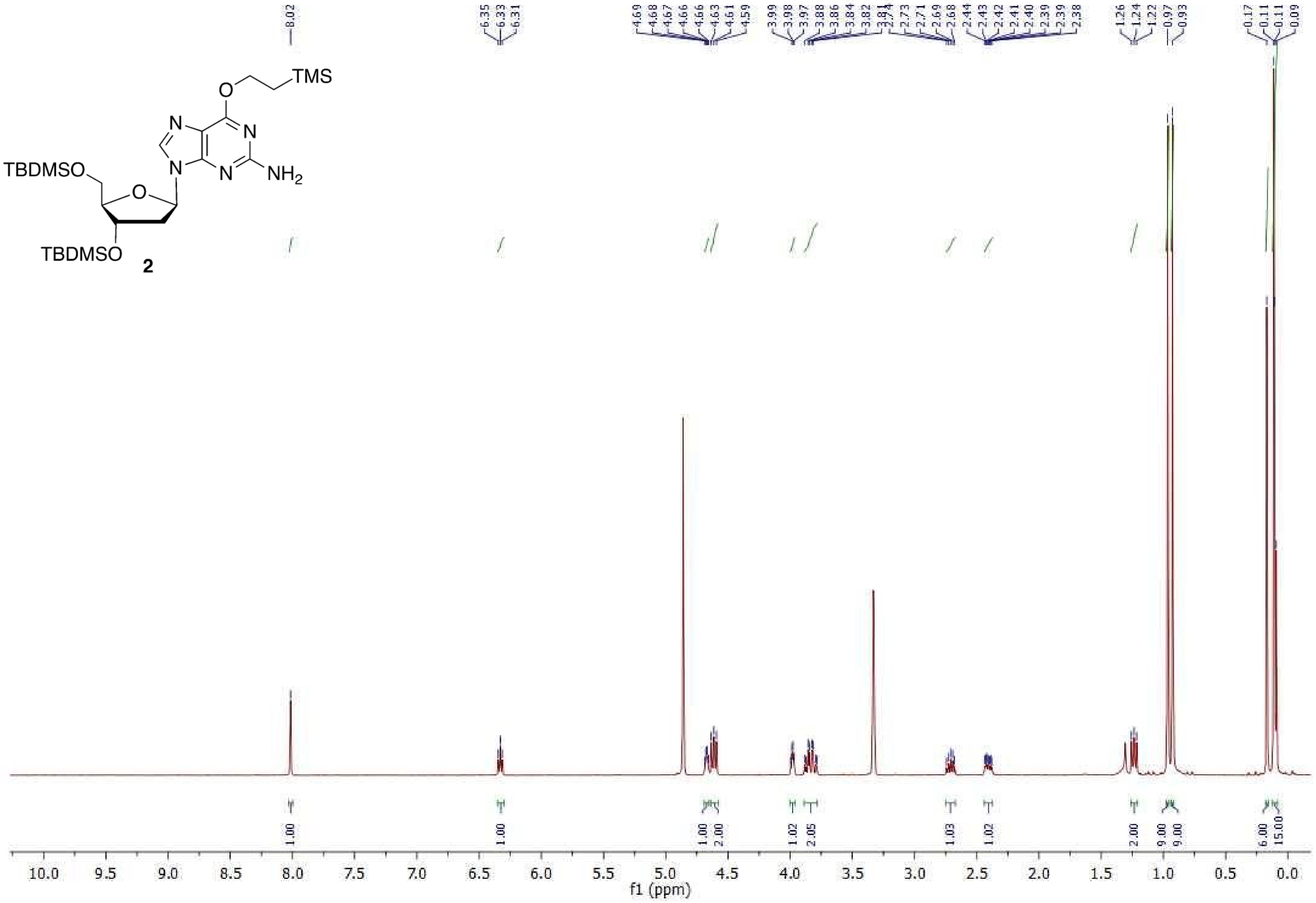

2

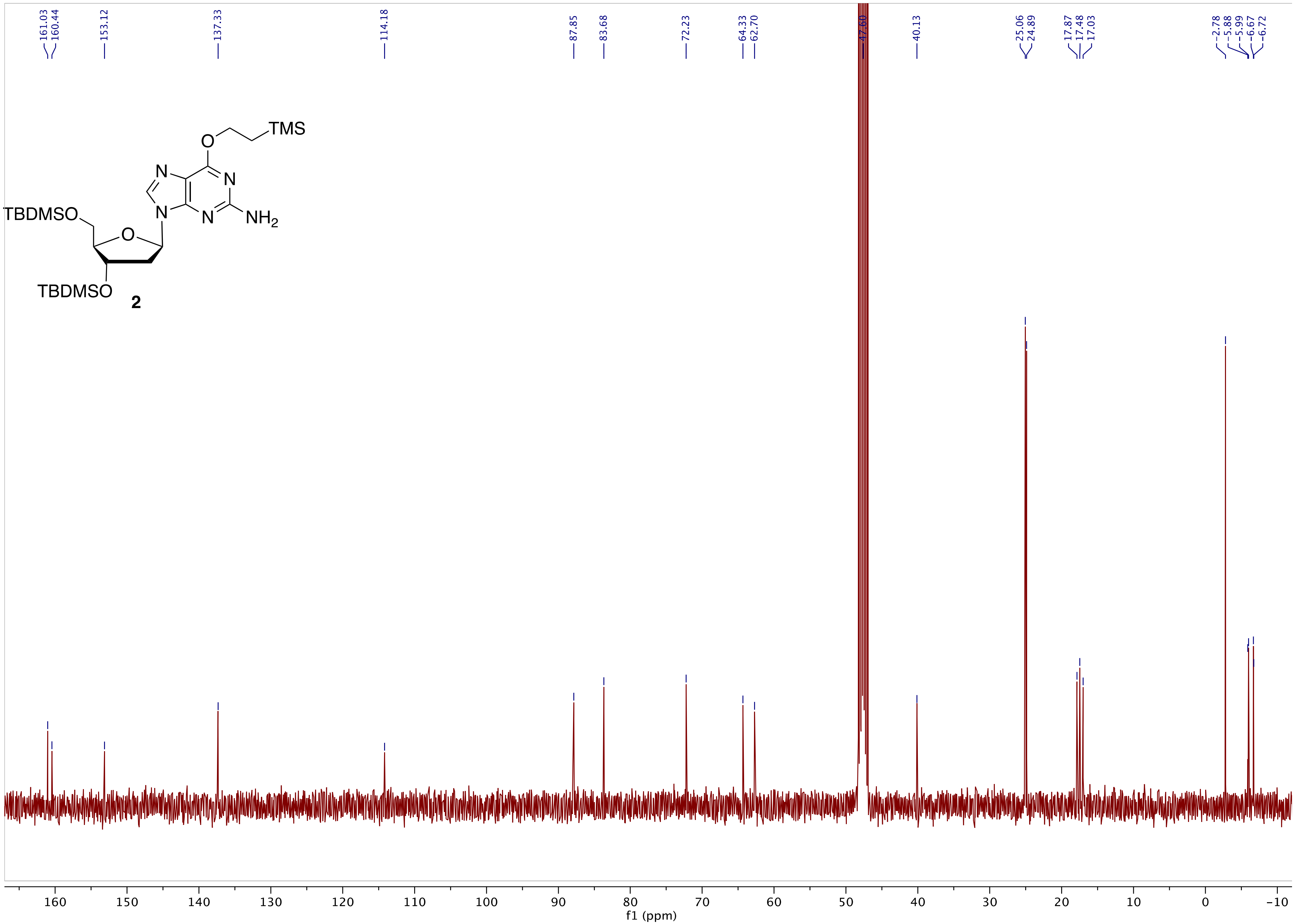
161.03
160.44
153.12
137.33
114.18
87.85
83.68
72.23
64.33
62.70
40.13
25.06
24.89
17.87
17.48
17.03
-2.78
-5.88
-5.99
-6.67
-6.72
TMS
TBDMSO
TBDMSO
NH2
2
f1 (ppm)

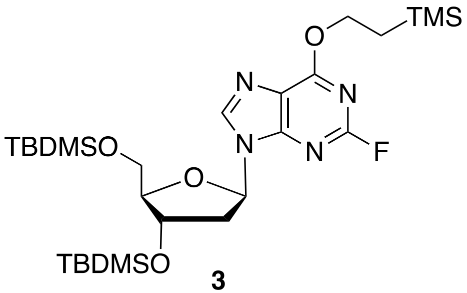
TMS
O
N
N
N
N
F
TBDMSO
O
TBDMSO

3

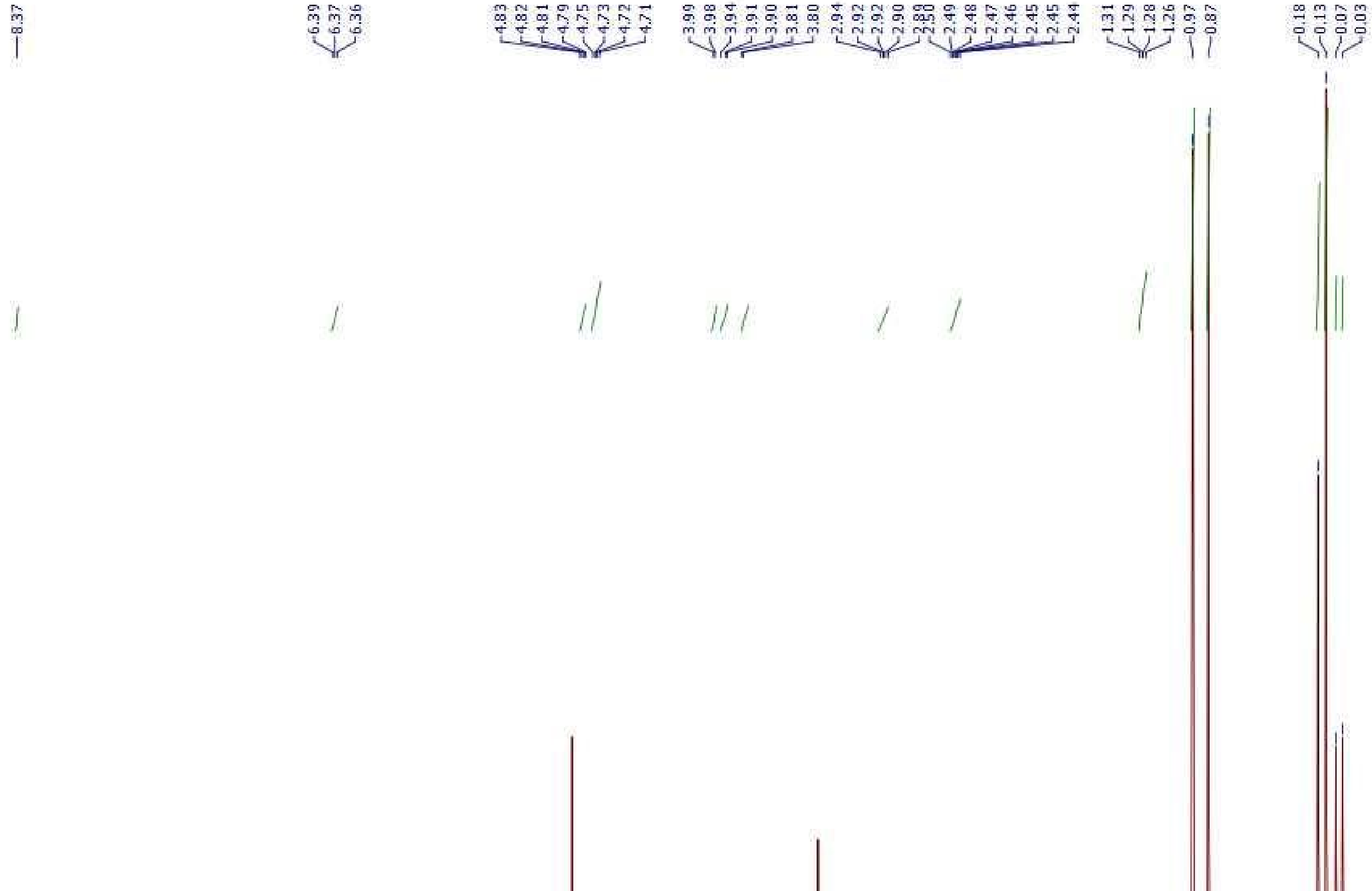
8.37
6.39
6.37
6.36
4.83
4.82
4.81
4.79
4.75
4.73
4.72
4.71
3.99
3.98
3.94
3.91
3.90
3.81
3.80
2.94
2.92
2.92
2.90
2.89
2.50
2.49
2.48
2.47
2.46
2.45
2.45
2.44
1.31
1.29
1.28
1.26
0.97
0.87
0.18
0.13
0.07
0.03

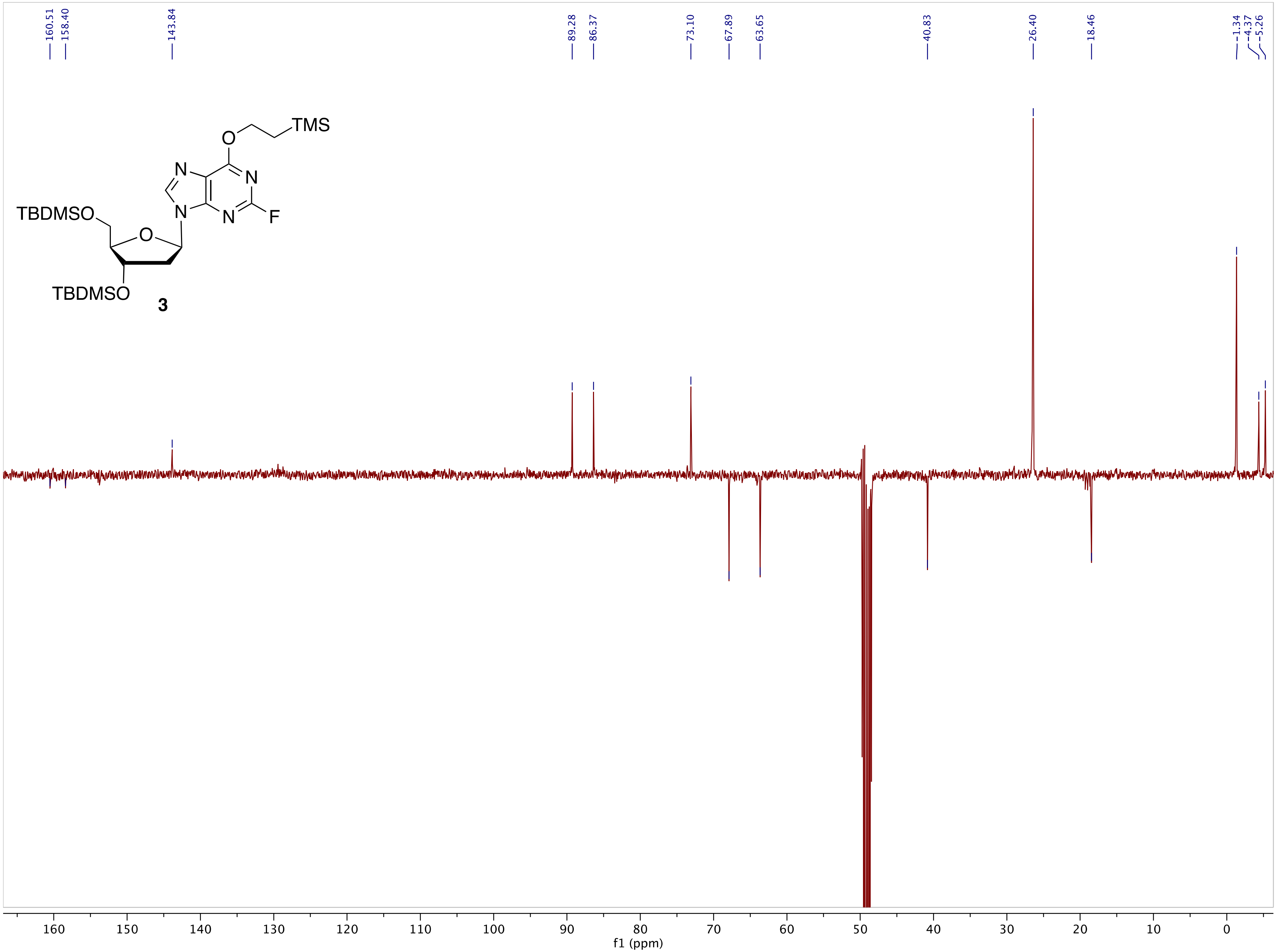
TMS
O
N
N
N
N
F
TBDMSO
O
TBDMSO
3
160.51
158.40
143.84
89.28
86.37
73.10
67.89
63.65
40.83
26.40
18.46
-1.34
-4.37
-5.26
160
150
140
130
120
110
100
90
80
70
60
50
40
30
20
10
0
f1 (ppm)

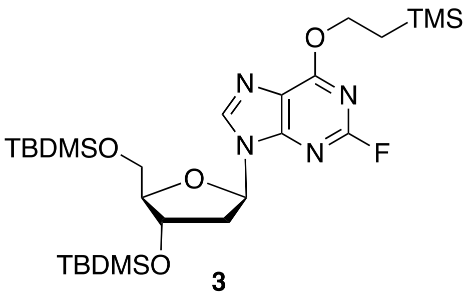

3

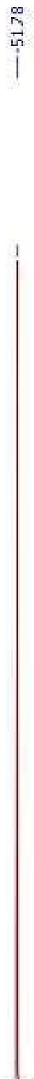

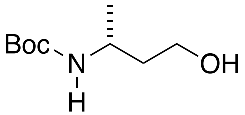
Boc
N
H
OH

4

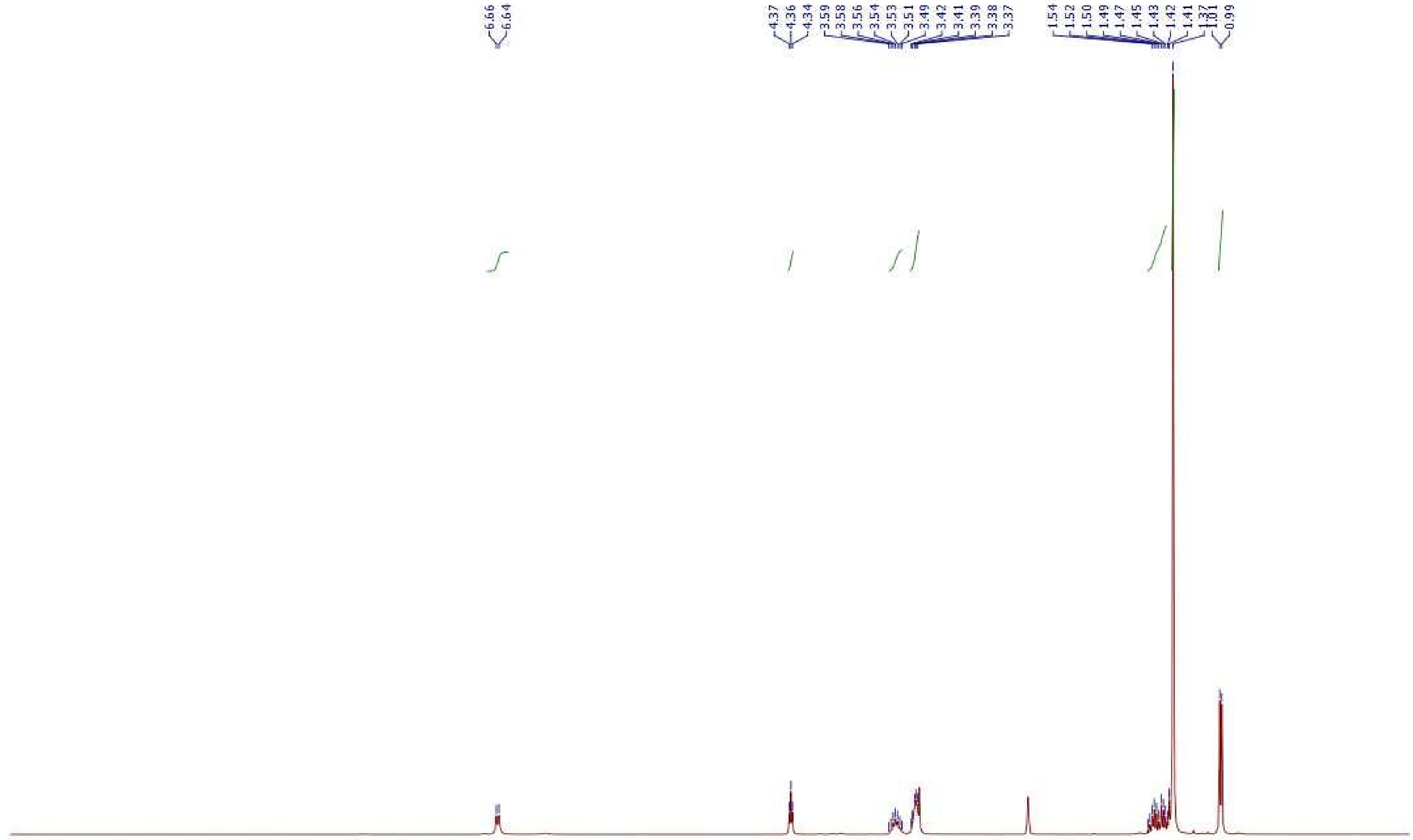
6.66
6.64
4.37
4.36
4.34
3.59
3.58
3.56
3.54
3.53
3.51
3.49
3.42
3.41
3.39
3.38
3.37
1.54
1.52
1.50
1.49
1.47
1.45
1.43
1.42
1.41
1.37
1.01
0.99

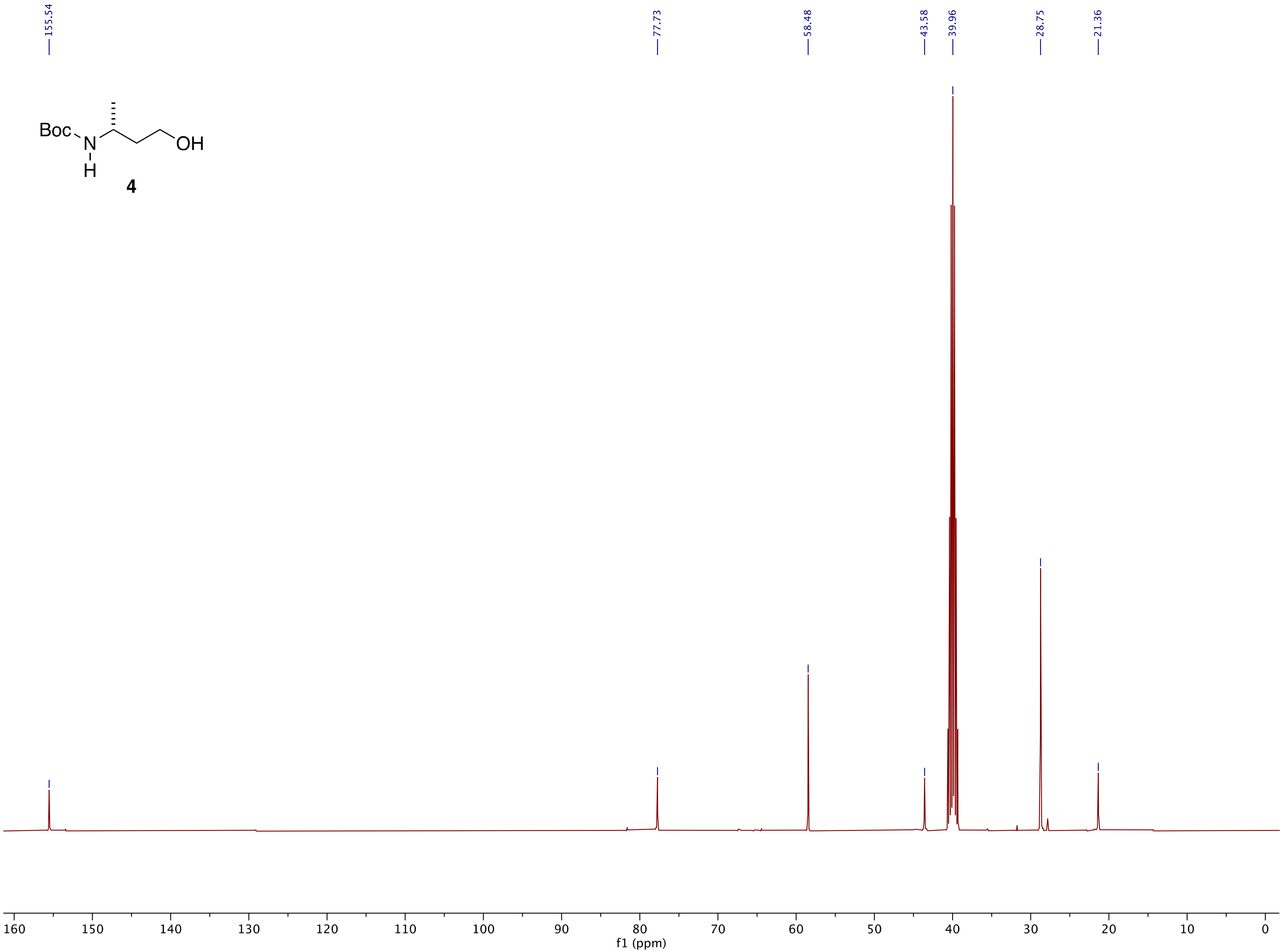
Boc
N
H
OH
4
155.54
77.73
58.48
43.58
39.96
28.75
21.36
160
150
140
130
120
110
100
90
80
70
60
50
40
30
20
10
0
f1 (ppm)

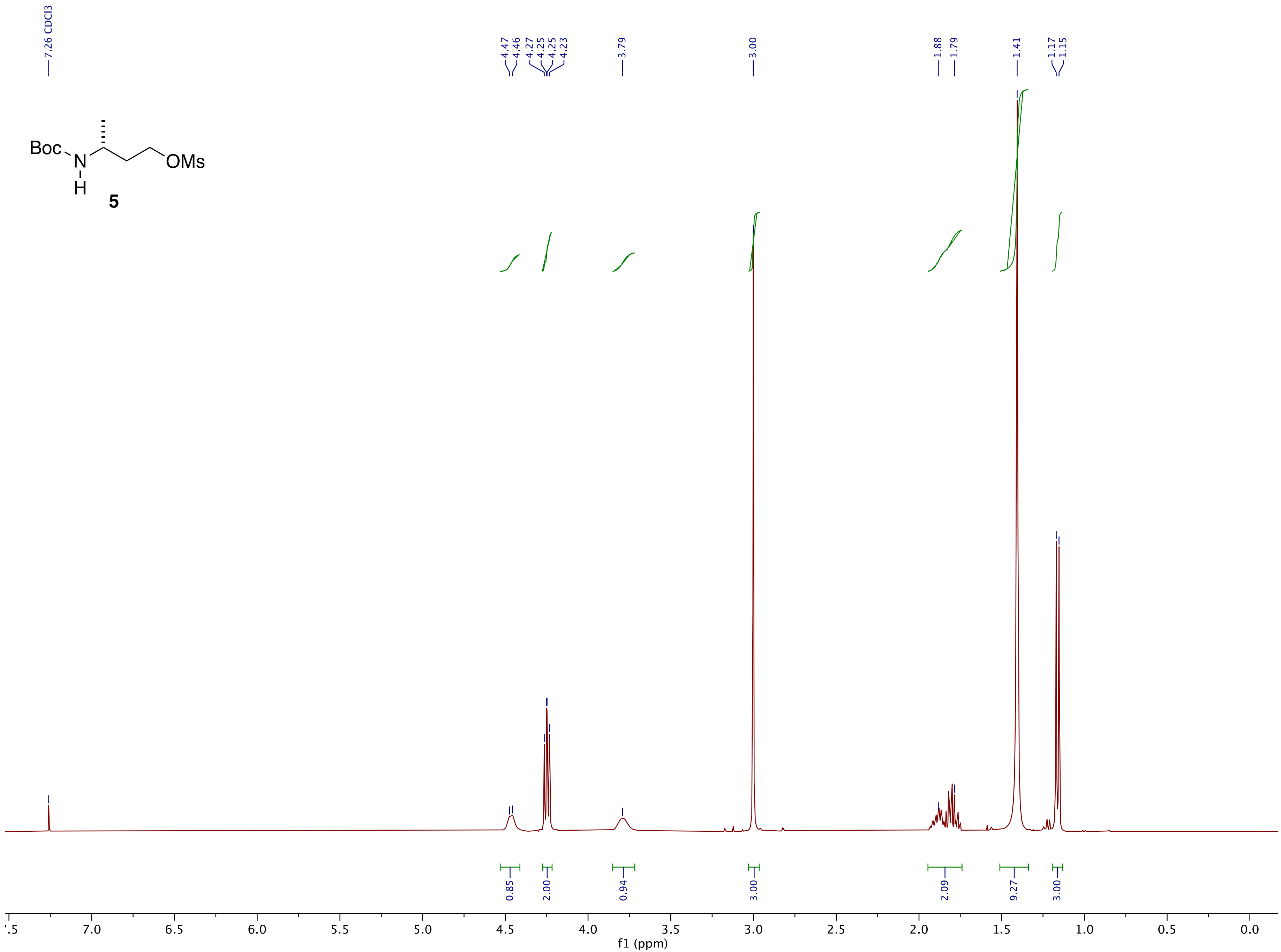

7.26 CDCl3
4.47
4.46
4.27
4.25
4.25
4.23
3.79
3.00
1.88
1.79
1.41
1.17
1.15
Boc
N
H
OMs
5
0.85
2.00
0.94
3.00
2.09
9.27
3.00
7.0
6.5
6.0
5.5
5.0
4.5
4.0
3.5
3.0
2.5
2.0
1.5
1.0
0.5
0.0
f1 (ppm)

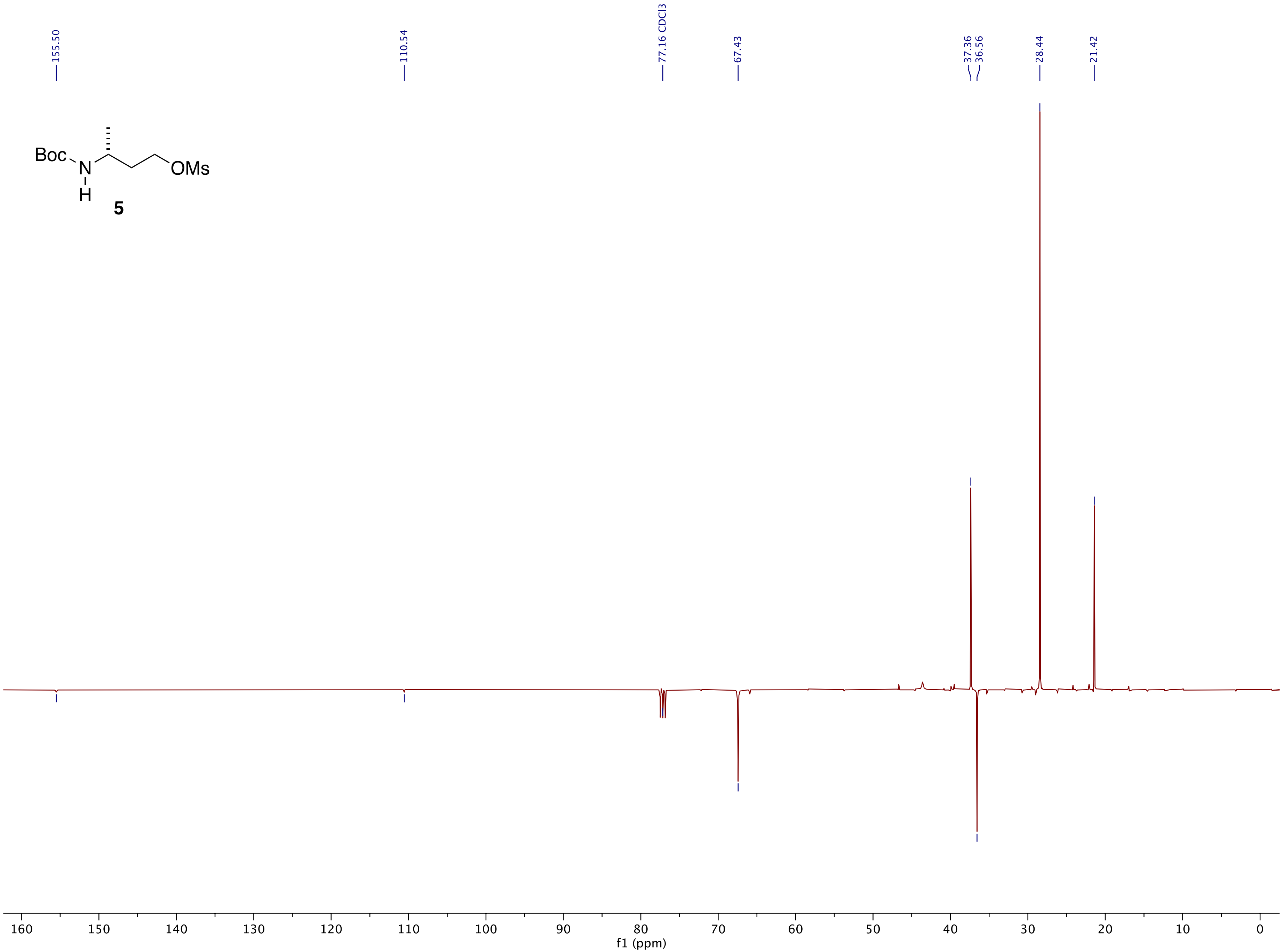
Boc
N
H
OMs
5
155.50
110.54
77.16 CDCl3
67.43
37.36
36.56
28.44
21.42
160
150
140
130
120
110
100
90
80
70
60
50
40
30
20
10
0
f1 (ppm)

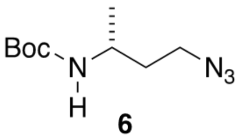
Boc
N
H
N3

6

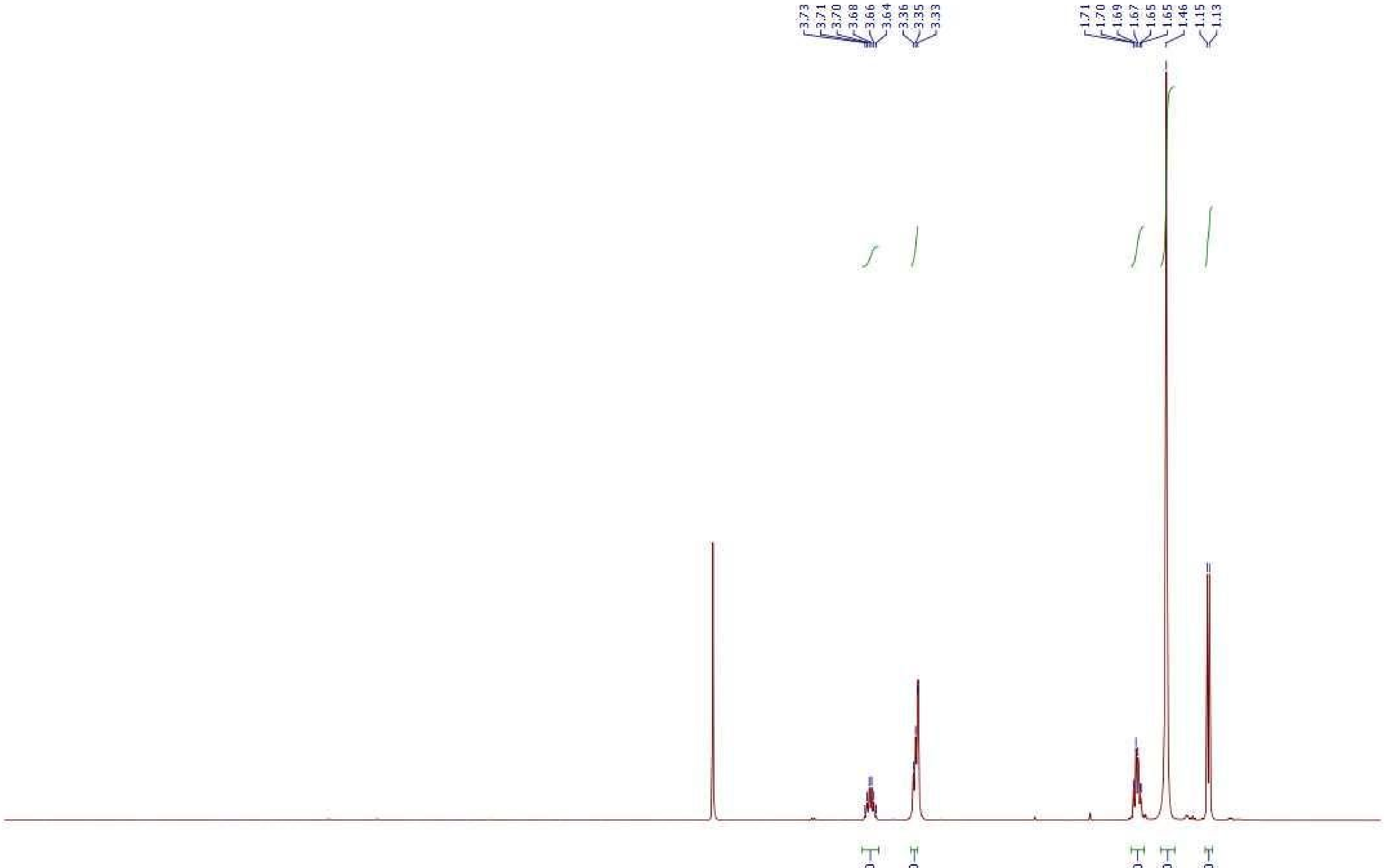
3.73
3.71
3.70
3.68
3.66
3.64
3.36
3.35
3.33
1.71
1.70
1.69
1.67
1.65
1.65
1.46
1.15
1.13

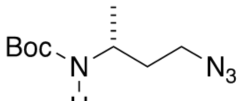

6

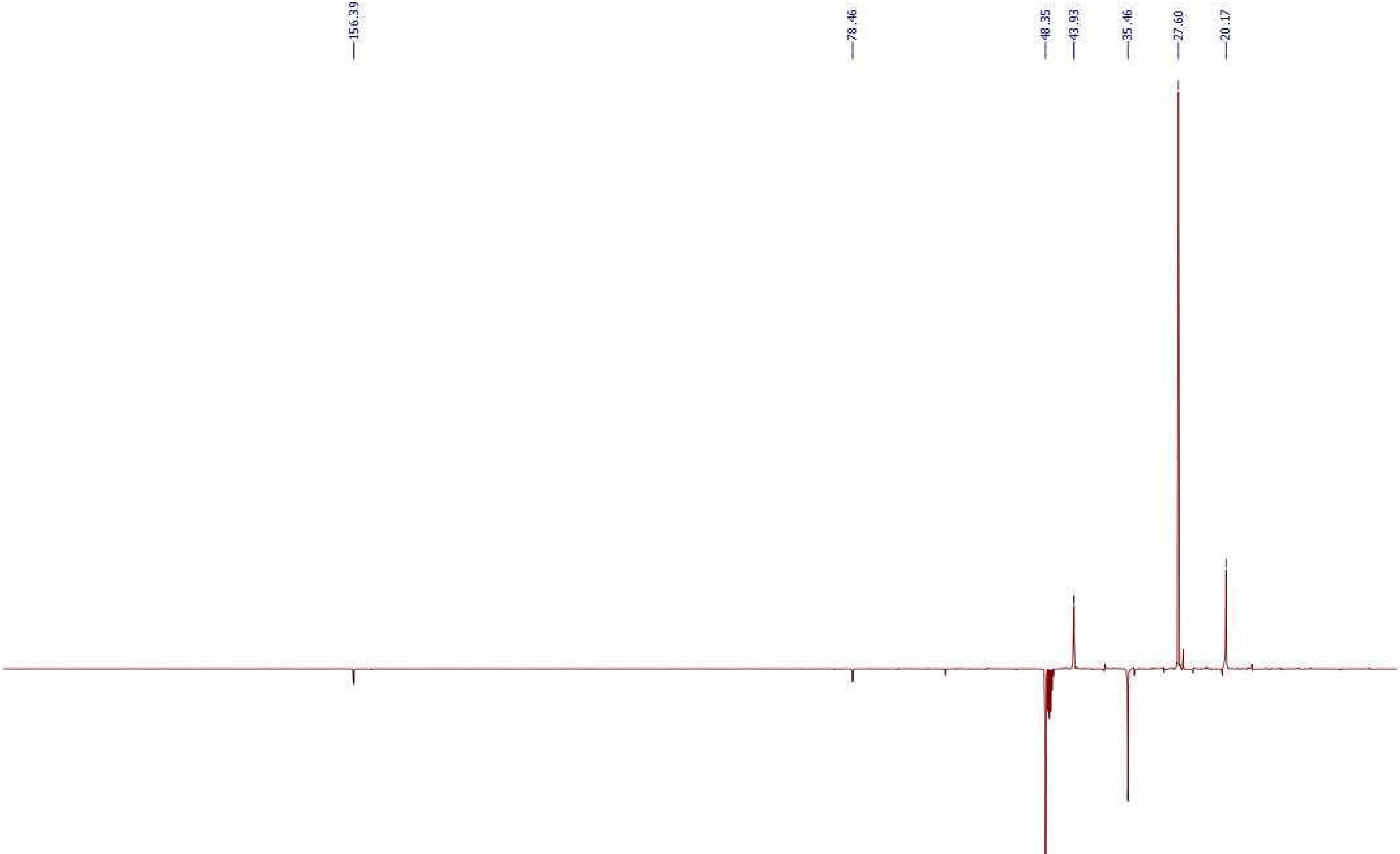

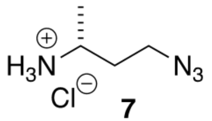
H3N
Cl
N3

7

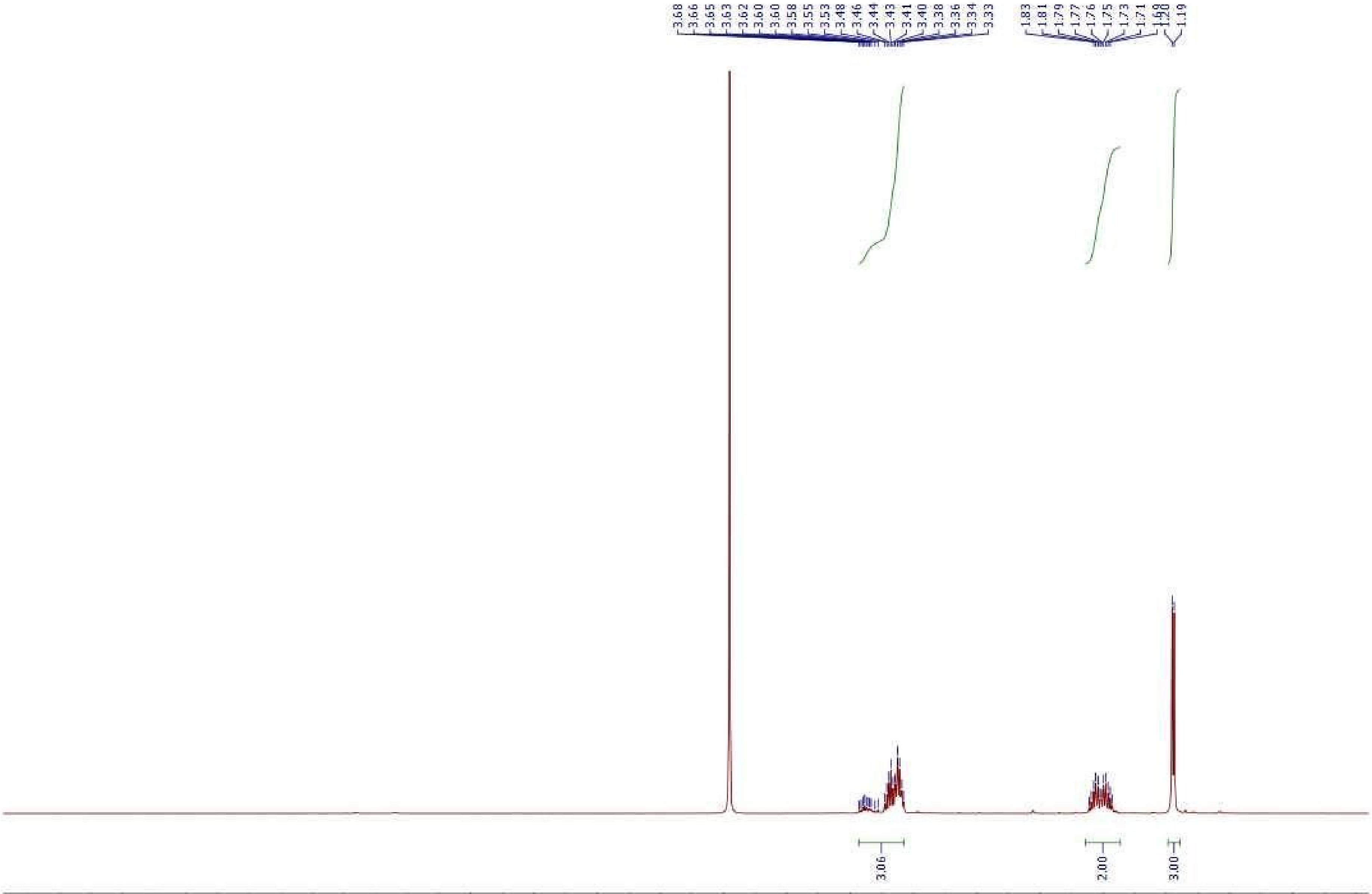
3.68 3.66 3.65 3.63 3.62 3.60 3.60 3.58 3.55 3.53 3.48 3.46 3.44 3.43 3.41 3.40 3.38 3.36 3.34 3.33
1.83 1.81 1.79 1.77 1.76 1.75 1.73 1.71 1.69 1.20 1.19
3.06
2.00
3.00

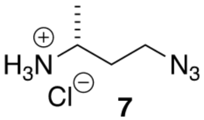

7

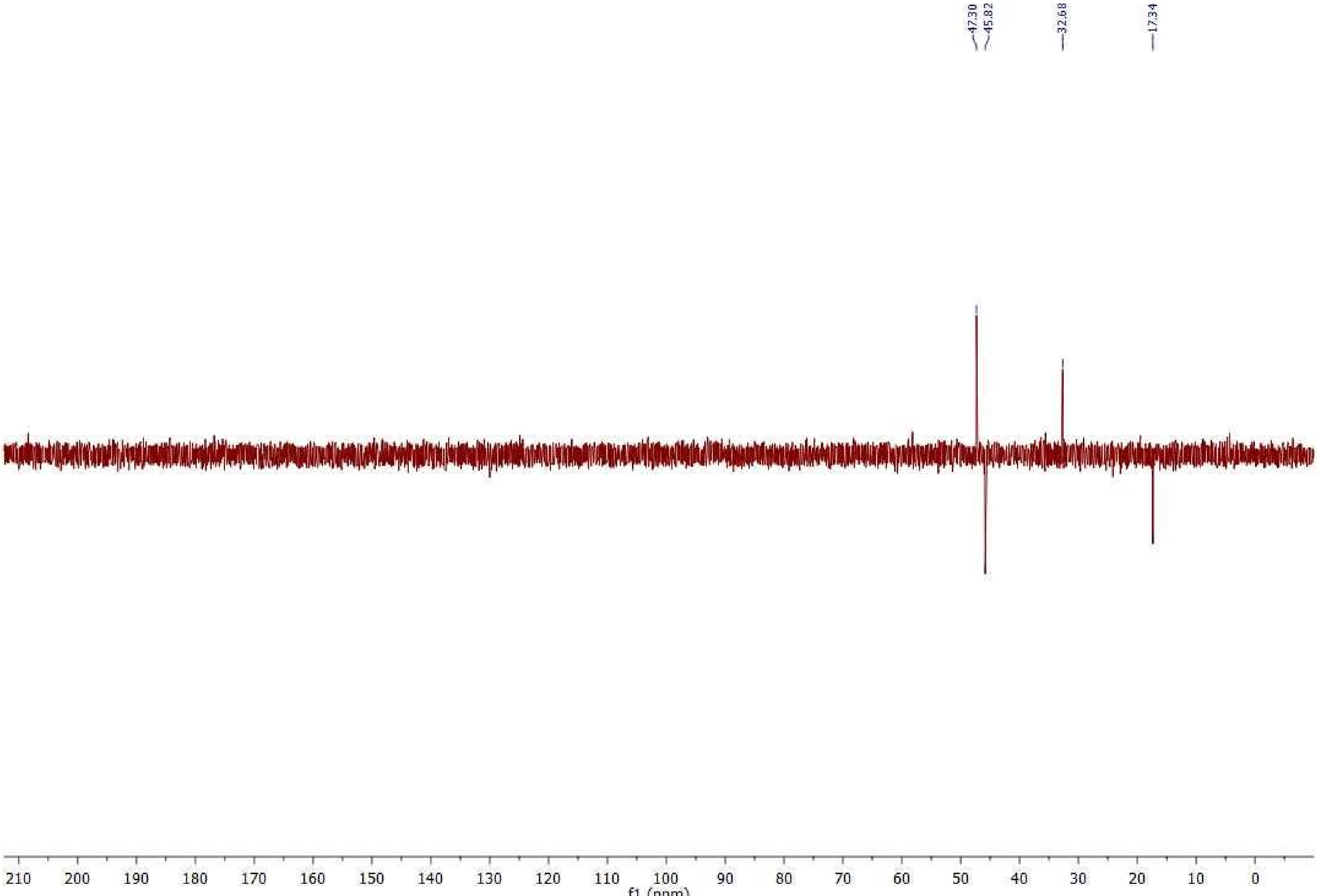

47.30
45.82
32.68
17.34
210 200 190 180 170 160 150 140 130 120 110 100 90 80 70 60 50 40 30 20 10 0
f1 (ppm)

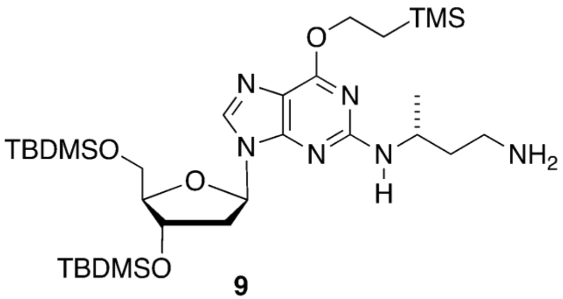

9

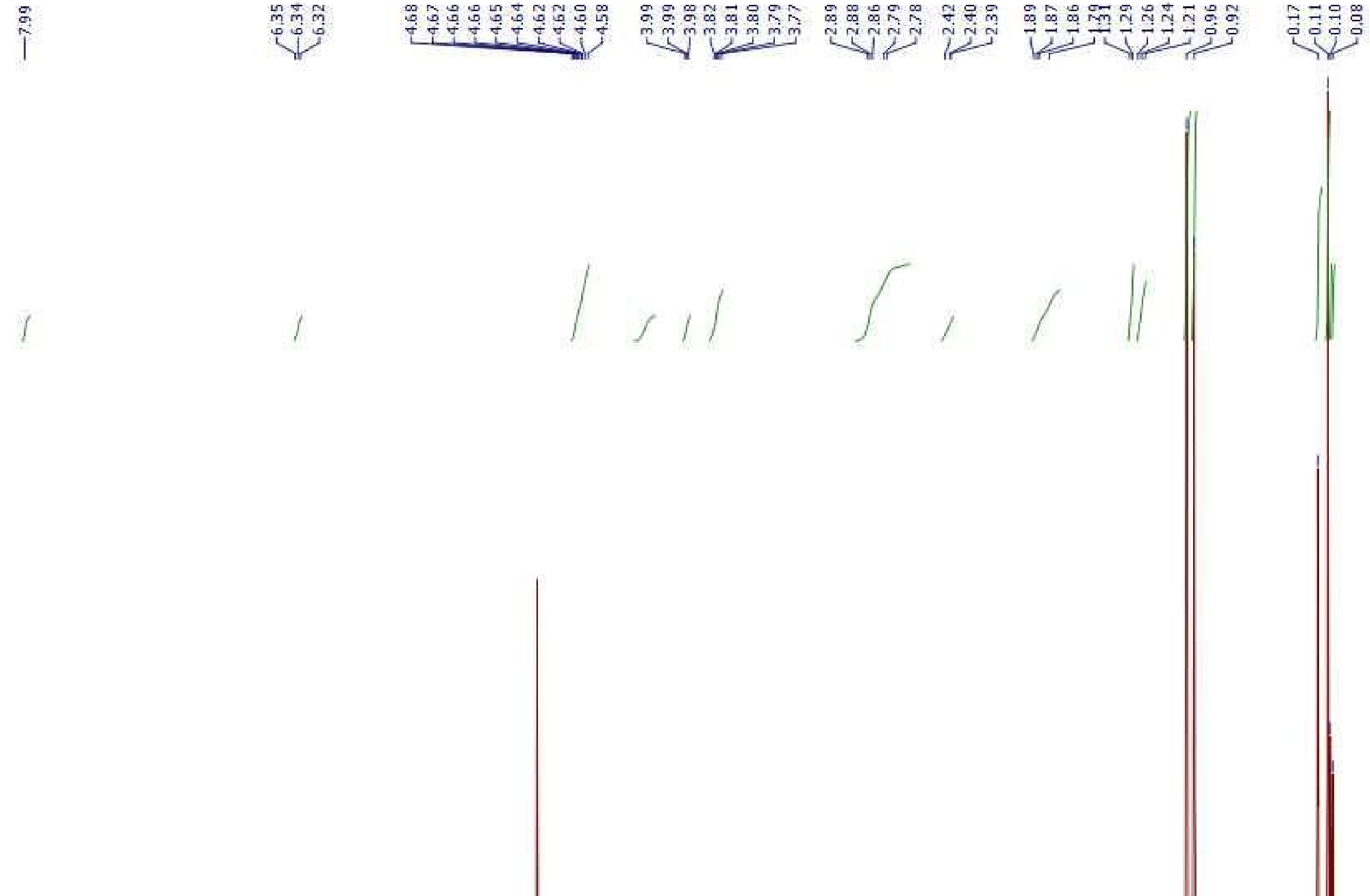

7.99
6.35
6.34
6.32
4.68
4.67
4.66
4.66
4.65
4.64
4.62
4.62
4.60
4.58
3.99
3.99
3.98
3.82
3.81
3.80
3.79
3.77
2.89
2.88
2.86
2.79
2.78
2.42
2.40
2.39
1.89
1.87
1.86
1.31
1.29
1.26
1.24
1.21
0.96
0.92
0.17
0.11
0.10
0.08

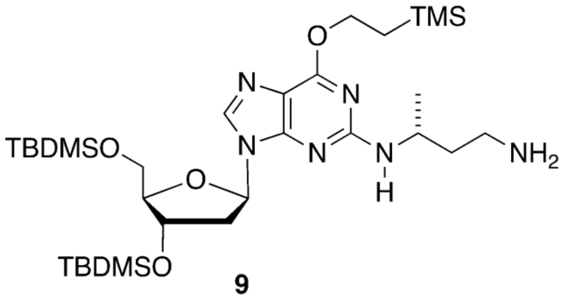
TMS
O
N
N
TBDMSO
N
N
N
NH$_2$
H
O
TBDMSO

9

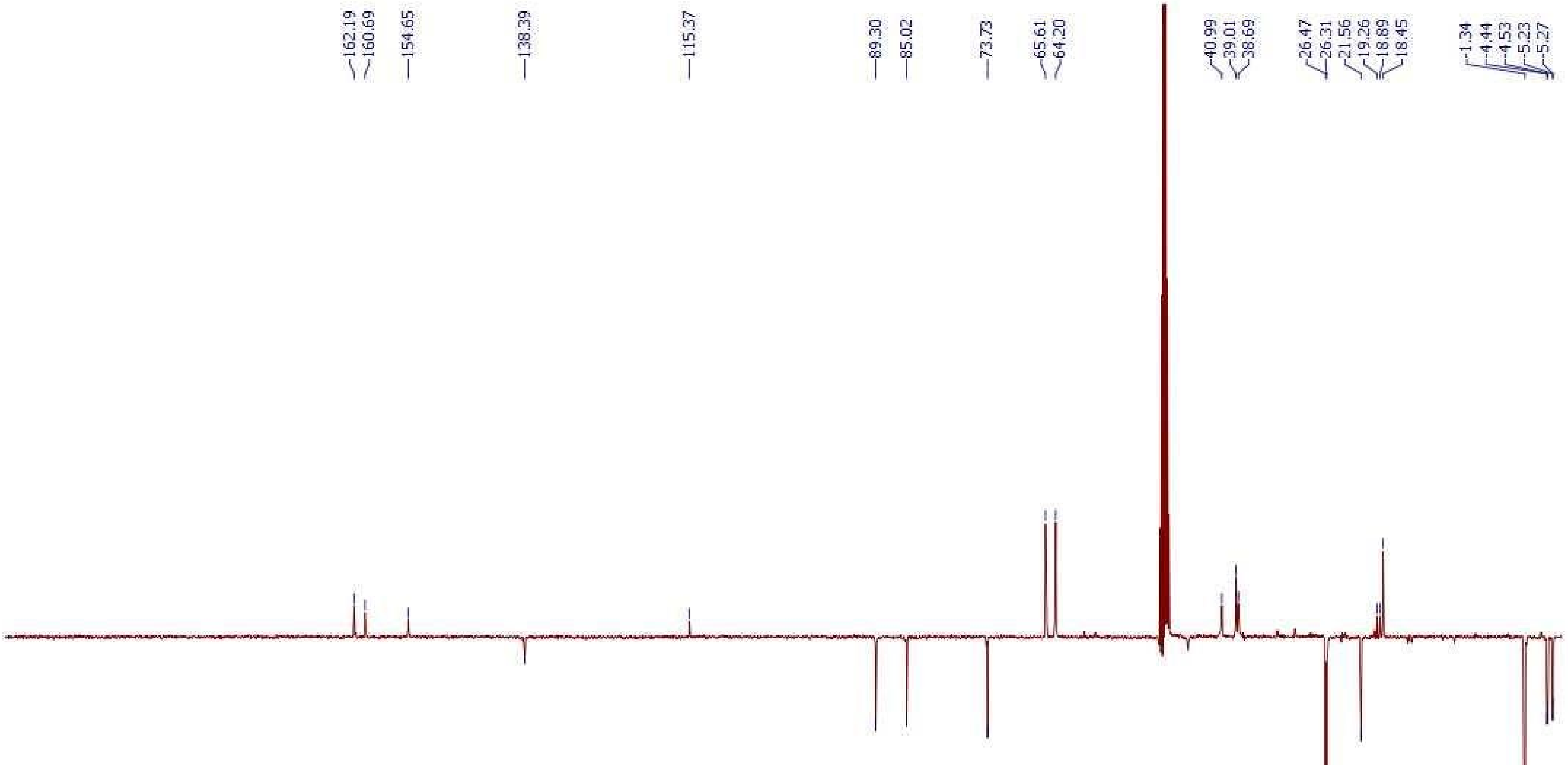
162.19
160.69
154.65
138.39
115.37
89.30
85.02
73.73
65.61
64.20
40.99
39.01
38.69
26.47
26.31
21.56
19.26
18.89
18.45
-1.34
-4.44
-4.53
-5.23
-5.27

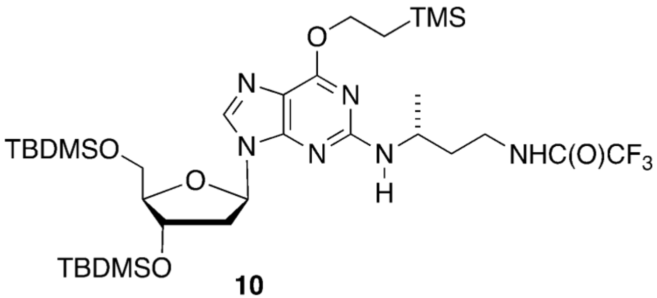

TMS
O
N
TBDMSO
N
H
NHC(O)CF3
TBDMSO

10

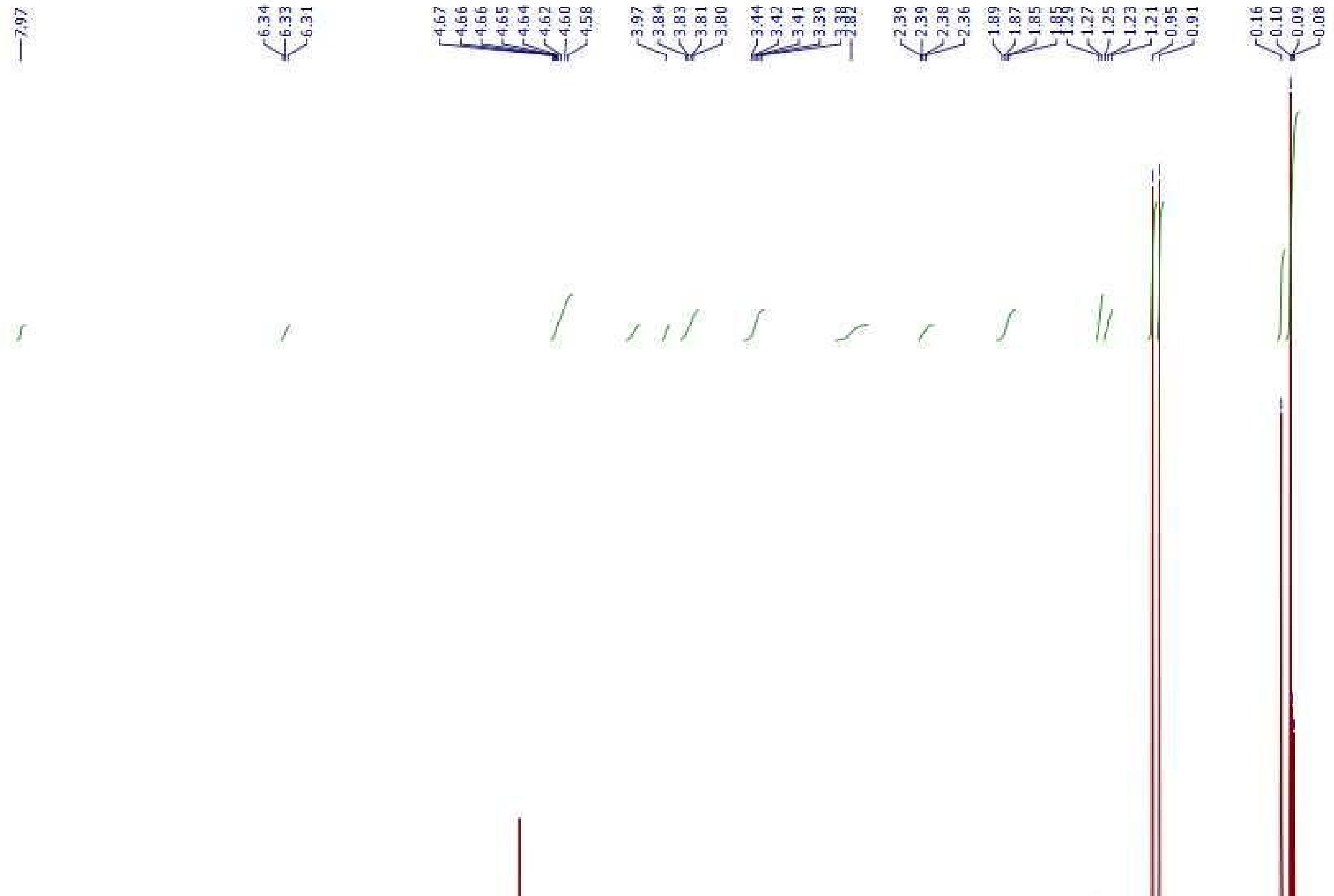

7.97
6.34
6.33
6.31
4.67
4.66
4.66
4.65
4.64
4.62
4.60
4.58
3.97
3.84
3.83
3.81
3.80
3.44
3.42
3.41
3.39
2.39
2.39
2.38
2.36
1.89
1.87
1.85
1.27
1.25
1.23
1.21
0.95
0.91
0.16
0.10
0.09
0.08

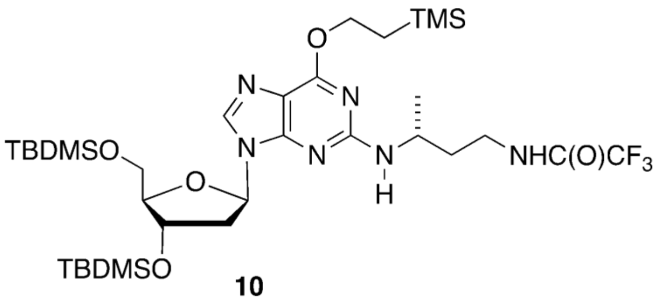

10

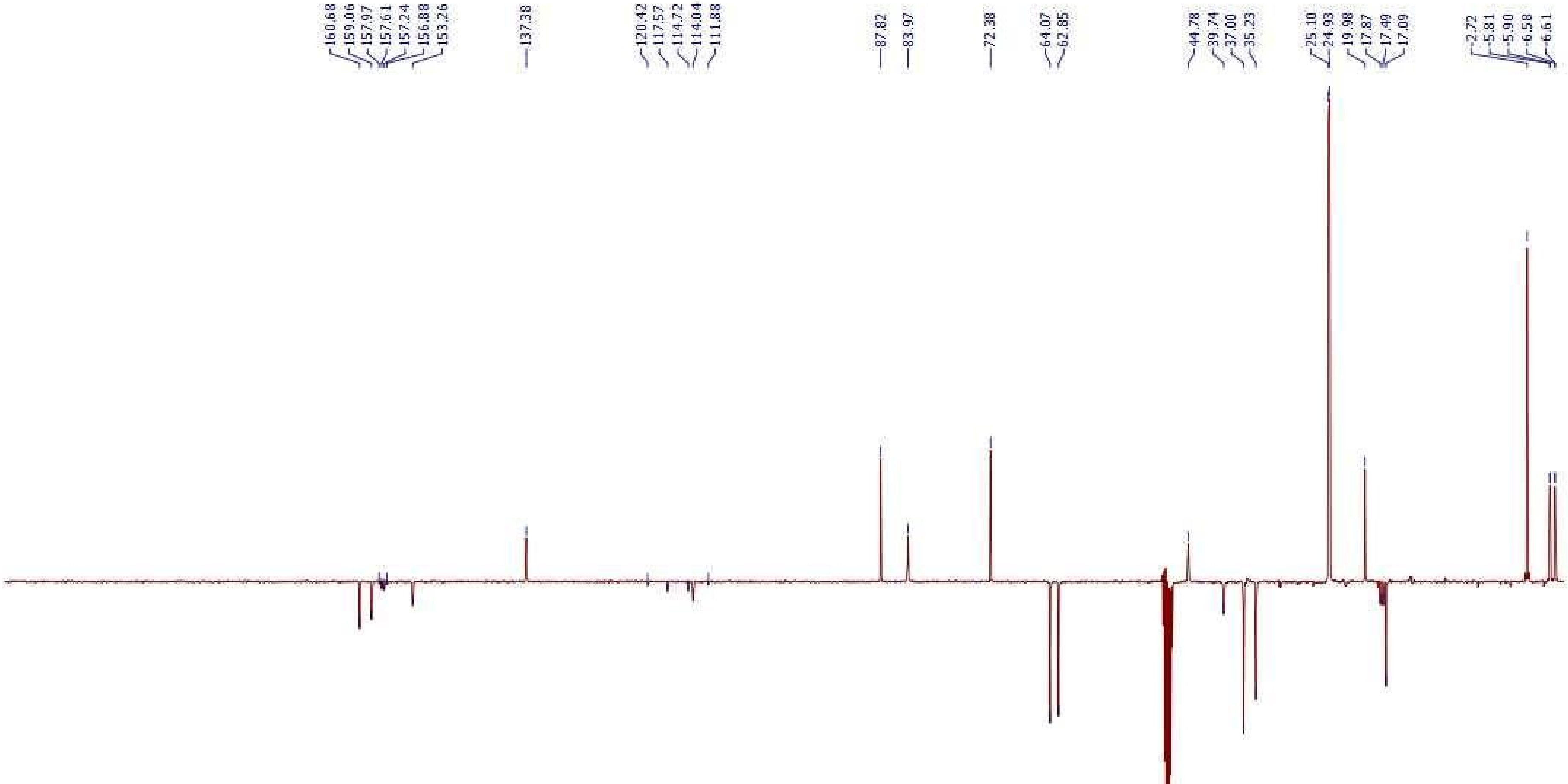
160.68
159.06
157.97
157.61
157.24
156.88
153.26
137.38
120.42
117.57
114.72
114.04
111.88
87.82
83.97
72.38
64.07
62.85
44.78
39.74
37.00
35.23
25.10
24.93
19.98
17.87
17.49
17.09
-2.72
-5.81
-5.90
-6.58
-6.61

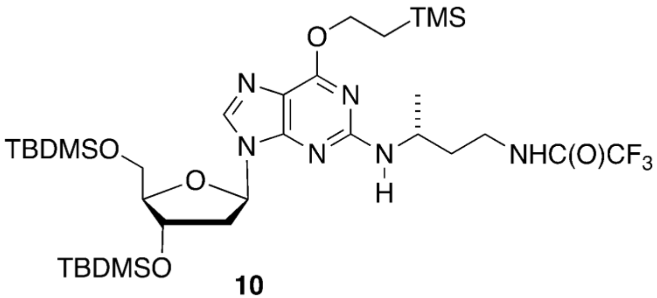

10

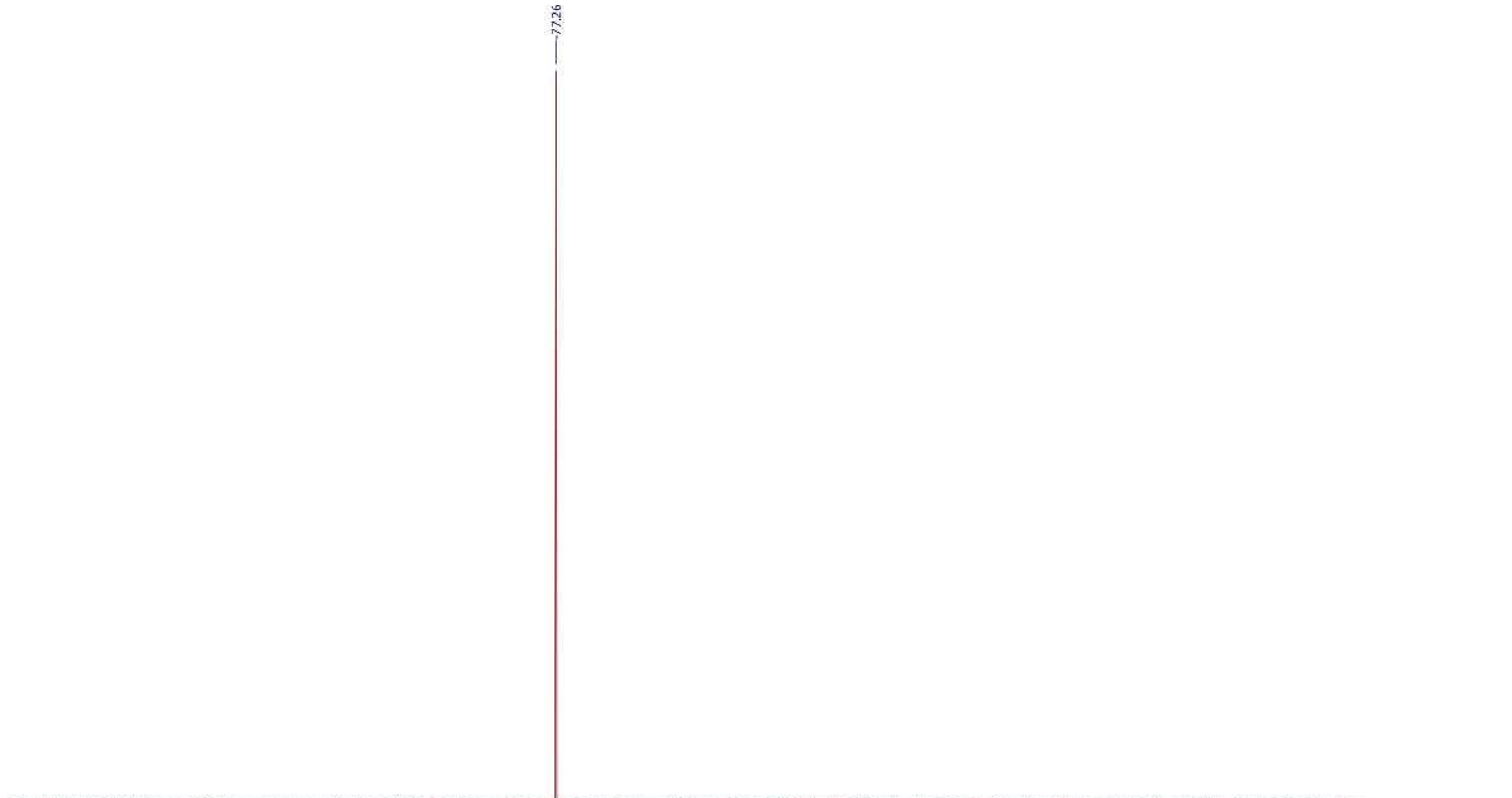

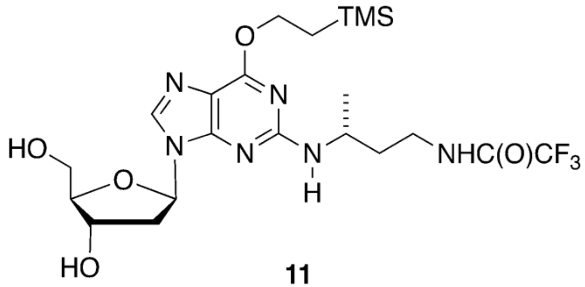
TMS
O
N
N
N
N
N
HO
O
HO
N
H
NHC(O)CF3

11

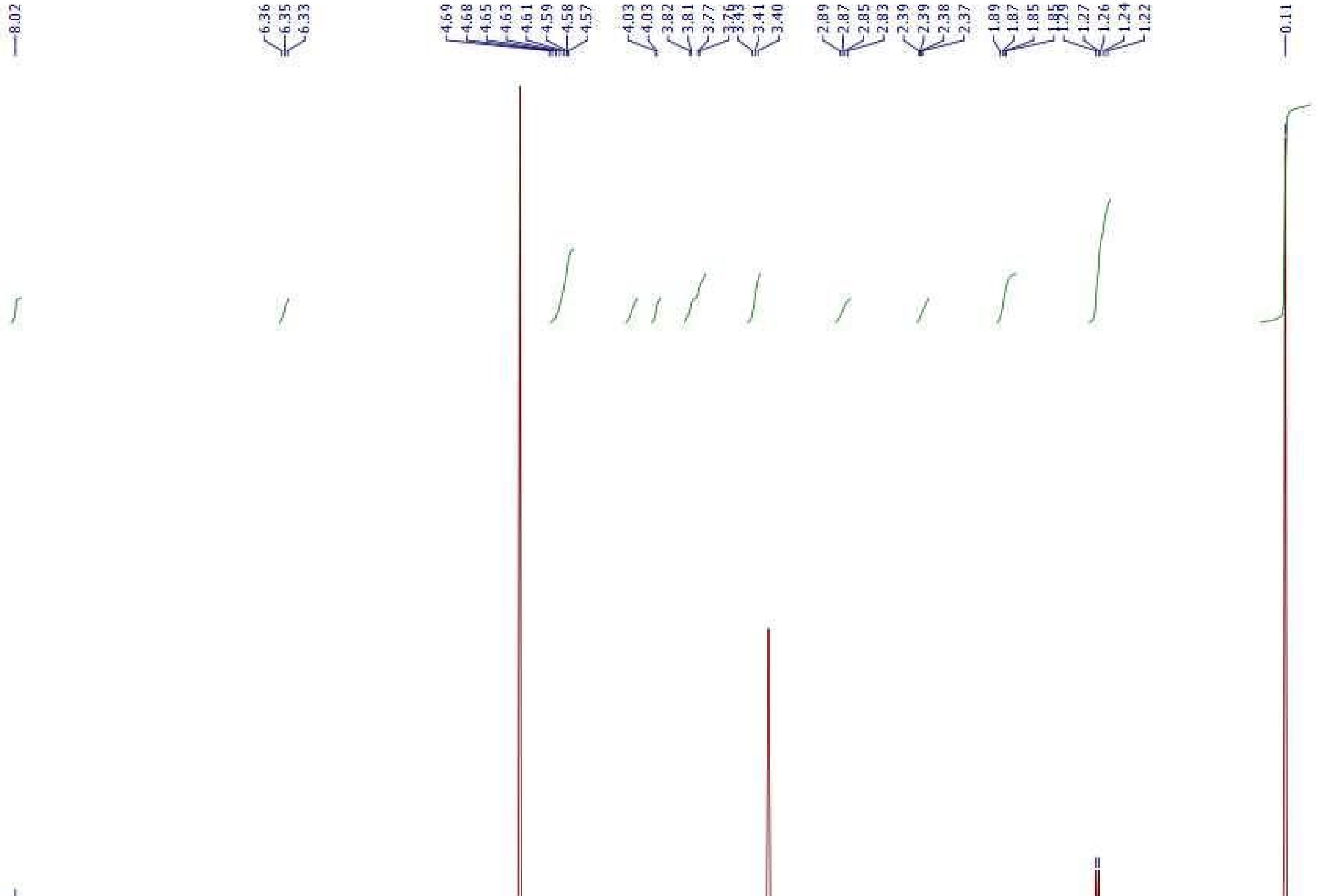
8.02
6.36
6.35
6.33
4.69
4.68
4.65
4.63
4.61
4.59
4.58
4.57
4.03
4.03
3.82
3.81
3.77
3.41
3.40
2.89
2.87
2.85
2.83
2.39
2.39
2.38
2.37
1.89
1.87
1.85
1.27
1.26
1.24
1.22
0.11

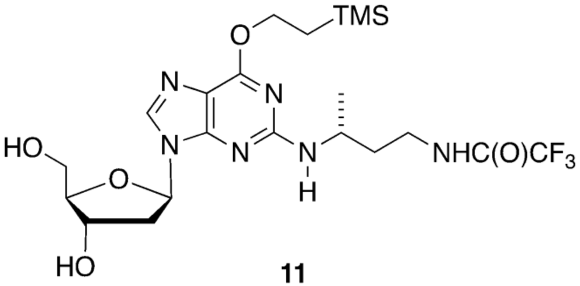
TMS
O
N
N
N
N
HO
O
N
H
NHC(O)CF3
HO

11

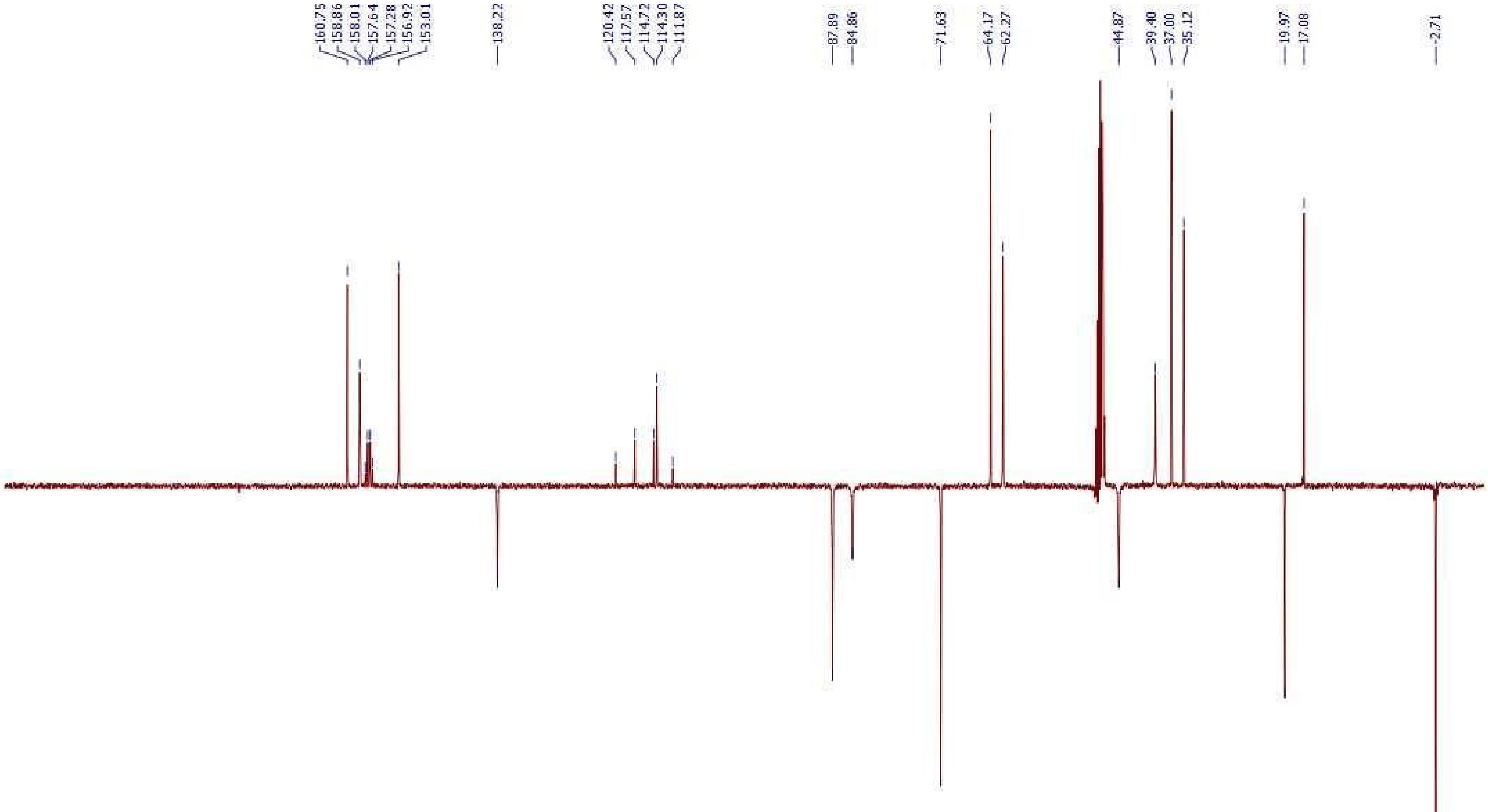
160.75
158.86
158.01
157.64
157.28
156.92
153.01
138.22
120.42
117.57
114.72
114.30
111.87
87.89
84.86
71.63
64.17
62.27
44.87
39.40
37.00
35.12
19.97
17.08
-2.71

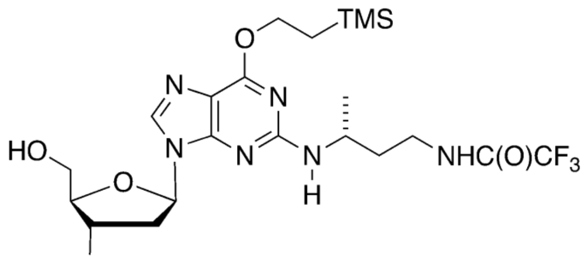

11

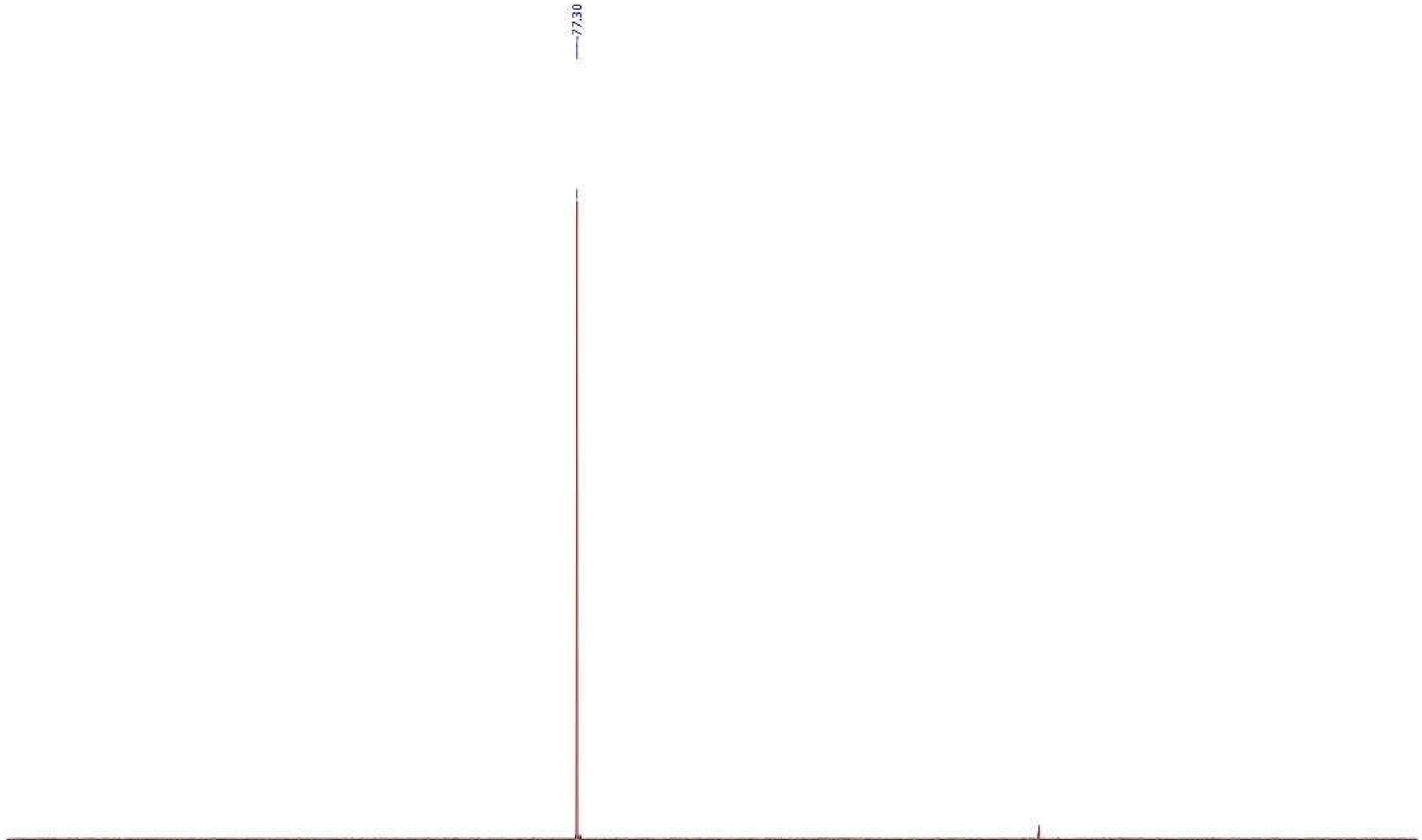

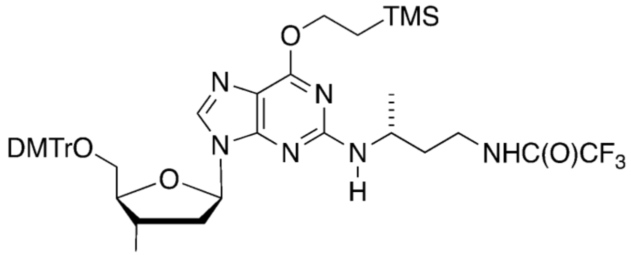
TMS
O
N
N
DMTrO
N
N
N
NHC(O)CF$_3$
H
O
HO

12

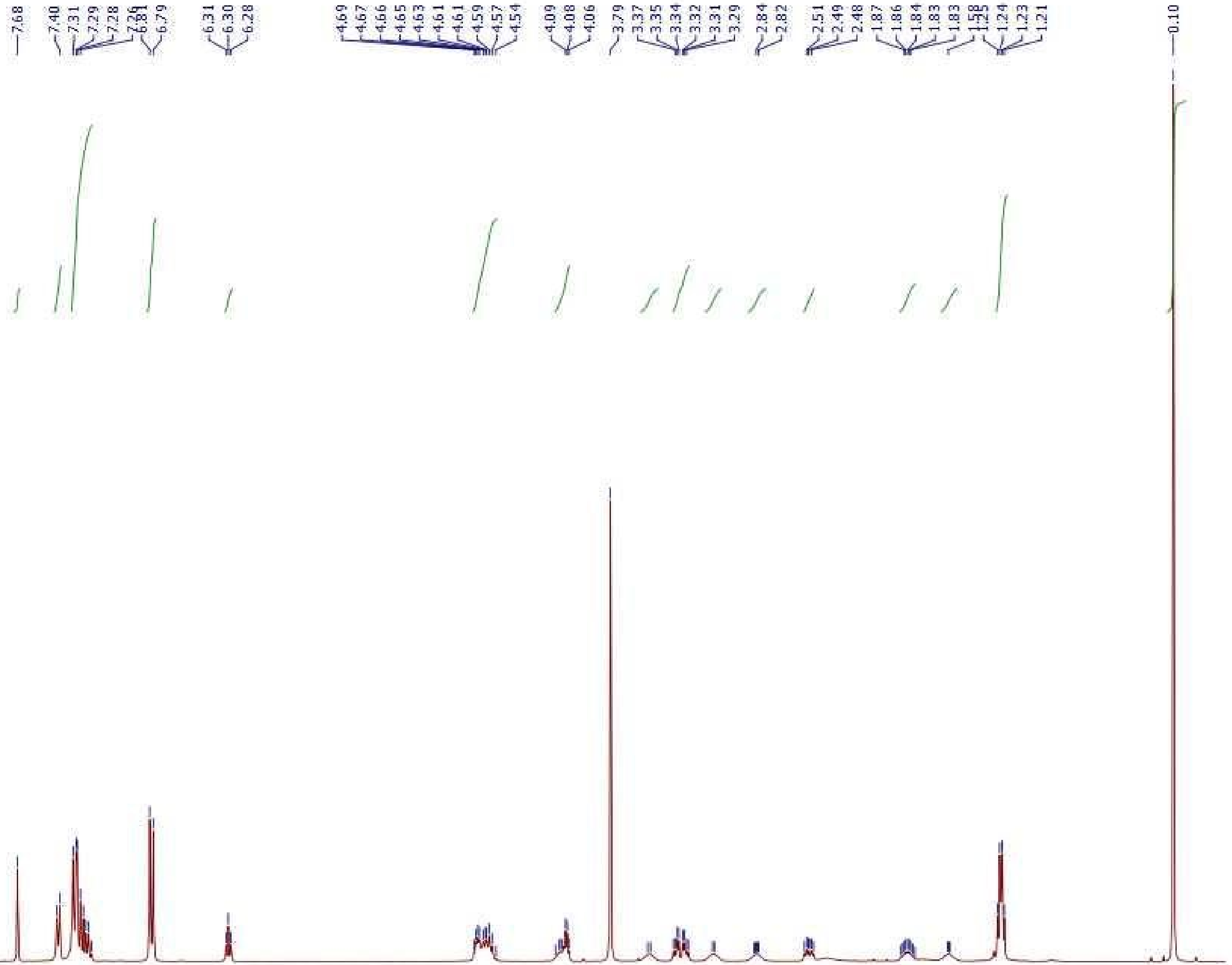
7.68
7.40
7.31
7.29
7.28
7.26
6.81
6.79
6.31
6.30
6.28
4.69
4.67
4.66
4.65
4.63
4.61
4.61
4.59
4.57
4.54
4.09
4.08
4.06
3.79
3.37
3.35
3.34
3.32
3.31
3.29
2.84
2.82
2.51
2.49
2.48
1.87
1.86
1.84
1.83
1.83
1.58
1.25
1.24
1.23
1.21
0.10

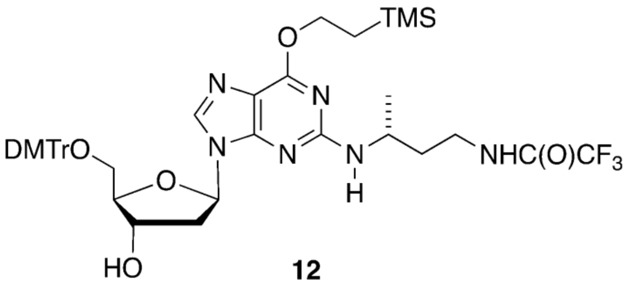
TMS
DMTrO
HO
NHC(O)CF3

12

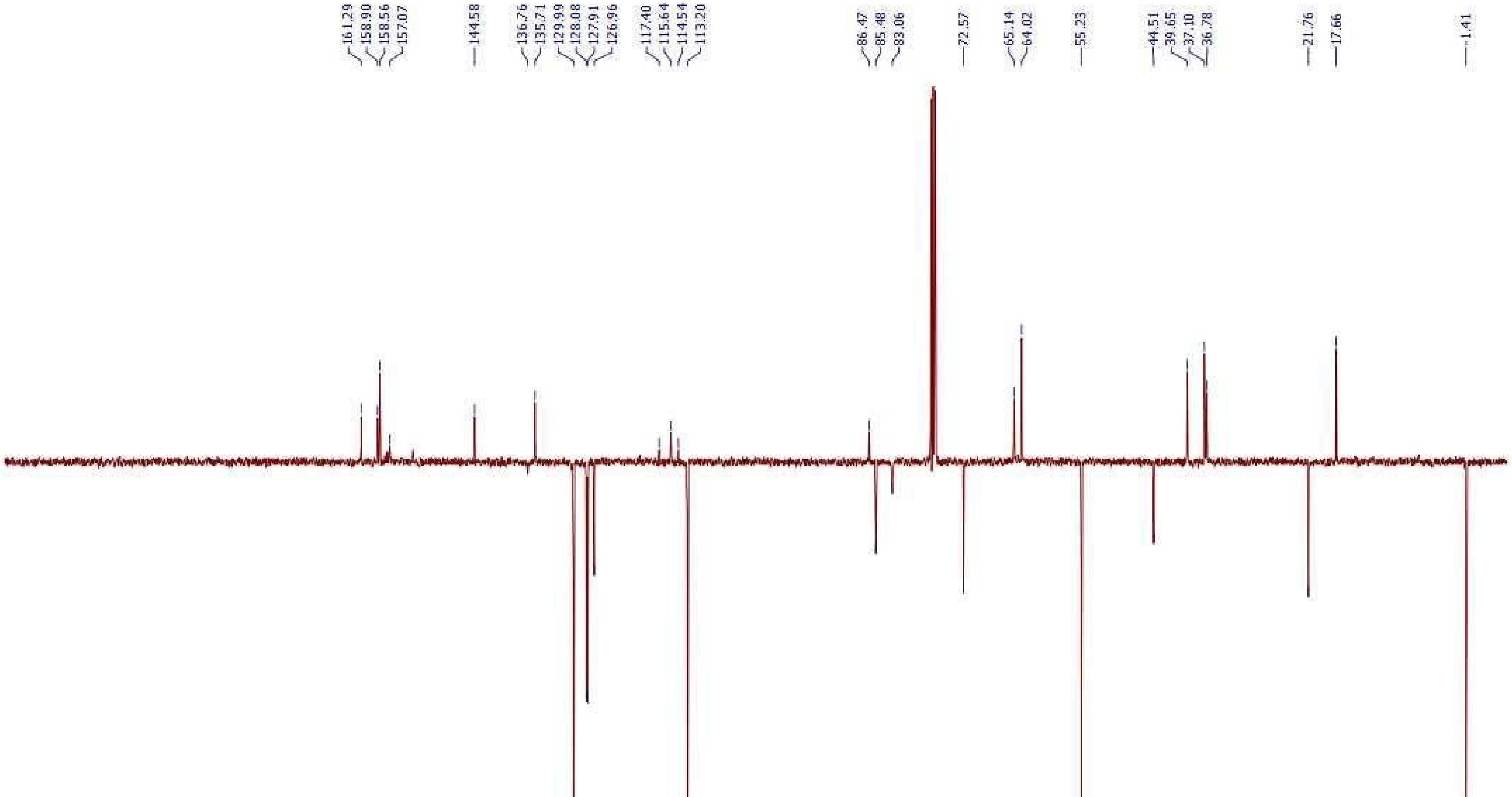
161.29
158.90
158.56
157.07
144.58
136.76
135.71
129.99
128.08
127.91
126.96
117.40
115.64
114.54
113.20
86.47
85.48
83.06
72.57
65.14
64.02
55.23
44.51
39.65
37.10
36.78
21.76
17.66
1.41

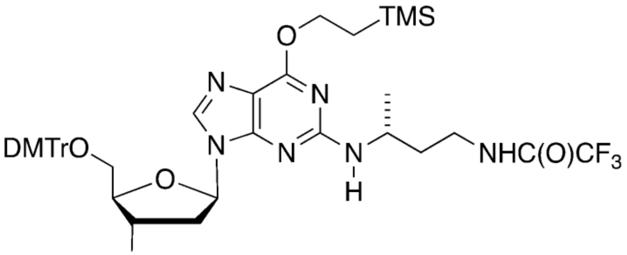

12

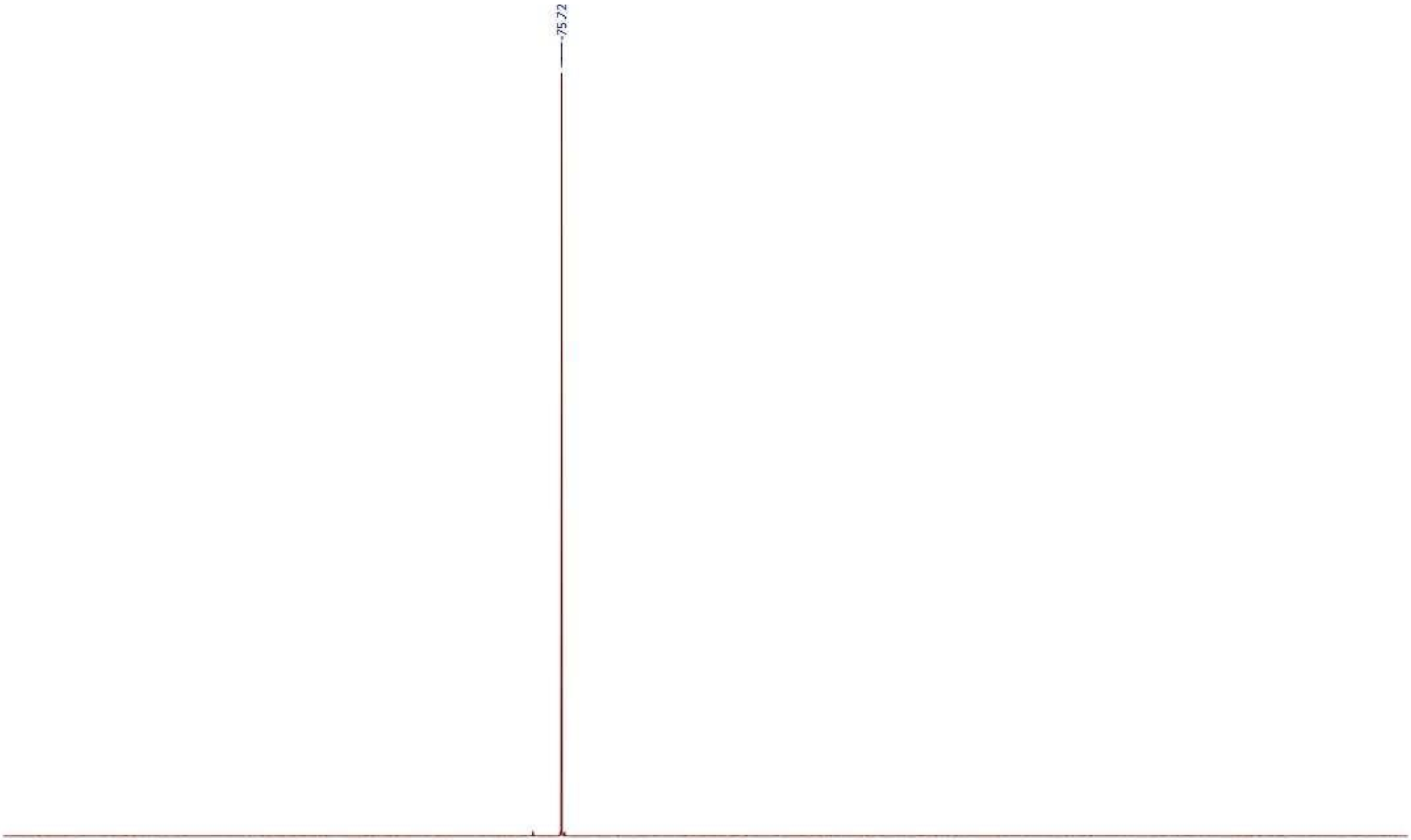

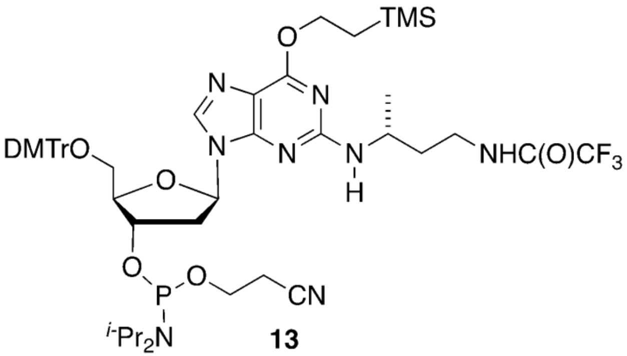
TMS
O
N
N
N
N
DMTrO
O
N
H
NHC(O)CF3
O
P
O
CN
i-Pr2N

13

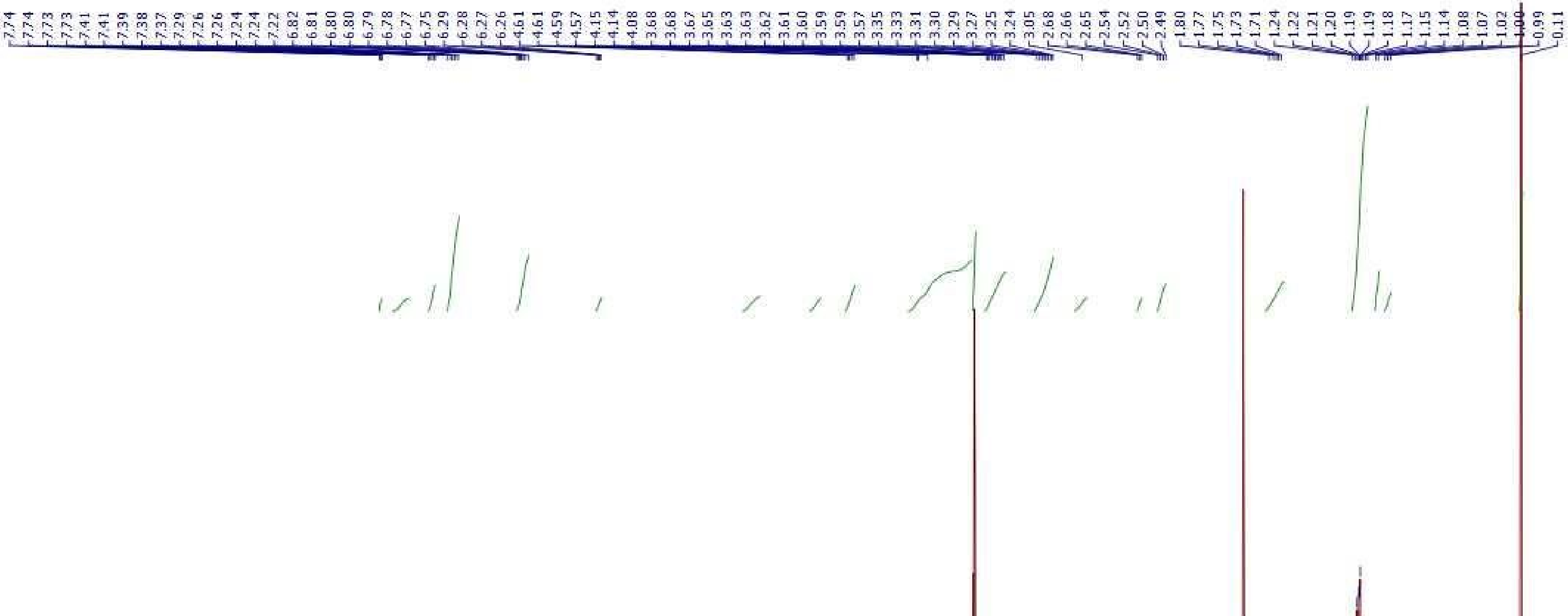
7.74
7.74
7.73
7.73
7.41
7.41
7.39
7.38
7.37
7.29
7.26
7.26
7.24
7.24
7.22
6.82
6.81
6.80
6.80
6.79
6.78
6.77
6.75
6.29
6.28
6.27
6.26
4.61
4.61
4.59
4.57
4.15
4.14
4.08
3.68
3.68
3.67
3.65
3.63
3.63
3.62
3.61
3.60
3.59
3.59
3.57
3.35
3.33
3.31
3.30
3.29
3.27
3.25
3.24
3.05
2.68
2.66
2.65
2.54
2.52
2.50
2.49
1.80
1.77
1.75
1.73
1.71
1.24
1.22
1.21
1.20
1.19
1.19
1.18
1.17
1.15
1.14
1.08
1.07
1.02
1.00
0.99
0.11

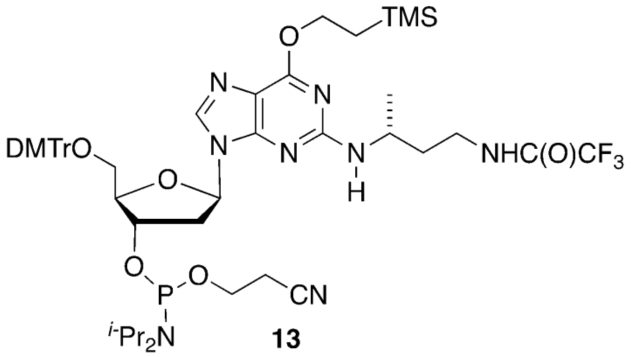
TMS
N
N
O
DMTrO
O
N
N
N
H
NHC(O)CF3
O
P
O
CN
i-Pr2N

13

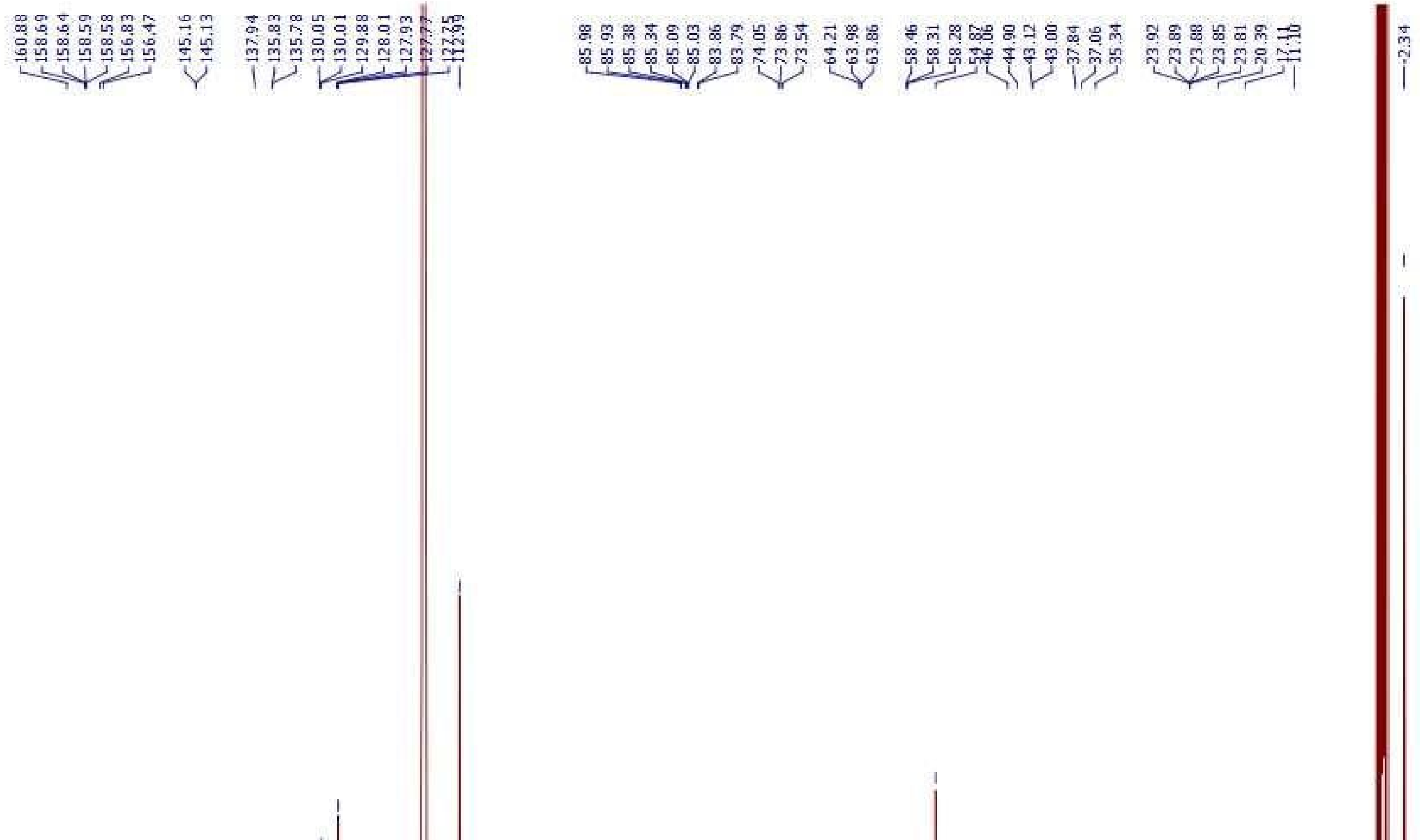
160.88
158.69
158.64
158.59
158.58
156.83
156.47
145.16
145.13
137.94
135.83
135.78
130.05
130.01
129.88
128.01
127.93
127.77
127.75
112.99
85.98
85.93
85.38
85.34
85.09
85.03
83.86
83.79
74.05
73.86
73.54
64.21
63.98
63.86
58.46
58.31
58.28
54.87
46.06
44.90
43.12
43.00
37.84
37.06
35.34
23.92
23.89
23.88
23.85
23.81
20.39
17.11
17.10
-2.34

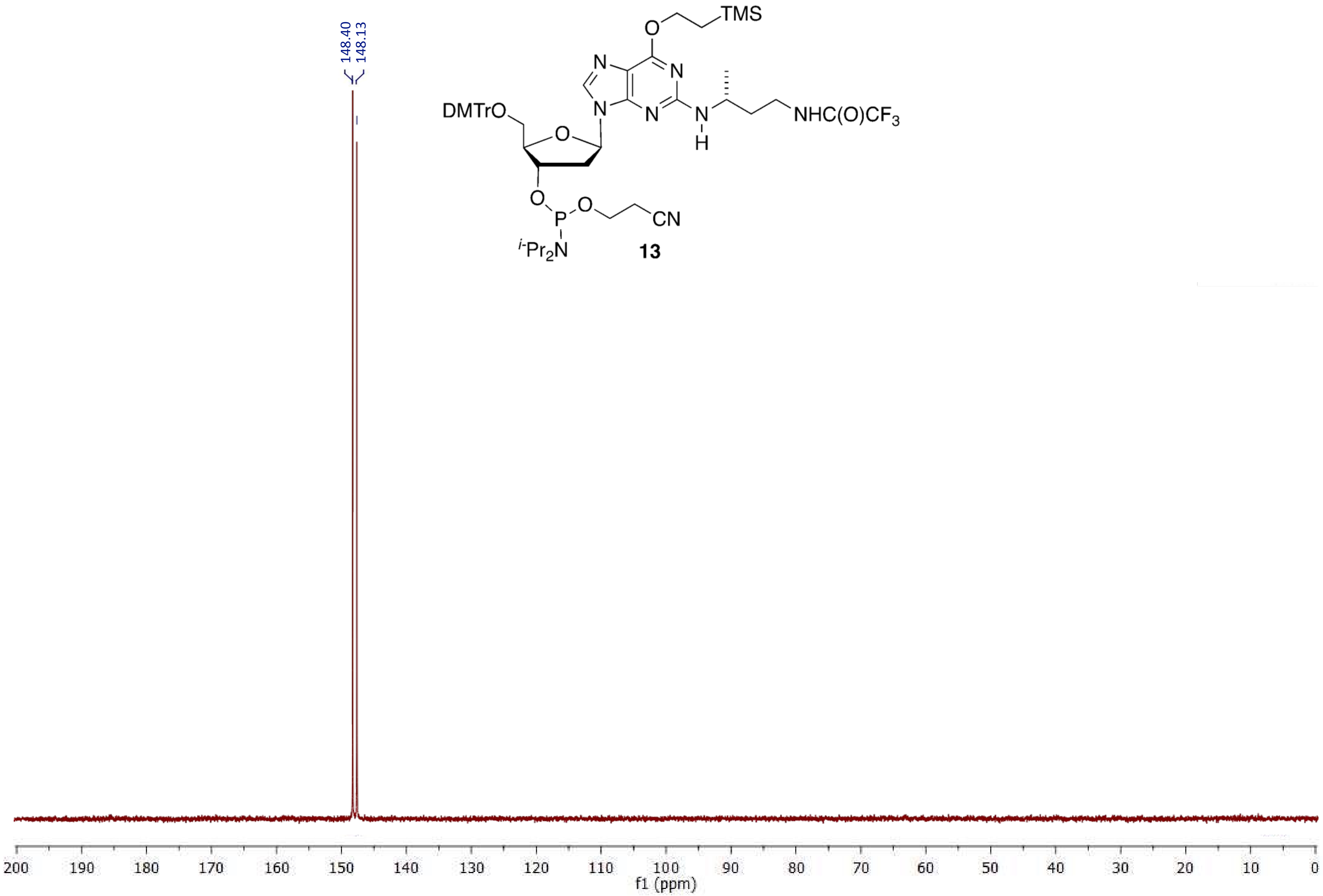
148.40
148.13
DMTrO
TMS
NHC(O)CF3
CN
i-Pr2N
13
200 190 180 170 160 150 140 130 120 110 100 90 80 70 60 50 40 30 20 10 0
f1 (ppm)

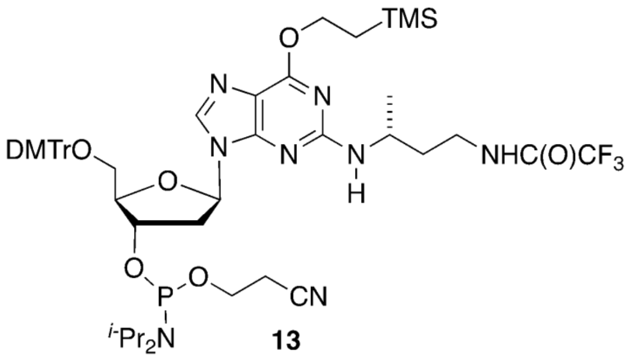

13

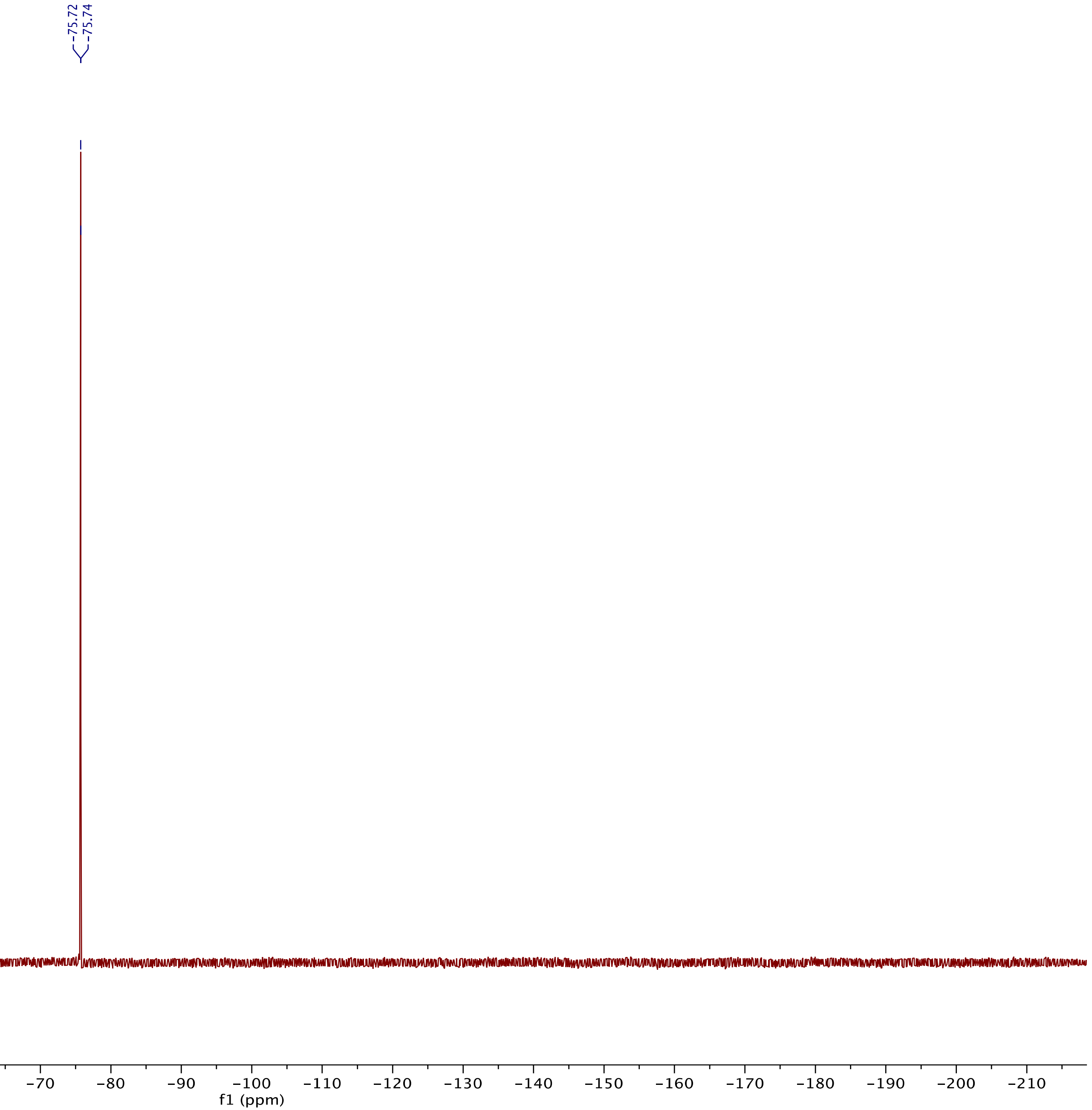

Supplement: Supplementary file 1 — ja2c10070_si_001.pdf [file ja2c10070_si_001.pdf]
